# Identification of Novel Isatin Derivative Bearing a Nitrofuran Moiety as Potent Multi-Isoform Aldehyde Dehydrogenase Inhibitor

**DOI:** 10.3390/molecules29133114

**Published:** 2024-06-29

**Authors:** Krishne Gowda, Asif Raza, Venugopal Vangala, Nazir Ahmad Lone, Jyh Ming Lin, Jaikee Kumar Singh, Sandeep Kumar Srivastava, Todd D. Schell, Gavin P. Robertson, Shantu Amin, Arun K. Sharma

**Affiliations:** 1 Department of Pharmacology, Penn State Cancer Institute, Penn State College of Medicine, Hershey, PA 17033, USA; 2 Department of Biochemistry and Molecular Biology, Penn State Cancer Institute, Penn State College of Medicine Hershey, Hershey, PA 17033, USA; 3 Department of Biosciences, Manipal University Jaipur, Jaipur 303007, Indiasandeepkumar.srivastava@jaipur.manipal.edu (S.K.S.); 4 Department of Microbiology and Immunology, Penn State Cancer Institute, Penn State College of Medicine Hershey, Hershey, PA 17033, USA; 5 Departments of Pathology, Dermatology, Surgery, Melanoma Skin Cancer Center, Penn State Cancer Institute, Penn State College of Medicine Hershey, Hershey, PA 17033, USA

**Keywords:** chemical synthesis, aldehyde dehydrogenases, cancer therapeutics, colon cancer, ovarian cancer, pancreatic cancer, drug development, antioxidant activity, molecular docking

## Abstract

Aldehyde dehydrogenases (ALDHs) are a family of enzymes that aid in detoxification and are overexpressed in several different malignancies. There is a correlation between increased expression of ALDH and a poor prognosis, stemness, and resistance to several drugs. Several ALDH inhibitors have been generated due to the crucial role that ALDH plays in cancer stem cells. All of these inhibitors, however, are either ineffective, very toxic, or have yet to be subjected to rigorous testing on their effectiveness. Although various drug-like compounds targeting ALDH have been reported in the literature, none have made it to routine use in the oncology clinic. As a result, new potent, non-toxic, bioavailable, and therapeutically effective ALDH inhibitors are still needed. In this study, we designed and synthesized potent multi-ALDH isoform inhibitors based on the isatin and indazole pharmacophore. Molecular docking studies and enzymatic tests revealed that among all of the synthesized analogs, compound **3** is the most potent inhibitor of ALDH1A1, ALDH3A1, and ALDH1A3, exhibiting 51.32%, 51.87%, and 36.65% inhibition, respectively. The ALDEFLUOR assay further revealed that compound **3** acts as an ALDH broad spectrum inhibitor at 500 nM. Compound **3** was also the most cytotoxic to cancer cells, with an IC_50_ in the range of 2.1 to 3.8 µM for ovarian, colon, and pancreatic cancer cells, compared to normal and embryonic kidney cells (IC_50_ 7.1 to 8.7 µM). Mechanistically, compound **3** increased ROS activity due to potent multi-ALDH isoform inhibition, which increased apoptosis. Taken together, this study identified a potent multi-isoform ALDH inhibitor that could be further developed as a cancer therapeutic.

## 1. Introduction

Aldehydes are produced through metabolic processes in living organisms from various precursors, including the biosynthetic pathways of amino acids, proteins, carbohydrates, and lipids. The accumulation of aldehydes can lead to cytotoxicity and inflammation, which are closely associated with increased oxidative stress and DNA damage in cells [1,2,3]. More than 200 aldehyde species have been identified, including 4-hydroxy-2-nonenal (4-HNE) and malondialdehyde (MDA), which are produced when cellular membrane lipids undergo oxidative breakdown, a process known as lipid peroxidation [4]. Aldehydes can form adducts with various biological targets, including glutathione (GSH), nucleic acids, and proteins [4]. These adducts can cause DNA damage, disturbed cellular homeostasis, and cell death, and these processes are thought to be the primary mechanisms responsible for aldehydes toxicity [5,6].

The superfamily of aldehyde dehydrogenases (ALDHs) comprises 19 distinct isoenzymes that work with NAD(P)+ as a cofactor to detoxify aldehydes to their corresponding carboxylic acid irreversibly. The various ALDH enzymes participate in several metabolic processes, including detoxifying aldehydes generated from ROS and lipid peroxidation, ethanol consumption, and the metabolism of many neurotransmitters [2,7]. Thus, cancer cells commonly overexpress specific ALDH isoforms to battle oxidative stress caused by high metabolic demands, radiation, and ROS-generating chemotherapeutics [8,9].

Upregulated ALDH isoforms have been linked to cancer progression, resistance, and a poor prognosis in various cancers, including breast, lung, colorectal, esophageal, melanoma, multiple myeloma, neuroblastoma, prostate, and pancreatic cancer [10,11,12,13,14,15,16,17,18,19]. ALDH can be expressed at high levels in cancer cells with a stem-cell-like phenotype. These cells are a small fraction of the multipotent cells constituting tumors and have been reported to be involved in cancer initiation, resistance development, and metastatic spread [20,21]. In addition, the production of retinoic acid by ALDH1A1, ALDH2, and ALDH3 has been reported to be involved in the generation and function of tumor-infiltrating regulatory T cells. These cells contribute to an immunosuppressive tumor microenvironment and inhibit tumor immunity [22]. Most malignancies have increased ALDH expression and activity, which usually worsens the prognosis. ALDH1A and ALDH3A1 have been reported to be critical for cancer development [23,24,25]. ALDH overexpression reduces aldehyde levels in cells and groups of cells to modulate toxic aldehyde-induced oxidative damage, apoptosis, and tumor immunity [9].

**Figure 1:**
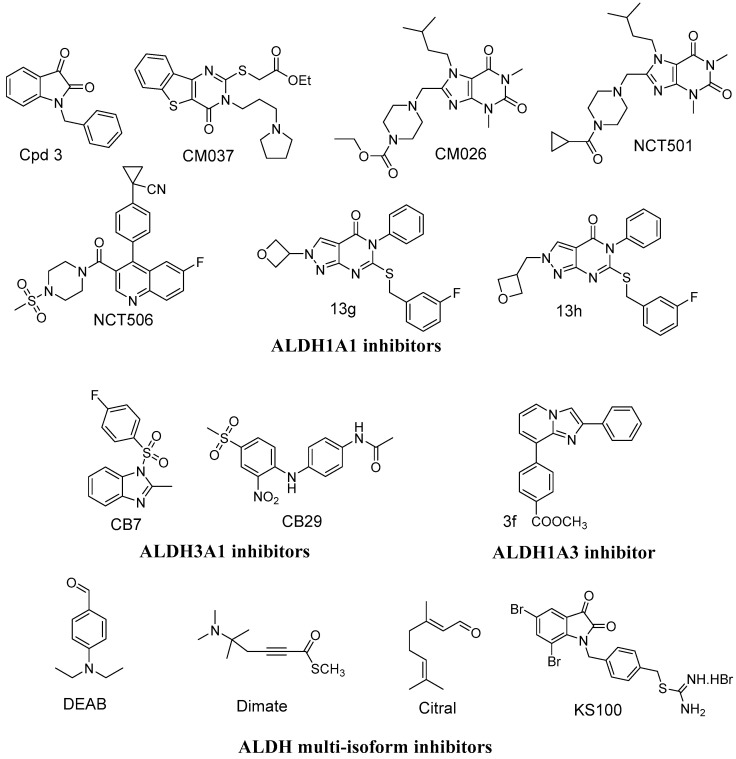
Specific and multi-isoform ALDH inhibitors.

Since ALDH overexpression is known to promote cancer growth and therapeutic resistance, there has been an interest in developing efficient ALDH inhibitors. Current ALDH inhibitors can be divided into two groups: those that predominantly inhibit multiple isoforms and those that specifically inhibit a single isoform [26]. Examples of the isoform-specific inhibitors include the ALDH1A1 inhibitors Cpd 3, CM037, CM026, NCT-501, NCT-505, NCT-506, 13g and 13h; ALDH3A1-specific inhibitors CB7 and CB29 [26,27,28,29,30,31,32,33,34]; and ALDH1A3-specific inhibitor Cpd 3f [35,36,37] (Figure 1). Due to low potency, bioavailability, or overlapping functions with other isoforms, these drugs demonstrate little evidence of in vivo activity. However, some can be effective; for example, NCT-501 reduced HNSCC xenograft development by 78% when injected intratumorally [29]. Since cancer cells often contain many ALDH isoforms, targeting multiple isoforms may be necessary for the best anticancer therapy. *N,N*-Diethylamino benzaldehyde (DEAB), 4-dimethylamino-4-methyl-pent-2-ynthioic acid-S-methyl ester (DIMATE), citral, (Aldis)-1, 2, 3, 4 and 6, and dyclonine are examples of compounds that block multiple ALDH isoforms [26,38,39,40,41,42,43,44,45,46] (Figure 1). However, their effectiveness might depend on which ALDH isoforms are inhibited. This can also lead to unexpected effects; for example, DIMATE’s oral bioavailability is uncertain, and citral has off-target effects and can be toxic [38,40,41,42,43,47]. Although various drug-like compounds targeting ALDH have been reported in the literature, none have made it to routine use in the oncology clinic. As a result, new, potent, non-toxic, bioavailable, and therapeutically effective ALDH inhibitors are still needed.

We recently developed KS100, a multi-isoform ALDH inhibitor bearing an isatin core structure, as a promising drug candidate to treat melanoma [48,49]. Systemic toxicity was observed with KS100 when given i.p. at a dose of 5 mg/kg per day, which led to the development of its liposomal nanoformulation (nano-KS100), which, at 20 mg/kg/day intravenous dose, suppressed tumor growth by 65% without any obvious toxic effects [49]. During our ongoing investigation into the isatin analog KS100 and its potential application as a multi-isoform ALDH inhibitor, we recently developed a renewed interest in synthesizing novel isatin analogs (**1**–**7**) to enhance efficacy further while reducing toxic effects. Besides using the bio isostere hypothesis, we also chose indazole as a novel scaffold to replace the isatin pharmacophore (**8**–**21**).

Herein, we report the synthesis and pharmacological assessment of new isatin and indazole-based ALDH inhibitors against ALDH1A1, ALDH1A3, and ALDH3A1. We extended these compounds’ structure–activity relationship (SAR) primarily using a bio-isosteric approach in which we substituted the isatin and indazole rings at the C3, C5, or C7 positions and the N-benzyl moiety. We also examined their antiproliferative efficacy in cancer and normal cells and their efficacy for inhibiting particular ALDH isoforms.

## 2. Results and Discussion

### 2.1. Molecular Docking Studies 

Since overall ALDH activity within cancer cells is mediated by several ALDH isoforms, multi-ALDH isoform inhibition may be more beneficial for cancer therapy than using isoform-specific agents [50,51,52]. One of the most effective ALDH1A1 selective inhibitors is Cpd 3, an isatin analog [27]. Previously, we reported KS100, also an isatin analog, as an effective non-selective ALDH inhibitor suppressing tumor growth [48,49] by inhibiting multiple ALDH isoforms with an IC_50_ of 230 nM, 1.54 µM, and 193 nM for ALDH1A1, ALDH2, and ALDH3A1, respectively. However, in the naked form, it was toxic to mice at 5 mg/kg/day. Consequently, we used molecular docking studies to improve the structure of KS100 to generate new, efficient multi-ALDH isoform inhibitors with reduced toxicity.

A series of compounds (**1**–**21**) were designed and assessed using molecular docking studies for capacity to bind in the active site pockets of ALDH1A1, ALDH1A3, and ALDH3A1. Autodock Vina was used to perform molecular docking because of its accuracy in repeatedly predicting the poses of the ligands at the binding sites, with lower RMSD values for poses that are close to the crystal structure and have the highest binding affinity. Vina is highly robust in reproducing the same pose as that ligand in crystal structures. 1,4-Bis(bromomethyl)benzene was used as a linker to connect the isatin, indazole scaffolds, and other moieties. 

**Figure 2:**
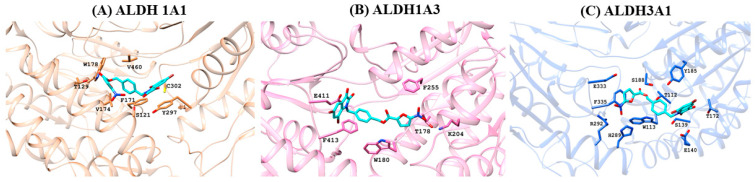
Molecular docking 3D representation of compound **3** in the active site pockets of (**A**) ALDH1A1 (PDB ID: 4 × 4 L) (**B**) ALDH1A3 (PDB ID: 7QK7) (**C**) ALDH3A1 (PDB ID: 3SZB).

The compounds were initially docked in silico into the ligand-binding pockets of ALDH1A1, ALDH1A3, and ALDH3A1. The docking scores of all of the compounds (**1**–**21**) are depicted in Table 1. To investigate the ligand binding pocket of the ALDH isoforms, we chose compound **3** as a representative molecule and performed molecular docking experiments on the ALDH1A1, ALDH1A3, and ALDH3A1 protein structures (Figure 2). The docking scores of ALDH1A1, ALDH1A3, and ALDH3A1 with compound **3** are −10.1 kcal/mol, −9.6 kcal/mol, and −10.4 kcal/mol, respectively. The docking scores indicate strong binding of ligands and a good fit into the ligand-binding pockets of ALDH1A1, ALDH1A3, and ALDH3A1. Docking studies with ALDH1A1 revealed π−π interactions with the W178 residue, H-bond interactions with Ser121 residues, and Cys302 residues (Figure 2). Similar docking studies with compound **3** and the protein structures of ALDH1A3 and ALDH3A1 showed hydrophobic interaction with Phe255, π-stacking with Phe413, and hydrogen bonds with Thr178, Trp180, Lys204, and Glu411 occurred between the ligand and ALDH1A3. Similarly, ALDH3A1 and the ligand formed π−π interactions with Thr112 and Phe335, hydrophobic interactions with Thr172, and hydrogen bonds with Thr172 and Arg292. Docking scores for compounds (**1**–**21**) varied between −7.9 to −10.1 for ALDH1A1, −7.2 to −9.6 for ALDH1A3, and −7.2 to −10.4 for ALDH3A1 (Table 1). Based on these impressive docking scores, compounds (**1**–**21**) were synthesized to examine their ALDH enzyme inhibitory activity, anticancer effectiveness, and toxicity.

### 2.2. Chemistry

**Scheme 1:**
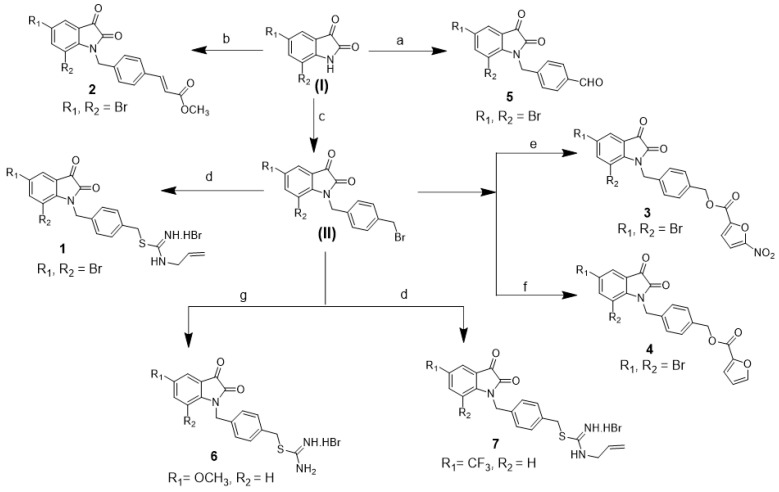
Synthesis of novel isatin derivatives (**1**–**7**). Reagents and conditions: a) K_2_CO_3_, DMF, 4-(bromomethyl)benzaldehyde, RT; b) K_2_CO_3_, DMF, methyl (2E)-3-[4-(bromomethyl)phenyl]-2 propenoate, RT; c) K_2_CO_3_, DMF, 1,4-bis(bromomethyl)benzene, RT; d) *N*-allylthiourea, EtOH, reflux; e) Cs_2_CO_3_, DMF, 5-nitro-2-furoic acid, RT; f) Cs_2_CO_3_, DMF, 2-furoic acid, RT; g) thiourea, EtOH, reflux.

The synthesis of isatin derivatives (**1**–**7**) is depicted in Scheme 1. The key intermediates, N-(*p*-bromomethyl benzyl)isatins (II), were synthesized in a single step by reacting 5- or 5,7-disubstituted isatins with 1,4-bis(bromomethyl)benzene in DMF in the presence of potassium carbonate. The intermediates (II) were subsequently refluxed in ethanol with allylthiourea to produce the corresponding compounds **1** and **7** in excellent yield in the range 69−76% and with thiourea in ethanol to yield compound **6**. When the intermediate (II) was agitated with 5-nitrofuran-2-carboxylic acid or furan-2-carboxylic acid in cesium carbonate and DMF, compounds **3** and **4** were obtained in good yields in the range 71–73%. Compounds **2** and **5** were produced in satisfactory amounts in the range 56–68% when methyl (*E*)-3-(4-(bromomethyl)phenyl)acrylate or 4-(bromomethyl)benzaldehyde was reacted with substituted isatin (I) in DMF (Scheme 1). 

**Scheme 2:**
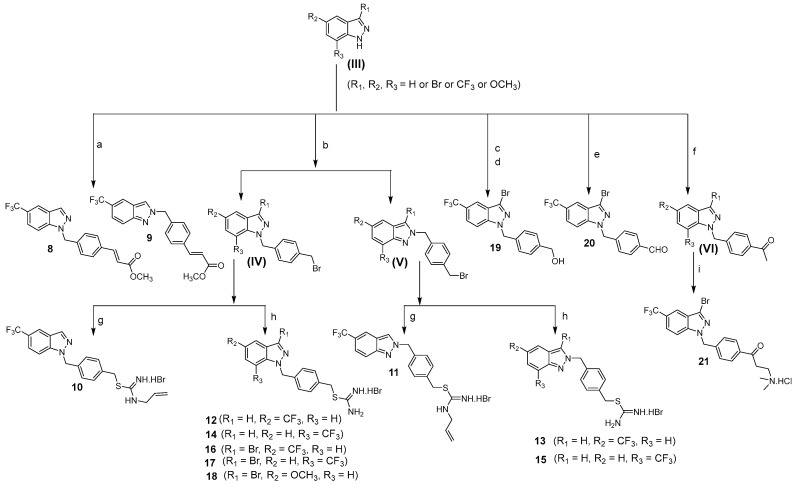
Synthesis of novel indazole derivatives (**8**–**21**). Reagents and conditions: (a) NaH, DMF, 60 °C, methyl (2E)-3-[4-(bromomethyl)phenyl]-2-propenoate; (b) NaH, DMF, 60 °C, 1,4-bis(bromomethyl)benzene, RT; (c) NaH, DMF, 60 °C, 4-(bromomethyl)benzyl)oxy)TBDMS; (d) THF, TBAF, RT; (e) NaH, DMF, 60 °C, 4-(bromomethyl)benzaldehyde; (f) NaH, DMF, 60 °C,1-(4 (Bromomethyl)phenyl)ethanone; (g) *N*-allylthiourea, EtOH, reflux; (h) thiourea, EtOH, reflux; (i) Paraformaldehyde, dimethyl amine, EtOH, RT.

Indazole derivatives (**8**–**21**) were synthesized as outlined in Scheme 2. Compounds **8** and **9** were generated in decent yields in the range of 26−40% when methyl (*E*)-3-(4-(bromomethyl)phenyl)acrylate was reacted in DMF with substituted indazole (III). The location of the side chain was verified by an HMBC NMR experiment (Figures S3 and S4). The regio-isomer structure **9** was determined by assigning all of the chemical shifts of carbon, as indicated in Table 2. The H_10_ proton interacted with carbons at δ = 127.47 (C_1_), 128.77 (C_12_) and 139.27 (C_11_). If the side chain was at the N_3_-position, H_10_ would not interact with C_1_ carbon. The HMBC NMR experiment also confirmed the substitution of N_3_ regio-isomer **8**. The H_10_ proton was observed to interact with carbons δ = 140.82 (C_9_), 139.9 (C_11_), and 128.3 (C_12_).

The syntheses of intermediates (IV) and (V) were accomplished by treating substituted indazoles (III) with 1,4-bis(bromomethyl)benzene in DMF in the presence of sodium hydride. The intermediates (IV) and (V) were refluxed with allylthiourea in ethanol to obtain compounds **10** and **11** in good yield in the range 46−61%. Refluxing (IV) and (V) with thiourea in ethanol under similar conditions yielded compounds **12**, **14**, **16**, **17**, **18**, **13**, and **15**. Compound **19** was obtained by reacting compound (III) with 4-(bromomethyl)benzyl)oxy)TBDMS in DMF, followed by treatment with tetrabutylammonium fluoride in THF. Under identical conditions, compound **20** was generated by reacting compound (III) with 4-(bromomethyl)benzaldehyde in a decent yield (56%). Compound **21** was synthesized in good yield (62%) by the reaction of 1-(4-(bromomethyl)phenyl)ethan-1-one with indazole (III), which resulted in the generation of the intermediate (VI), followed by the treatment of this intermediate with paraformaldehyde and hydrochloric acid. ^1^H NMR, ^13^C NMR, and HRMS analysis confirmed the structures of all isatin and indazole derivatives. The compound purity (≥98%) was established by analytical high-performance liquid chromatography (HPLC) before proceeding with the in vitro biological assays.

### 2.3. Biological Evaluation

#### 2.3.1. Cytotoxicity

The efficacy of all of the synthesized compounds was assessed in normal and cancer cell lines. The synthesized compounds (**1**–**21**) were evaluated for their antiproliferative activity against SKOV-3, MiaPaCa-2, HCT-116, HEK-293, and FHC cell lines, and KS100, previously developed as a multi-ALDH isoform inhibitor in our laboratory, was used as the reference standard (Table 3; dose-response in, Figures S1 and S2). Different substituents at positions 3, 5, and 7 were introduced into the isatin and indazole rings and different functionalities to the benzyl moiety to determine the structure–activity relationship. Compounds **1**, **3**, and **16** in the series demonstrated promising cytotoxicity, with IC_50_ values ranging from 2.1 to 6.8 µM against the tested cell lines. Compounds **1** and **3** are dibromo-substituted isatin analogs, whereas compound **16** is an indazole derivative with CF_3_ and bromo substituents. 

Compared to the other compounds in the series, compound **3** displayed a much higher potency level as an inhibitor of ALDH1A1, ALDH1A3, and ALDH3A1 (Table 3 and Table 4). It showed a comparatively lower level of toxicity against normal cells (FHC and HEK293). The addition of a nitro group to the furan moiety boosted the inhibition of ALDH enzymes (namely ALDH1A1 and ALDH3A1) two-fold over compound **4** with no nitro moiety, as well as boosting its antiproliferative effects on cancer cell lines. Compound **4**, which has a furan moiety but lacks a nitro group, exhibited a lower degree of cytotoxicity than compound **3**, interpreted as the nitro group contributing to the antiproliferative activities on the cancer cell lines. Compared to compound **1**, compound **2** lost activity, requiring >20 µM for activity when the benzyl moiety was combined with an acrylate ester. Compounds **5** and **7** were less cytotoxic than KS100, whereas compound **6** was inert, suggesting the importance of dibromo and isothiourea moieties in modulating cellular proliferation.

After that, the indazole compounds were tested for their antiproliferative properties in several cancer cell lines (Table 3). Compounds **8**, **9**, **10**, **11**, **15**, **17**, **18**, **19**, **20**, and **21** were ineffective for killing cancer cells until concentrations greater than 50 µM were used. Compounds **12** and **13** had an IC_50_ ranging from 4.7 to 33 µM, while compound **16** was a little more potent than the other indazole derivatives in all three cancer cell lines. The CF_3_ functionality alone did not play a significant role in causing toxicity, while indazole derivative **16** with CF_3_ and bromo substitution was more cytotoxic to cancer cells. When isatin and indazole derivatives are compared, isatin derivatives are more cytotoxic than indazole derivatives.

#### 2.3.2. ALDEFLUOR Assay and ALDH Isoform Inhibitory Activity

**Figure 3:**
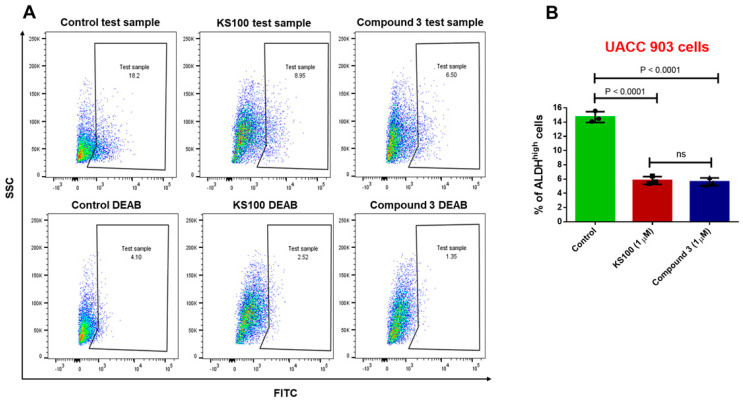
Inhibition of ALDH Activity in Human Melanoma by Compound **3** and KS100. ALDEFLUOR assay using DEAB as a negative control. UACC 903 cells treated with Compound **3** and KS100 were stained with ALDEFLUOR dye (**A**), and the resulting bar graph represents triplicates (**B**). Both compounds exhibited significant inhibition of ALDH activity, suggesting their potential therapeutic relevance in human melanoma. The bar graph displays the mean ± SD of three replicates. Statistical analysis was conducted using one-way ANOVA with GraphPad PRISM Version 6 software, followed by Tukey’s multiple comparisons test. Statistical significance was established at a *p*-value of < 0.05.

Based on the cytotoxicity data, we selected compounds **1**, **3**, **4**, **5**, **12**, **16**, and **17** from among the 21 compounds, and **KS100** as a positive control to define their inhibitory actions against ALDH1A1, ALDH3A1, and ALDH1A3 enzyme activity at 500 nM concentration and the results are given in Table 4. The inhibition of enzymes was assessed by measuring the conversion of NAD+ to NADH in the presence of **1**, **3**, **4**, **5**, **12**, **16**, **17**, and **KS100** after adding isoform-specific aldehydes. ALDH1A1 inhibitory activity ranged from 9.46 to 51.32%, ALDH3A1 inhibitory activity ranged from −25.33 to 51.87%, and ALDH1A3 inhibitory activity ranged from 13.83 to 36.65 (Table 4). The most effective multi-ALDH isoform inhibitor was compound **3**, with 51.32%, 51.87%, and 36.65% enzyme inhibition activity for ALDH1A1, ALDH3A1, and ALDH1A3 (Table 4). To investigate whether compound 3 acts as a broad-spectrum ALDH inhibitor, we conducted an ALDEFLUOR assay using **KS100** as a positive control. The results showed a significant (~2.5-fold) inhibition of overall ALDH activity in melanoma cells by both **KS100** and compound **3** (Figure 3A,B).

The structure–activity relationship of compounds **1**, **3**, **4**, **5**, **12**, **16**, and **17** was analyzed and compared to **KS100** (Table 4). In general, the multi-ALDH isoform inhibitory action of compounds **1**, **3**, **4**, and **5** with a dibromo isatin pharmacophore was shown to be higher than that of the indazole compounds. Compound **3** was an ALDH inhibitor and more effective than KS100 at inhibiting several isoforms. When compared to **KS100** inhibitory activity, the ALDH inhibitory activity of **3** was 51.32% for ALDH1A1, 51.87% for ALDH3A1, and 36.65 for ALDH1A3, whereas the **KS100** inhibitory activity was 45.93% for ALDH1A1, 37.04% for ALDH3A1, and 13.83 for ALDH1A3. In comparison to the isothiourea moiety present in **KS100**, the nitrofuran moiety in compound **3** led to better inhibition of ALDH1A1, ALDH3A1, and ALDH1A3. Compound **3** contains 5,7-dibromo isatin with a nitrofuran moiety and seems to confer the compound with broad-spectrum ALDH inhibitory activity by providing it with a more hydrophobic character due to the bromo isatin moiety and the interaction of nitrofuran with the active site of ALDH enzymes. Among all of the compounds, 5,7-dibromo isatin with nitrofuran moiety is the structure that ultimately leads to broad-spectrum ALDH inhibitory activity.

#### 2.3.3. Evaluation of the Antiproliferative Activity of Compound **3** (MTS and TBDEA)

**Figure 4:**
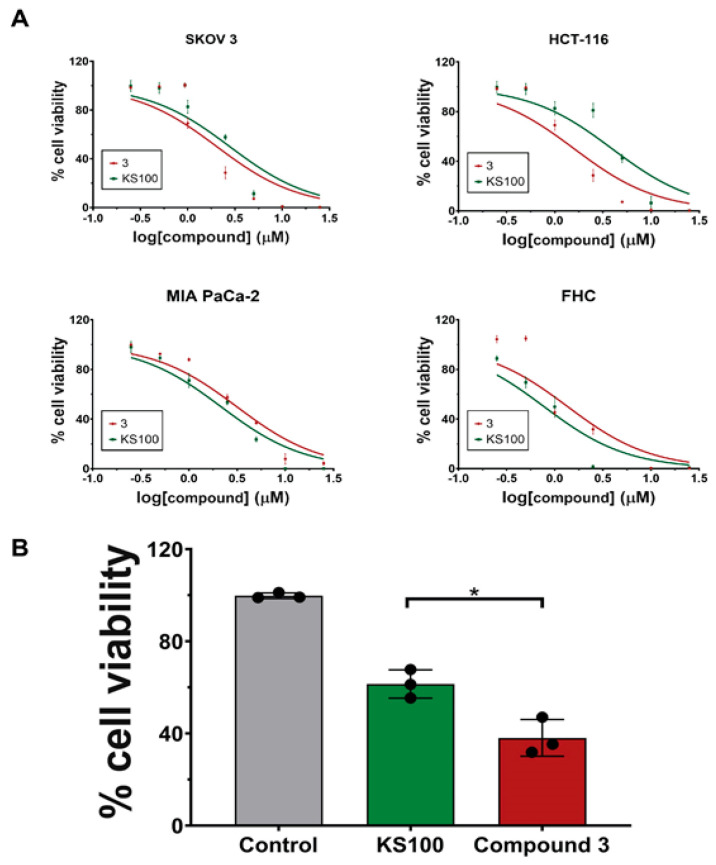
(**A**) HCT-116, SKOV-3, MIA PaCa-2, HEK-293, and FHC cell lines were treated with compound **3** (**KS124**) and **KS100** for 48 h at different concentrations. At the end of the treatment duration, an MTS assay was performed. The data were analyzed on GraphPad V 8.0 software (Prism, CA, USA) using non-linear regression. (**B**) Trypan blue dye exclusion assay–HCT-116 cells were treated with 3 µM of compounds 3 (**KS124**) and **KS100** for 48 h. The cells were harvested and stained with trypan blue. The live cells (excluded trypan blue) and dead cells (stained with trypan blue) were counted using a hemocytometer. Data are presented as mean ± SD; *n* = 3 for each treatment with * *p* < 0.05.

As shown in Figure 4A, compound **3** inhibited the in vitro growth of the human cancer cell lines in a concentration-dependent manner as measured by the MTS assay. Out of all of the **21** compounds screened, compound **3** showed better killing efficacy compared to the reference compound **KS100**. Compound **3** had IC_50_ values of 2.2 ± 0.2, 3.8 ± 0.4, 2.1 ± 0.1, 8.7 ± 1.1, and 7.1 ± 0.8 while **KS100** had IC_50_ values of 3.8 ± 0.8, 2.8 ± 0.2, 4.3 ± 0.7, 4.3 ± 0.1, and 4.9 ± 0.8 against SKOV-3, MiaPaCa-2, HCT-116, HEK-293, and FHC cell lines, respectively. Compound **3** had significantly greater effectiveness, ranging from 0.5 to 2 times, than KS100 in targeting cancer cells. Additionally, it was found to be twice as non-toxic toward normal cell lines, as shown by the cell viability assay (Figure 4A, Table 3), suggesting a more significant therapeutic index. A trypan blue dye exclusion assay was performed using HCT-116 cells to validate the data obtained from the MTS assay further. HCT-116 cells were treated with 3 µM of compound **3** or **KS100** for 48 h, harvested, and stained with trypan blue. The live (excluded trypan blue) and dead (stained with trypan blue) cells were counted using a hemocytometer. Compound **3** exhibited 23% higher cytotoxicity than KS100 when both were present at a concentration of 3 µM for a treatment duration of 48 h, as shown in Figure 4B. Thus, the trypan blue dye exclusion assay data confirmed the findings of the cell viability assay using MTS, indicating improved killing of cancer cells.

#### 2.3.4. Compound **3** Induced Caspase-Mediated Apoptotic Cell Death in HCT116 Cells

**Figure 5:**
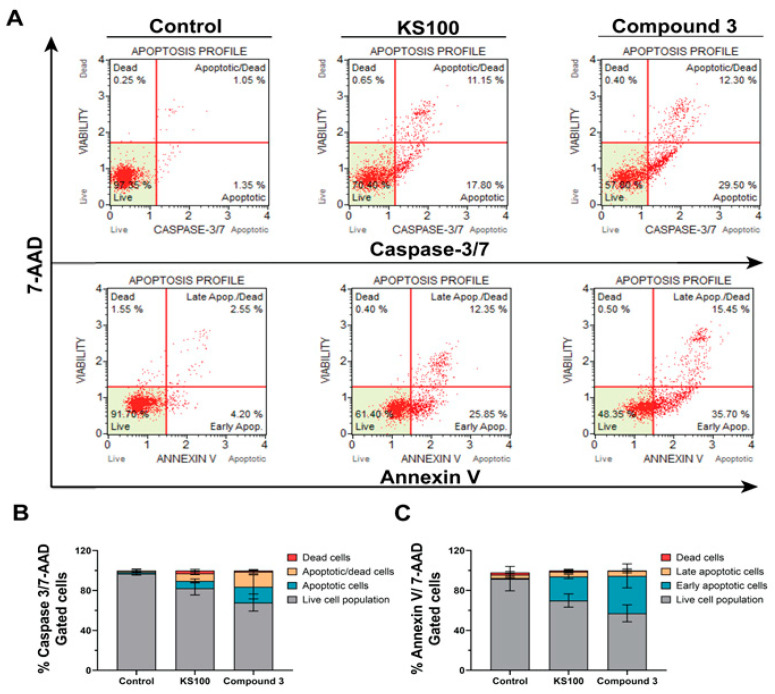
(**A**) HCT-116 cells were treated with compound **3** (**KS124**) and **KS100** 24 h (Caspase 3/7 assay) and 48 h (Annexin V/7-AAD assay). (**B**,**C**) represent the quantification of the apoptotic cell population after the Caspase 3/7 assay and Annexin V/7-AAD assay, respectively. Data are presented as Mean ± SEM; *n* = 3 for each treatment.

Among all of the 21 compounds, compound **3** emerged as a lead molecule with the most potential for killing cancer cells. We evaluated cell death using the apoptosis assays caspase-3/7 7-AAD and Annexin V/7-AAD to identify the mechanistic basis for this efficacy. Both procedures were used to determine the live and apoptotic cell populations [54]. Compound **3** was more effective than **KS100** in inducing apoptosis, even at a similar dose (3 µM), according to a Muse-based analysis of HCT-116 cells treated with both compounds (Figure 5A). Compound **3** showed ~12.8% (*p* = 0.0039) and ~13% (*p* = 0.0376) more apoptotic cell populations than KS100 in caspase-3/7 7-AAD and Annexin V/7-AAD, respectively. A quantitative comparison of the apoptotic cell populations caused by compound **3** and **KS100** is shown in Figure 5B,C.

#### 2.3.5. Compound **3** Generates more ROS Than KS100

**Figure 6:**
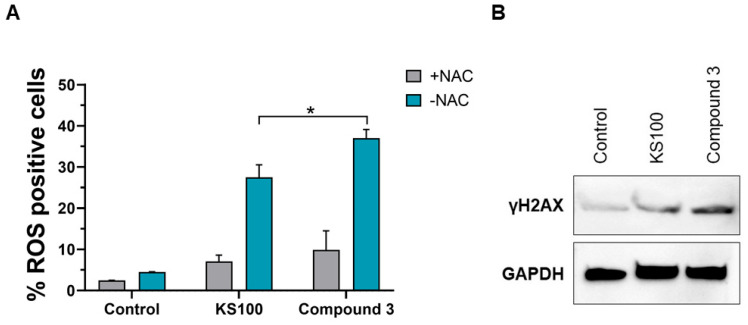
(**A**) The generation of ROS in HCT-116 cells was assessed by using the Muse oxidative stress kit. Untreated HCT-116 cells were taken as the control, and cells were treated in the presence or absence of the antioxidant NAC (10 mM). HCT-116 cells were treated with 3 µM of compound **3** (**KS124**) and KS100 for 24 h. Data are presented as mean ± SD; *n* = 3 for each treatment at each time point. * *p* < 0.05 compared to **KS100** treated cells. (**B**) Western blot demonstrating increased γH2AX protein levels in HCT-116 cell lines treated with 3 µM of compound **3** and **KS100** for 12 h.

Increased ROS production and antioxidant depletion can cause cancer cells to undergo apoptosis [55]. ROS production in the HCT-116 cells was analyzed using an oxidative stress kit after treating the cells for 24 h with compounds **3** or **KS100** with/without *N*-acetylcysteine (NAC) for 24 h to quantify the ROS levels and its potential role in inducing apoptosis. NAC is a thiol-containing compound that has been used as an antioxidant to demonstrate the role of ROS in apoptosis. The results suggest compound **3** produces 34.7% (*p* = 0.0013) more ROS than **KS100** (Figure 6A). When combined with NAC (a ROS scavenger)[56], both compounds showed a reduction in ROS production. These data suggest ROS may affect HCT-116 cells’ subsequent apoptosis-mediated cell death. Consistent with these data, western blot analysis confirmed DNA damage initiation by these compounds with the induction of γH2AX in HCT-116 cells (Figure 6B). The increased expression of γH2AX within 12 h of treatment suggests both compounds initiate DNA damage.

## 3. Materials and Methods

### 3.1. Chemistry

Isatin was purchased from Sigma–Aldrich (Sigma–Aldrich, St. Louis, MO, USA), and previously published procedures (41) were used to synthesize 5,7-dibromo isatin. Indazoles were purchased from Combi Blocks (San Diego, CA, USA). All of the other chemicals and solvents were purchased from major suppliers. As received, anhydrous solvents were employed. Glassware that had been dried in a nitrogen environment was used for the reactions. Analytical thin-layer chromatography (TLC) was used to evaluate the course of the reaction on aluminum-backed, precoated silica gel 60 F254 plates (Sigma–Aldrich). Colorless compounds were found using UV light and/or iodine vapor. Silica gel 60 (230–400 mesh, E. Merck, Rahway, NJ) was used for column chromatography together with the solvent system described in each method. Vol/Vol is used to express all solvent ratios. Utilizing a Bruker Avance 500 MHz spectrometer (Billerica, MA, USA), NMR spectra were captured. Chemical shifts (δ) from the internal standard were reported in parts per million downfield. The signals are denoted by the abbreviations s (singlet), d (doublet), t (triplet), m (multiplet), dd (doublet of doublet), respectively. The residual solvent peak of the solvent mentioned in each process is the standard by which spectra are measured. MS spectra were recorded in positive-ion mode on TripleTOF 5600 (AB Sciex, Framingham, MA, USA) mass spectrometer with electrospray ionization (ESI). The samples were analyzed by flow infusion with a Prominence UFLC system (Shimadzu, Columbia, MD, USA) at flow 300 μL/min rates of 0.1% formic acid (MS grade) in a mixture of methanol and water 60:40. MS1 and MS2 data were acquired using a declustering potential (DP) of 100 V. MS2 data were obtained for a collision energy (CE) of 35 V with a collision energy spread (CES) of 15 eV. During the analysis, an ion spray voltage (IS) of 5500  V, curtain gas (CUR) of 35 psi, nebulizer gas (GS1) of 50 psi, heater gas 2 (GS2) of 55, and heater temperature (TEM) of 550 C were applied. The melting points were calculated using an uncorrected Fischer–Johns melting point equipment. Unless otherwise noted, all substances have a purity of >97% as determined by HPLC using an HP-Agilent 1200 HPLC machine (Santa Clara, CA, USA) on a C18 column. ALDH1A1 and ALDH3A1 were purchased from R&D systems, and ALDH1A3 from Sino Biological United States. The substrate and substrate mixture used were supplied with an ALDH assay Kit (MK-085) purchased from Sigma–Aldrich (St. Louis, MO, USA).

#### Procedure for the Synthesis of Compounds (**1**–**21**)

4-((5,7-Dibromo-2,3-dioxoindolin-1-yl)methyl)benzyl allylcarbamimidothioate hydrobromide (**1**)

Thiourea (47.6 mg, 0.41 mmol), ethanol (10 mL), and 5,7-dibromo-N-(*p*-bromomethylbenzyl)isatin (II) (200 mg, 0.41 mmol) were then added and heated to reflux until the initial substance disappeared (TLC). Under vacuum, the solvent was evaporated. To eliminate unreacted 5,7-dibromo-*N*-(*p*-bromomethylbenzyl)isatin, the crude product was washed with ethylacetate. To obtain orange-yellow crystals of 1, the product was recrystallized using ethanol and ethyl acetate (1:3), Yield: 76%; mp: 118–120 °C; ^1^H NMR (500 MHz, DMSO-*d*_6_): δ = 9.85 (br. s, 1H), 9.44 (br s, 1H), 9.12 (br s, 1H), 8.01 (d, *J* = 1.99 Hz, 1H), 7.82 (d, *J* = 1.99 Hz, 1H), 7.40 (d, *J* = 8.37 Hz, 2H), 7.37 (d, *J* = 8.37 Hz, 2H), 5.78~5.71 (m, 1H), 5.24 (s, 2H), 5.15 (d, *J* = 10.99 Hz, 1H), 5.06 (d, *J* = 17.65 Hz, 1H), 4.53 (s, 2H), 3.94 (d, *J* = 4Hz, 2H). ^13^C NMR (125 MHz, DMSO-*d*_6_): δ = 181.06, 165.63, 159.57, 146.64, 143.70, 137.37, 134.33, 131.68, 129.52, 127.08, 126.79, 123.26, 117.73, 116.17, 104.59, 45.92, 44.41, 34.99. MS (ESI) m/z 524 [M+H]; HRMS (ESI) m/z for C_20_H_17_Br_2_N_3_O_2_S calculated 523.2430, found m/z: 523.9405.

Methyl (*E*)-3-(4-((5,7-dibromo-2,3-dioxoindolin-1-yl)methyl)phenyl)acrylate (**2**)

5,7-Dibromoisatin (I) (200 mg, 0.66 mmol) was taken up in anhydrous DMF (5 mL), and stirred with K_2_CO_3_ (113 mg, 0.82 mmol) at RT for 1 h. Methyl (2*E*)-3-[4-(bromomethyl)phenyl]-2-propenoate (210 mg, 0.82 mmol) was added, and the reaction mixture was stirred for 4 h until the 5,7-dibromoisatin starting material had been consumed (TLC). The reaction mixture was poured into cold water and extracted with ethyl acetate. The ethyl acetate layer was washed with water, brine and dried over MgSO_4_. The solvent was removed, and the crude product was purified by silica gel column chromatography (hexane:ethylacetate; 80:20) as eluent to obtain **2** as an orange yellow solid, yield: 56%; mp: 186–188 °C; ^1^H NMR (500 MHz, DMSO-*d*_6_): δ = 8.00 (d, *J* = 1.99 Hz, 1H), 7.81 (d, *J* = 1.99 Hz, 1H), 7.70 (d, *J* = 8.34 Hz, 2H), 7.66 (d, *J* = 16.24 Hz, 1H), 7.45 (d, *J* = 8.34 Hz, 2H), 6.65 (d, *J* = 16.24 Hz, 1H), 5.27 (s, 2H), 3.73 (s, 3H). ^13^C NMR (125 MHz, DMSO-*d*_6_): δ = 181.00, 167.19, 159.62, 146.61, 144.72, 143.64, 140.17, 133.30, 128.99, 127.20, 126.72, 123.32, 118.01, 116.13, 104.61, 51.92, 44.60. MS (ESI) m/z 480[M+H]; HRMS (ESI) m/z for C_19_H_13_Br_2_NO_4_ calculated 479.1240, found m/z: 479.9278.

4-((5,7-Dibromo-2,3-dioxoindolin-1-yl)methyl)benzyl 5-nitrofuran-2-carboxylate (**3**)

A suspension of compound (II) (250 mg, 0.51 mmol) and solid Cs_2_CO_3_ (208 mg, 0.64 mmol) was agitated with 5-nitro-2-furoic acid (80 mg, 0.51 mmol) overnight in anhydrous DMF (8 mL). The reaction mixture was poured into water and extracted with ethyl acetate. After being washed with water and brine, the ethyl acetate layer was dried over MgSO_4_. The crude product was purified using silica gel column chromatography with hexane:ethylacetate (80:20) as the eluent to provide **3** as a yellow solid, yield: 71%; mp: 122–124 °C; ^1^H NMR (500 MHz, DMSO-*d*_6_): δ = 8.01 9d, J = 1.92 Hz, 1H), 7.81 (d, *J* = 1.92 Hz, 1H), 7.78 (d, J = 3.87 Hz, 1H), 7.63 (d, J = 3.87 Hz, 1H), 7.44 (s, 4H), 5.39 (s, 2H), 5.27 (s, 2H). 13C NMR (125 MHz, DMSO-*d*_6_): δ = 181.08, 159.58, 157.12, 146.67, 144.37, 143.72, 137.90, 134.28, 129.15,126.91, 126.76, 123.24, 120.66, 116.13, 113.51, 104.65, 67.42, 44.47. MS (ESI) m/z 587 [M+Na]; HRMS (ESI) m/z for C_21_H_12_Br_2_N_2_O_7_ calculated 564.1420, found m/z: 586.8907.

4-((5,7-Dibromo-2,3-dioxoindolin-1-yl)methyl)benzyl furan-2-carboxylate (**4**)

The procedure used for 3 was repeated with compound (II) (200 mg, 0.41 mmol), Cs_2_CO_3_ (167 mg, 0.51 mmol), and 2-furoic acid (80 mg, 0.51 mmol) in anhydrous DMF (8 mL). The compound **4** was an orange solid, yield: 73%; mp: 132–134 °C; ^1^H NMR (CDCl_3_) δ = 7.84 (d, *J* = 1.99 Hz, 1H), 7.74 (d, *J* = 1.99 Hz, 1H), 7.60 (m, 1H), 7.43 (d, *J* = 7.90 Hz, 2H), 7.28 (d, *J* = 7.90 Hz, 2H), 7.22 (dd, *J* = 3.42 Hz, *J* = 0.5 Hz, 1H), 6.52 (dd, *J* = 2.69 Hz, *J* = 1.76 Hz, 1H), 5.44 (s, 2H), 5.34 (s, 2H). ^13^C NMR (CDCl_3_) δ = 181.17 (s), 158.46 (s), 158.31 (s), 146.63 (s), 146.51 (s), 145.35 (s), 144.46 (s), 135.94 (s), 135.34 (s), 128.89 (s). 127.62 (s), 126.77 (s), 121.46 (s), 118.31 (s), 117.30 (s), 111.90 (s), 105.22 (s), 65.99 (s), 44.498 (s). MS (ESI) m/z 540 [M+Na]; HRMS (ESI) m/z for C_21_H_13_Br_2_NO_5_ calculated 516.9160, found m/z: 539.9066.

4-((5,7-Dibromo-2,3-dioxoindolin-1-yl)methyl)benzaldehyde (**5**)

5,7-Dibromoisatin (I) (280 mg. 0.90 mmol) was dissolved in anhydrous DMF (6 mL) and stirred with K_2_CO_3_ (133 mg, 0.96 mmol) at room temperature for 1 h. After adding (248 mg, 1.25 mmol) of 4-(bromomethyl)benzaldehyde, the reaction mixture was agitated for 4 h. The reaction mixture was poured into cold water and extracted with ethyl acetate. The ethyl acetate layer was washed with water, brine and dried over MgSO_4_. The solvent was removed, and the crude product was purified by silica gel column chromatography (hexane: ethylacetate; 80:20) as eluent to obtain 5 as a yellow solid, yield: 68%; mp: 205–207 °C; ^1^H NMR (500 MHz, DMSO-*d*_6_): δ = 10.00 (s, 1H), 8.01 (d, *J* = 1.99 Hz, 1H), 7.89 (d, *J* = 7.18 Hz, 2H), 7.83 (d, *J* = 1.99 Hz, 1H), 7.65 (d, *J* = 8.18 Hz, 2H), 5.33 (s, 2H). ^13^C NMR (125 MHz, DMSO-*d*_6_): δ = 193.22, 180.92, 159.66, 146.52, 144.72, 143.61, 135.68, 130.17, 127.33, 126.75, 123.37, 116.20, 104.57, 44.81. MS (ESI) m/z 424 [M+H]; HRMS (ESI) m/z for C_16_H_9_Br_2_NO_3_ calculated 423.0600, found m/z: 423.9017.

4-((5-Methoxy-2,3-dioxoindolin-1-yl)methyl)benzyl carbamimidothioate hydrobromide (**6**)

The procedure used for 1 was repeated with 1-(4-(bromomethyl)benzyl)-5-methoxyindoline-2,3-dione (230 mg, 0.64 mmol) of (II), and (48 mg, 0.64 mmol) of thiourea. Compound **6** was obtained as an orange solid, yield: 73%); mp: 214–216 °C; ^1^H NMR (500 MHz, DMSO-*d*_6_): δ = 9.18 (br. s, 2H), 8.99 (br. s, 2H), 7.43 (d, *J* = 8.35 Hz, 2H), 7.40 (d, *J* = 8.35 Hz, 2H), 7.19~7.17 (m, 2H), 6.91 (dd, *J* = 9.25 Hz, *J* = 1.53 Hz, 1H), 4.89 (s, 2H), 4.40 (s, 2H), 3.76 (s, 3H). ^13^C NMR (125 MHz, DMSO-*d*_6_): δ =183.77, 169.39, 158.83, 156.30, 144.49, 135.98, 134.74, 129.74, 128.25, 124.16, 118.72, 112.52, 109.82, 56.39, 43.03, 34.33. MS (ESI) m/z 356 [M+H]; HRMS (ESI) m/z for C_18_H_17_N_3_O_3_S calculated 355.4120, found m/z: 356.1037.

4-((2,3-Dioxo-5-(trifluoromethyl)indolin-1-yl)methyl)benzyl allylcarbamimidothioate hydrobromide (**7**)

The procedure used for 1 was repeated with 1-(4-(bromomethyl)benzyl)-5-(trifluoromethyl)indoline-2,3-dione (260 mg, 0.65 mmol) of (II), and (76 mg, 0.65 mmol) of allylthiourea. The product **7** was an orange solid, yield: 69%; mp: 122–124 °C; ^1^H NMR (500 MHz, DMSO-*d*_6_): δ = 9.84 (br. s, 1H), 9.43 (br. s, 1H), 9.16 (br. s, 1H), 7.95 (d, *J* = 8.25 Hz, 1H), 7.89 (s, 1H), 7.47 (d, *J* =8.12 Hz, 2H), 7.39 (d, *J* = 8.12 Hz, 2H), 7.13 (d, *J* = 8.45 Hz, 1H), 5.78~5.70 (m, 1H), 5.13 (d, *J* = 10.38 Hz, 1H), 5.04 (d, *J* = 16.54 Hz, 1H), 4.98 (s, 2H), 4.53 (s, 2H), 3.93 (d, *J* = 4.75 Hz, 2H). ^13^C NMR (125 MHz, DMSO-*d*_6_): δ = 165.57, 158.99, 153.30, 135.45, 135.03, 134.84 (q, *J* = 3.62 Hz), 131.68, 129.72, 128.20, 124.36 (q, *J* = 271.59 Hz), 124.17 (q, *J* = 32.66 Hz), 123.26, 121.57, 121.52 (q, *J* = 3.70 Hz), 118.85, 117.64, 111.91, 65.39, 45.91, 43.25, 34.91. MS (ESI) m/z 434 [M+H]; HRMS (ESI) m/z for C_21_H_18_F_3_N_3_O_2_S calculated 433.4492, found m/z: 434.1117.

Methyl (*E*)-3-(4-((5-(trifluoromethyl)-1H-indazol-1-yl)methyl)phenyl)acrylate (**8**); Methyl (*E*)-3-(4-((5-(trifluoromethyl)-2H-indazol-2-yl)methyl)phenyl)acrylate (**9**)

Sodium hydride (60% in oil, 0.14 mg, 5.12 mmol) was added slowly to a solution of compound (III) (500 mg, 3.35 mmol) in anhydrous *N,N*-dimethylformamide (15 mL) at room temperature. The reaction mixture was stirred for 30 min at an ambient temperature of 60 °C. Methyl (2*E*)-3-[4-(bromomethyl)phenyl]-2-propenoate (1.71 g, 6.7 mmol) was then added, and the reaction mixture was stirred for an additional 4 h at 60 °C. The mixture was poured into ice-cold water, and the product was extracted by ethyl acetate, washed with water, and the isomers were purified by silica gel column chromatography eluting with 5% ethyl acetate in hexanes to provide products 8 and 9 as white solids. Compound **8**, yield 40%; mp: 125–127 °C; ^1^H NMR (500 MHz, DMSO-*d*_6_) δ: H_1_ = 8.33 (s, 1H), H_4_ = 7.81 (d, *J* = 8.95 Hz, 1H), H_5_ = 7.45 (m, 1H), H_7_ = 8.27 (s, 1H), H_10_ = 5.79 (s, 2H), H_12_ = 7.26 (d, *J* = 8.23 Hz, 2H), H_13_ = 7.61 (d, *J* = 8.23 Hz, 2H), H_15_ = 7.61 (d, *J* = 16.13 Hz, 1H), H_16_ = 6.60 (d, *J* = 16.13 Hz, 1H), H_18_ = 3.71 (s, 3H). ^13^C NMR (125 MHz, DMSO-*d*_6_) δ: C_17_ = 167.05, C_15_ = 144.41, C_9_ = 140.82, C_11_ = 139.9, C_1_ = 135.34, C_14_ = 133.94, C_13_ = 129.10, CF_3_ = 125.36 (q, *J* = 271.44 Hz), C_8_ = 123.42, C_7_ = 123.08 (q, *J* = 2.95 Hz), C_6_ = 122.01 (q, *J* = 31.54 Hz), C_5_ = 120.06 (q, *J* = 4.60 Hz), C_12_ = 128.30, C_16_ = 118.42, C_4_ = 111.56, C_10_ = 52.17, C_18_ = 51.93. MS (ESI) m/z 361 [M+H]; HRMS (ESI) m/z for C_19_H_15_F_3_N_2_O_2_ calculated 360.3362, found m/z: 361.1138.

Compound **9**, yield 26%; mp: 146–148 °C; ^1^H NMR (500 MHz, DMSO-*d*_6_) δ: H_1_ = 8.77(s, 1H), H_7_ = 8.26 (s, 1H), H_4_ = 7.81 (d, *J* = 8.91 Hz, 1H), H_13_ = 7.72 (d, *J* = 8.23 Hz, 2H), H_15_ = 7.64 (d, *J* = 16.10 Hz, 1H), H_5_ = 7.45 (dd, *J* = 8.91 Hz, *J* = 1.59 Hz, 1H), H_12_ = 7.37 (d, *J* = 8.23 Hz, 2H), H_16_ = 6.63 (d, *J* = 16.10 Hz, 1H), H_10_ = 5.76 (s, 2H), H_18_ = 3.72 (s, 3H). ^13^C NMR (125 MHz, DMSO-*d*_6_) δ: C_17_ = 167.06, C_9_ = 149.13, C_4_ = 144.37, C_11_ = 139.27, C_14_ = 134.23, C_13_ = 129.11, C_12_ = 128.77, C_1_ = 127.47, CF_3_ = 125.41 (q, *J* = 271.62 Hz), C_6_ = 122.20 (q, *J* = 31.37 Hz), C_5_ = 121.67 (q, *J* = 2.93 Hz), C_7_ = 120.68 (q, *J* = 5.05 Hz), C_8_ = 120.59, C_15_ = 118.99, C_16_ = 118.64, C_10_ = 56.65, C_18_ = 51.95. MS (ESI) m/z 361 [M+H]; HRMS (ESI) m/z for C_19_H_15_F_3_N_2_O_2_ calculated 360.3362, found m/z: 361.1146.

4-((5-(Trifluoromethyl)-1H-indazol-1-yl)methyl)benzyl allylcarbamimidothioate hydrobromide (**10**)

The procedure used for 1 was repeated with 1-(4-(bromomethyl)benzyl)-5-(trifluoromethyl)-1H-indazole (IV) (210 mg, 0.57 mmol), and (66 mg, 0.57 mmol) of allylthiourea. The product was a white solid, yield: 61%; mp: 104–106 °C; ^1^H NMR (500 MHz, DMSO-*d*_6_): δ = 9.80 (br. s, 1H), 9.33 (br. s, 2H), 8.31 (s, 1H), 8.27 (s, 1H), 7.97 (d, *J* = 8.96 Hz,1H), 7.68 (dd, *J* = 9.08 Hz, *J* = 1.29 Hz, 1H), 7.36 (d, *J* = 7.98 Hz, 2H), 7.25 (d, *J* = 7.98 Hz, 2H), 5.75 (s, 2H), 5.72–5.66 (m, 1H), 5.02 (d, *J* = 10.61 Hz, 1H), 4.96 (d, *J* = 17.02 Hz, 1H), 4.51 (s, 2H), 3.91 (d, *J* = 4.76 Hz, 2H). ^13^C NMR (125 MHz, DMSO-*d*_6_): δ = 165.46, 140.76, 137.40, 135.27, 135.20, 132.64, 129.69, 128.630, 125.36 (q, *J* = 271.27 Hz), 123.38, 123.02 (q, *J* = 3.18 Hz), 121.96 (q, *J* = 31.54 Hz), 120.05 (q, *J* = 4.51 Hz), 117.51, 111.59, 52.12, 45.89, 34.93. MS (ESI) m/z 405 [M+H]; HRMS (ESI) m/z for C_20_H_19_F_3_N_4_S calculated 404.4552, found m/z: 405.1335.

4-((5-(Trifluoromethyl)-2H-indazol-2-yl)methyl)benzyl allylcarbamimidothioate hydrobromide (**11**)

The procedure used for 1 was repeated with 2-(4-(bromomethyl)benzyl)-5-(trifluoromethyl)-2H-indazole (V) (160 mg, 0.43 mmol), and (50 mg, 0.43 mmol) of allylthiourea. The product was a white solid, yield: 46%; mp: 105–107 °C; ^1^H NMR (500 MHz, DMSO-*d*_6_): δ = 9.81 (br. s, 2H), 9.33 (br. s, 2H), 8.32 (s, 1H), 8.27 (s, 1H), 7.97 (d, *J* = 8.96 Hz, 1H), 7.68 (dd, *J* =9.08 Hz, *J* = 1.29 Hz, 1H), 7.36 (d, *J* = 7.98 Hz, 2H), 7.25 (d, *J* = 7.98 Hz, 2H), 5.75 (s, 2H), 5.72~5.66 (m, 1H), 5.02 (d, *J* = 10.61 Hz, 1H), 4.96 (d, *J* = 17.02, 1H), 4.51 (s, 2H), 3.91 (d, *J* = 4.76 Hz, 2H). ^13^C NMR (125 MHz, DMSO-*d*_6_): δ = 165.47, 149.07, 136.81, 135.58, 131.64, 129.70, 128.92, 127.39, 125.41 (q, *J* = 271.50 Hz), 122.16 (q, *J* = 31.19 Hz), 121.57 (q, *J* = 2.72 Hz), 120.67 (q, *J* = 4.90 Hz), 120.57, 118.98, 117.63, 56.61, 45.91, 34.94. MS (ESI) m/z 405 [M+H]; HRMS (ESI) m/z for C_20_H_19_F_3_N_4_S calculated 404.4552, found m/z: 405.1337.

4-((5-(Trifluoromethyl)-1H-indazol-1-yl)methyl)benzylcarbamimidothioate hydrobromide (**12**)

The procedure used for 1 was repeated with 1-(4-(bromomethyl)benzyl)-5-(trifluoromethyl)-1H-indazole (IV) (220 mg, 0.60 mmol), and (45 mg, 0.60 mmol) of thiourea. The product was a white solid, yield: 66%; mp: 183–185 °C; ^1^H NMR (500 MHz, DMSO-*d*_6_): δ = 9.18 (br. s, 2H), 8.98 (br. s, 2H), 8.33 (s, 1H), 8.27 (s, 1H), 7.97 (d, *J* = 8.94 Hz, 1H), 7.69 (dd, *J* = 8.94 Hz, *J* = 1.42 Hz, 1H), 7.37 (d, *J* = 8.04 Hz, 2H), 7.24 (d, *J* = 8.04 Hz, 2H), 5.75 (s, 2H), 4.46 (s, 2H). ^13^C NMR (125 MHz, DMSO-*d*_6_): δ = 169.35, 140.79, 137.43, 135.30, 134.92, 129.70, 128.29, 125.37 (q, *J* = 271.04 Hz), 123.36, 123.05 (q, *J* = 3.12 Hz), 121.96 (q, *J* = 31.58 Hz), 120.06 (q, *J* = 4.55 Hz), 111.58, 52.09, 34.31. MS (ESI) m/z 365 [M+H]; HRMS (ESI) m/z for C_17_H_15_F_3_N_4_S calculated 364.3902, found m/z: 365.1039.

4-((5-(Trifluoromethyl)-2H-indazol-2-yl)methyl)benzyl carbamimidothioate hydrobromide (**13**)

The procedure used for 1 was repeated with 2-(4-(bromomethyl)benzyl)-5-(trifluoromethyl)-2H-indazole (V) (230 mg, 0.62 mmol), and (47 mg, 0.62 mmol) of thiourea. The product was white solid, yield: 58%; mp: 181–183 °C; ^1^H NMR (500 MHz, DMSO-*d*_6_): δ = 9.19 (br. s, 2H), 8.98 (br. s, 2H), 8.78 (s, 1H), 8.26 (s, 1H), 7.81 (d, *J* = 9.17 Hz, 1H), 7.45 (dd, *J* = 9.17 Hz, *J* = 1.42 Hz, 1H), 7.42 (d, *J* = 8.14 Hz, 2H), 7.35 (d, *J* = 8.14 Hz, 2H), 5.73 (s, 2H), 4.49 (s, 2H). ^13^C NMR (125 MHz, DMSO-*d*_6_): δ = 169.34, 149.07, 136.83, 135.32, 129.72, 128.92, 127.43, 125.41 (q, *J* = 271.28 Hz), 122.16 (q, *J* = 31.35 Hz), 121.58 (q, *J* = 2.82 Hz), 120.68 (q, *J* = 4.96 Hz), 120.57, 118.99, 56.60, 34.31. MS (ESI) m/z 365 [M+H]; HRMS (ESI) m/z for C_17_H_15_F_3_N_4_S calculated 364.3902, found m/z: 365.1034.

4-((7-(Trifluoromethyl)-1H-indazol-1-yl)methyl)benzyl carbamimidothioate hydrobromide (**14**)

The procedure used for 1 was repeated with 1-(4-(bromomethyl)benzyl)-7-(trifluoromethyl)-1H-indazole (IV) (240 mg, 0.65 mmol), and (50 mg, 0.65 mmol) of thiourea. The product was a white solid, yield: 59%; mp: 206–208 °C; ^1^H NMR (500 MHz, DMSO-*d*_6_): δ = 9.16 (br. s, 2H), 8.97 (br. s, 2H), 8.23 (d, *J* = 8.07 Hz, 1H), 7.88 (d, *J* = 7.38 Hz, 1H), 7.37 (t, *J* = 7.67 Hz, 1H), 7.33 (d, *J* = 8.09 Hz, 2H), 6.94 (d, *J* = 8.09 Hz, 2H), 5.76 (s, 2H), 4.45 (s, 2H). ^13^C NMR (125 MHz, DMSO-*d*_6_): δ = 169.37, 138.09, 136.08, 134.95, 134.29, 129.53, 127.55, 127.25, 126.91, 126.30 (q, *J* = 6.40 Hz), 124.30 (q, *J* = 271.69 Hz), 120.65, 111.50 (q, *J* = 32.93 Hz), 53.99 (q, *J* = 4.12 Hz), 34.33. MS (ESI) m/z 365 [M+H]; HRMS (ESI) m/z for C_17_H_15_F_3_N_4_S calculated 364.3902, found m/z: 365.1051.

4-((7-(Trifluoromethyl)-2H-indazol-2-yl)methyl)benzyl carbamimidothioate hydrobromide (**15**)

The procedure used for 1 was repeated with 2-(4-(bromomethyl)benzyl)-7-(trifluoromethyl)-2H-indazole (V) (220 mg, 0.60 mmol), and (45 mg, 0.60 mmol) of thiourea. The product was a white solid, yield: 53%; mp: 195–197 °C; ^1^H NMR (500 MHz, DMSO-*d*_6_): δ = 9.19 (br. s, 2H), 8.99 (br. s, 2H), 8.74 (s, 1H), 8.07 (d, *J* = 8.53 Hz, 1H), 7.67 (d, *J* = 7.31 Hz, 1H), 7.43 (d, *J* = 8.14 Hz, 2H), 7.35 (d, *J* = 8.14 Hz, 2H), 7.19 (t, *J* = 7.77 Hz, 1H), 5.76 (s, 2H), 4.49 (s, 2H). ^13^C NMR (125 MHz, DMSO-*d*_6_): δ = 169.35, 143.60, 136.84, 135.31, 129.75, 128.76, 126.70, 126.24, 124.75 (q, *J* = 5.22 Hz), 124.57 (q, *J* = 272.86 Hz), 123.24, 120.37, 117.30 (q, *J* = 32.32 Hz), 56.54, 34.41. MS (ESI) m/z 365 [M+H]; HRMS (ESI) m/z for C_17_H_15_F_3_N_4_S calculated 364.3902, found m/z: 365.1052.

4-((3-Bromo-5-(trifluoromethyl)-1H-indazol-1-yl)methyl)benzyl carbamimidothioate hydrobromide (**16**)

The procedure used for 1 was repeated with 3-bromo-1-(4-(bromomethyl)benzyl)-5-(trifluoromethyl)-1H-indazole (IV) (260 mg, 0.58 mmol), and (44 mg, 0.58 mmol) of thiourea. The product was a white solid, yield: 53%; mp: 213–215 °C; ^1^H NMR (500 MHz, DMSO-*d*_6_): δ = 9.17 (br. s, 2H), 8.97 (br. s, 2H), 8.10 (d, *J* = 8.94 Hz, 1H), 7.99 (s, 1H), 7.82 (dd, *J* = 8.94 Hz, *J* = 1.28 Hz, 1H), 7.39 (d, *J* = 8.02 Hz, 2H), 7.28 (d, *J* = 8.02 Hz, 2H), 5.76 (s, 2H), 4.46 (s, 2H). ^13^C NMR (125 MHz, DMSO-*d*_6_): δ = 169.33, 142.14, 136.84, 135.17, 129.80, 128.48, 124.93 (q, *J* = 272.43 Hz), 124.62 (q, *J* = 2.96 Hz), 123.21 (q, *J* = 32.05 Hz), 122.76 (s), 121.77, 118.54 (q, *J* = 4.51 Hz), 112.63, 52.58, 34.29. MS (ESI) m/z 444 [M+H]; HRMS (ESI) m/z for C_17_H_15_BrF_3_N_4_S calculated 443.2862, found m/z: 444.0212.

4-((3-Bromo-7-(trifluoromethyl)-1H-indazol-1-yl)methyl)benzyl carbamimidothioate hydrobromide (**17**)

The procedure used for 1 was repeated with 3-bromo-1-(4-(bromomethyl)benzyl)-7-(trifluoromethyl)-1H-indazole (IV) (230 mg, 0.51 mmol), and (44 mg, 0.51 mmol) of thiourea. The product was a white solid, yield: 43%; mp: 235–237 °C; ^1^H NMR (500 MHz, DMSO-*d*_6_): δ = 9.17 (br. s, 2H), 8.97 (br. s, 2H), 8.03 (t, *J* = 7.44 Hz, 2H), 7.49 (t, *J* = 7.80 Hz, 1H), 7.36 (d, *J* = 7.93 Hz, 2H), 7.00 (d, *J* = 7.93 Hz, 2H), 5.73 (s, 2H), 4.46 (s, 2H). ^13^C NMR (125 MHz, DMSO-*d*_6_): δ = 169.36, 137.37, 136.33, 134.59, 129.65, 128.05 (q, *J* = 5.81 Hz), 127.05, 126.35, 126.26, 123.88 (q, *J* = 271.37 Hz), 123.08, 121.98, 112.12 (q, *J* = 32.87 Hz), 54.53 (q, *J* = 3.81 Hz), 34.32. MS (ESI) m/z 444 [M+H]; HRMS (ESI) m/z for C_17_H_15_BrF_3_N_4_S calculated 443.2862, found m/z: 444.0212.

4-((3-Bromo-5-methoxy-1H-indazol-1-yl)methyl)benzyl carbamimidothioate hydrobromide (**18**)

The procedure used for 1 was repeated with 3-bromo-1-(4-(bromomethyl)benzyl)-5-methoxy-1H-indazole (IV) (200 mg, 0.49 mmol), and (37 mg, 0.49 mmol) of thiourea. The product was a white solid, yield: 41%; mp: 171–173 °C; ^1^H NMR (500 MHz, DMSO-*d*_6_): δ = 9.16 (br. s, 2H), 8.96 (br. s, 2H), 7.73 (d, *J* = 9.26 Hz, 1H), 7.38 (d, *J* = 8.32 Hz, 2H), 7.24 (d, *J* = 8.32 Hz, 2H), 7.15 (dd, *J* = 9.26 Hz, *J* = 2.26 Hz, 1H), 6.92 (d, *J* = 2.26 Hz, 1H), 5.63 (s, 2H), 4.45 (s, 2H), 3.83 (s, 3H). ^13^C NMR (125 MHz, DMSO-*d*_6_): δ = 169.34, 155.57, 137.39, 136.86, 134.93, 129.71, 128.34, 123.77, 120.62, 118.96, 112.25, 98.76, 56.05, 52.40, 34.33. MS (ESI) m/z 406 [M+H]; HRMS (ESI) m/z for C_17_H_17_BrN_4_OS calculated 405.3140, found m/z: 406.0435.

(4-((3-Bromo-5-(trifluoromethyl)-1H-indazol-1-yl)methyl)phenyl)methanol (19)

Sodium hydride (60% in oil, 57 mg, 1.41 mmol) was added slowly to a solution of compound (III) (300 mg, 1.13 mmol) in anhydrous *N,N*-dimethylformamide (10 mL) at room temperature. The reaction mixture was stirred for 30 min at an ambient temperature to 60 °C. After adding the 4-(bromomethyl)benzyl)oxy)TBDMS (425 mg, 1.42 mmol), it was stirred for another 4 h at 60 °C. The product was extracted with ethyl acetate, washed with water, and purified by silica gel column chromatography using 10% ethyl acetate in hexanes to yield an intermediate viscous product, 3-bromo-5-(trifluoromethyl)-1-(4(2,3,3-trimethylbutan-2-yl)oxy) methyl)benzyl)-1H-indazole. The intermediate was stirred with tetrabutylammonium fluoride in THF for 1 h at RT. The reaction mixture was concentrated and the product was purified by silica gel column chromatography using 20% ethyl acetate in hexane, yield 43%; mp: 96–98 °C; ^1^H NMR (500 MHz, DMSO-*d*_6_): δ = 8.06 (d, *J* = 8.06 Hz, 1H), 7.97 (s, 1H), 7.80 (dd, *J* = 9.04 Hz, *J* = 1.47 Hz, 1H), 7.26 (q, *J* = 8.08 Hz, 4H), 5.71 (s, 2H), 5.16 (t, *J* = 5.64 Hz, 1H), 4.45 (d, *J* = 5.64 Hz, 2H). ^13^C NMR (125 MHz, DMSO-*d*_6_): δ = 142.79, 142.00, 135.24, 127.90, 127.23, 124.93 (q, *J* = 272.09 Hz), 124.50 (q, *J* = 3.01 Hz), 123.75 (q, *J* = 32.04 Hz), 121.51, 118.47 (q, *J* = 4.59 Hz), 112.64, 63.00, 52.89. MS (ESI) m/z 386 [M+H]; HRMS (ESI) m/z for C_16_H_12_BrF_3_N_2_O calculated 385.1842, found m/z: 386.0212.

4-((3-Bromo-5-(trifluoromethyl)-1H-indazol-1-yl)methyl)benzaldehyde (**20**)

Sodium hydride (60% in oil, 57 mg, 1.41 mmol) was added slowly to a solution of compound (III) (230 mg, 0.87 mmol) in anhydrous *N,N*-dimethylformamide (6 mL) at room temperature. The reaction mixture was stirred for 30 min at an ambient temperature to 60 °C. It was stirred for another 4 h at 60 °C after adding the 4-(bromomethyl)benzaldehyde (224 mg, 1.13 mmol). The mixture was poured into ice cold water, and the product was extracted by ethyl acetate, washed with water, and purified by silica gel column chromatography by eluting with 10% ethyl acetate in hexanes to obtain **20** as a white solid, yield 56%; mp: 108–110 °C; ^1^H NMR (500 MHz, DMSO-*d*_6_): δ = 9.97 (s, 1H), 8.08 (d, *J* = 9.00 Hz, 1H), 8.00 (s, 1H), 7.88 (d, *J* = 8.30 Hz, 2H), 7.83 (dd, *J* = 9.02 Hz, *J* = 1.26 Hz, 1H), 7.44 (d, *J* = 8.06 Hz); 2H), 5.88 (s, 2H). ^13^C NMR (125 MHz, DMSO-*d*_6_): δ = 193.14, 143.58, 142.32, 136.15, 130.42, 128.55, 124.90 (q, *J* = 272.10 Hz), 124.76 (q, *J* = 3.12 Hz), 123.34 (q, *J* = 32.20 Hz), 122.84, 122.09, 118.59 (q, *J* = 4.50 Hz), 112.57, 52.61. MS (ESI) m/z 384 [M+H]; HRMS (ESI) m/z for C_16_H_10_BrF_3_N_2_O calculated 383.1682, found m/z: 384.0061.

1-(4-((3-Bromo-5-(trifluoromethyl)-1H-indazol-1-yl)methyl)phenyl)-3-(dimethylamino)propan-1-one hydrochloride (**21**)

Sodium hydride (60% in oil, 52 mg, 1.32 mmol) was added slowly to a solution of compound (iii) (R_1_ = Br, R_2_ = CF_3_, R_3_ = H) (280 mg, 1.06 mmol) in anhydrous *N,N*-dimethylformamide (10 mL) at room temperature. The reaction mixture was stirred for 30 min at an ambient temperature of 60 °C. After adding the 1-(4-(bromomethyl)phenyl)ethan-1-one (282 mg, 1.33 mmol), it was stirred for another 4 h at 60 °C. The mixture was poured into ice-cold water, and the product was extracted by ethyl acetate, washed with water, concentrated, and purified by silica gel column chromatography by eluting with 20% ethyl acetate in hexanes to obtain an intermediate product 1-(4-(3-bromo-5-(trifluoromethyl)-1H-indazol-1-yl)methyl)phenyl)ethan-1-one (VI) as a white solid. The intermediate (200 mg, 0.50 mmol), dimethylamine hydrochloride, 55 mg (0.65 mmol), paraformaldehyde (40 mg, 1.3 mmol), and 20 mL of ethanol were heated to reflux with stirring. To this mixture was added 20 drops of conc. HCl and the reaction was heated at 80 °C for 18 h. The reaction mixture was cooled, and ether was added. The product was precipitated and filtered and washed with ether to provide **21** as a white solid, yield 62%; mp: 171–173 °C; ^1^H NMR (500 MHz, DMSO-*d*_6_): δ = 10.52 (s, 1H), 8.10 (d, *J* = 8.71 Hz, 1H), 8.00 (s, 1H), 7.98 (d, *J* = 8.20 Hz, 2H), 7.83 (dd, *J* = 9.04 Hz, J = 1.15 Hz, 1H), 7.42 (d, *J* = 8.20 Hz, 2H), 5.88 (s, 2H), 3.58 (t, *J* = 7.26 Hz, 2H), 3.38 (t, *J* = 7.26 Hz, 2H), 2.79 (s, 6H). ^13^C NMR (125 MHz, DMSO-*d*_6_): δ = 196.76, 142.66, 142.28, 135.87, 129.03, 128.30, 124.82 (q, *J* = 271.87 Hz), 124.72 (q, *J* = 2.80 Hz), 123.32 (q, *J* = 32.07 Hz), 122.84, 122.67, 118.57 (q, *J* = 4.52 Hz), 112.62, 52.58, 52.19, 42.72, 33.62. MS (ESI) m/z 455 [M+H]; HRMS (ESI) m/z for C_20_H_19_BrF_3_N_3_O calculated 454.2912, found m/z: 455.0811.

### 3.2. Biological Evaluation

#### 3.2.1. Cell Line and Culture Conditions

All of the cell lines used in this research were procured from the American Tissue Type Collection (ATCC, Manassas, VA, USA). The cell lines were maintained and cultured based on the suggestions provided by the ATCC (ThermoFisher Scientific, Waltham, MA USA). The cells were grown in the ATCC’s recommended growth media supplemented with 10% fetal bovine serum (FBS) (HyClone^®^, Logan, UT, USA). Each cell line was harvested using 0.25% trypsin-EDTA (Sigma–Aldrich) and counted using a hemocytometer for the experiments. Stock solutions of compounds were made up and delivered to the cells in DMSO.

#### 3.2.2. Molecular Docking Studies

To model the binding interactions of compound 3 with ALDH1A1, ALDH1A3, and ALDH3A1 proteins, the Autodock Vina was used to perform molecular docking [57]. The structures ALDH1A1 (PDB: 4X4L), ALDH1A3 (PDB: 7QK7), and ALDH3A1 (PDB: 3SZB) were retrieved on 25 May 2023 from the protein data bank (PDB) (https://www.rcsb.org//). Protein three-dimensional structures were prepared by removing water molecules from the structure coordinates files, adding hydrogen atoms and charges using MGL tools, and then converting it to PDBQT format as per the defined methodology. Prepared protein structures were used to generate scoring grids for further docking calculations. A grid box of size (40 × 40 × 40 Å) was centered on the corresponding active site position with 0.375 Å grid spacing. The grid dimensions and exhaustiveness values for ALDH1A1, ALDH1A3, and ALDH3A1 are given in Table 5. The ligand was prepared by setting the active torsions and detecting the root of the molecules to adjust them according to the defined pocket of the target proteins. The docking studies were carried out with rigid proteins, and all docking parameters were set to a default value. The schematic diagrams of the protein–ligand interactions were generated and analyzed using UCSF-Chimera [58].

#### 3.2.3. ALDEFLUOR Assay

Aldehyde dehydrogenase (ALDH) activity within human melanoma cells was assessed by utilizing the ALDEFLUOR Kit from STEMCELL Technologies. UACC 903 cells underwent separate 24 h treatments with 1 µM KS100, 1 µM Compound 3, and DMSO served as a control. Post-treatment, cells were washed with PBS, and the ALDEFLUOR assay was performed following the manufacturer’s protocol, employing DEAB as a negative control. ALDH percentages were determined using a BD LSRFortessa flow cytometer (San Jose, CA, USA).

#### 3.2.4. ALDH Isoform-Specific Enzyme Inhibition Assays

ALDH1A1, ALDH3A1, and ALDH1A3 enzyme inhibition assays were performed as reported previously [49]. Briefly, 500 ng/100 uL of ALDH1A1, ALDH3A1, and ALDH1A3 were treated with 500 nM concentration of the tested compounds and incubated for 15 min followed by the addition of reaction mixture (substrate + Substrate mixture). The plate was then incubated for another 2030 min until the conversion of NAD+ to NADH was visible as pink color production. The absorbance was measured at 450 nm. [Absorbance Control–Absorbance treated/Absorbance Control]100 was used to compute the percentage of inhibition.

#### 3.2.5. Cell Viability Assay

Five thousand cells/well were plated in 96-well plates in growth media and left for attachment for 24 h. The cells were then treated with the respective compounds using varying concentrations for 48 h. At the end of the treatment duration, a solution of MTS [3-(4,5-dimethylthiazol-2-yl)-5-(3-carboxymethoxyphenyl)-2-(4-sulfophenyl)-2H-tetrazolium] and an electron coupling reagent, PES (phenazine ethosulfate) (Promega, Madison, WI, USA) was added to the cells. Then, 15 μL of MTS reagent was added to each well, and the absorbance was assessed at 490 nm with a microplate reader (Dynex Technologies, MRX Revelation; Chantilly, VA, USA) after 1 h incubation with MTS. The data were recorded, and the IC_50_ value was calculated using non-linear curve regression analysis (Graph pad V8.0, CA, USA).

#### 3.2.6. Trypan Blue Dye Exclusion Assay

HCT-116 cells were seeded in a 6-well plate at a density of 5 x10^5^ cells/well and cultured for 24 h. The cells were then treated with 3 µM of compounds 3 and KS100 for 48 h. After 48 h of treatment, the cells were harvested and mixed with 1:4 volume of 0.4% trypan blue dye (Invitrogen, Waltham, MA, USA). Cells were loaded onto a hemocytometer and 4 fields per sample were counted using a bright field microscope. Live and healthy cells were unstained or excluded from the dye, whereas dead or membrane-compromised cells retained the dye and appeared blue.

#### 3.2.7. Apoptosis Assay

The apoptosis assays were carried out following the manufacturer’s instructions using the Muse Caspase-3/7 assay kit and Annexin V/7-AAD kit (Luminex Corp., TX, US). In a 6-well plate, the cells were seeded at a density of 1X105 cells/well. The cells were treated with 3 µM of the compounds following a 24 h incubation period. The cells were cultured for 48 h (Annexin V/7-AAD) as opposed to 24 h (Caspase-3/7 test). Cell Dissociation buffer, an enzyme-free solution, was used to harvest the cells at the end of the treatment period. The samples were then given the appropriate dye treatments following the manufacturer’s instructions.

#### 3.2.8. Chemifluorescence Assay for Reactive Oxygen Species (ROS) Generation

The Muse^®^ Oxidative Stress Kit (Luminex Corp., Austin, TX, USA) was used to assess the generation of ROS in the HCT-116 cells after the treatment with the compounds. In a 6-well plate, the cells were seeded at a density of 1 × 10^5^ cells/well. After 24 h incubation, the cells were treated with the respective compounds with or without 5 mM of N-acetylcysteine (NAC) in the serum-enriched medium for 24 h. At the end of the treatment duration, the cells were harvested, and 10 μL of cells in suspension was added into each tube. Furthermore, 190 μL of Muse Oxidative Stress Reagent working solution was added to each tube. The samples were mixed thoroughly by pipetting up and down or vortexing at medium speed for 3 to 5 s. The samples were incubated for 30 min at 37 °C and analyzed using Muse Cell Analyzer.

#### 3.2.9. Western Blot

Western blotting was performed as described previously [59]. The antibodies used were procured from Cell Signaling Technologies. Briefly, the HCT-116 cells were treated with 3 µM of compounds 3 and KS100 for 12 h. Subsequently, the cells were lysed, and the protein was loaded on a 4–15% SDS-PAGE gel (Bio-Rad, Philadelphia, PA, USA). The protein was transferred to a 0.45 µm pore membrane (Immobilon^®^-P PVDF Membrane, EMD Millipore, USA). The membranes were further treated with the respective primary and secondary antibodies. The Western blot images are representative of three independent experiments.

#### 3.2.10. Statistical Analysis

Statistical analysis was undertaken using one-way/two-way ANOVA in GraphPad PRISM Version 7.04 software. Dunnett’s post hoc analysis was performed to determine significant differences. A *p*-value of <0.05 was considered statistically significant.

## 4. Conclusion

This article describes the design and synthesis of new, multi-isoform, potentially more potent broad-spectrum ALDH inhibitors. Compounds were examined for in silico docking and antiproliferative properties in cell lines derived from multiple types of cancer, suggesting effectiveness and selectivity for killing cultured cancer cells. Compound **3** was more potent than **KS100**, a previously reported multi-isoform ALDH (ALDH1A1, ALDH1A3, and ALDH3A1) inhibitor. The inhibition of ALDH1A1 and 3A1 by compound **3** was slightly higher than that of **KS100**, while the inhibition of ALDH1A3 by compound **3** was three times greater than that of **KS100**. When compared to the isothiourea moiety in **KS100**, the nitro-furan moiety in compound **3** was the one that was responsible for the inhibition of ALDH1A3. Mechanistically, strong inhibition of multiple isoforms of ALDH led to increased ROS activity and apoptosis. Compound **3** (**KS124**) was identified as a potentially promising agent worthy of further investigation.

## Figures and Tables

**Table 1:** Docking scores of compounds (**1**–**21**). Docking scores were calculated for compounds against ALDH1A1, ALDH1A3, and ALDH3A1 using the Autodock Vina.

Docking Score (kcal/mol)			
Compound	ALDH1A1	ALDH1A3	ALDH3A1
**1**	−8.5	−8.3	−7.2
**2**	−8.8	−8.5	−8.3
**3**	−10.1	−9.6	−10.4
**4**	−9.2	−7.9	−7.9
**5**	−8.2	−7.4	−7.8
**6**	−8.7	−8.1	−7.7
**7**	−9.5	−8.2	−8.5
**8**	−9.2	−8.6	−8.7
**9**	−9.2	−8.4	−8.5
**10**	−9.2	−8.2	−8.6
**11**	−8.9	−8.2	−8.7
**12**	−8.9	−8.2	−9.0
**13**	−9.4	−7.2	−7.5
**14**	−8.0	−7.2	−8.3
**15**	−9.9	−8.5	−9.0
**16**	−9.2	−8.1	−8.1
**17**	−8.2	−7.9	−7.7
**18**	−9.5	−8.8	−8.2
**19**	−10.1	−9.1	−8.5
**20**	−10.0	−9.5	−9.1
**21**	−9.7	−8.6	−9.8
**KS100**	−7.9	−7.4	−8.3
**CM037**	−8.0	−7.6	−7.9

**Table 2:** Compounds **8** and **9** regio-isomer identification.

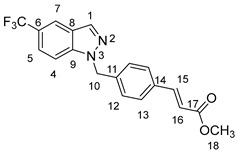 **Compound 8**	^1^H NMR (500 MHz, DMSO-*d*_6_) δ: H_1_ = 8.33 (s, 1H), H_4_ = 7.81 (d, *J* = 8.95 Hz, 1H), H_5_ = 7.45 (m, 1H), H_7_ = 8.27 (s, 1H), H_10_ = 5.79 (s, 2H), H_12_ = 7.26 (d, *J* = 8.23 Hz, 2H), H_13_ = 7.61 (d, *J* = 8.23 Hz, 2H), H_15_ = 7.61 (d, *J* = 16.13 Hz, 1H), H_16_ = 6.60 (d, *J* = 16.13 Hz, 1H), H_18_ = 3.71 (s, 3H).
	^13^C NMR (125 MHz, DMSO-*d*_6_) δ: C_17_ = 167.05, C_15_ = 144.41, C_9_ = 140.82, C_11_ = 139.9, C_1_ = 135.34, C_14_ = 133.94, C_13_ = 129.10, CF_3_ = 125.36 (q, *J* = 271.44 Hz), C_8_ = 123.42, C_7_ = 123.08 (q, *J* = 2.95 Hz), C_6_ = 122.01 (q, *J* = 31.54 Hz), C_5_ = 120.06 (q, *J* = 4.60 Hz), C_12_ = 128.30, C_16_ = 118.42, C_4_ = 111.56, C_10_ = 52.17, C_18_ = 51.93
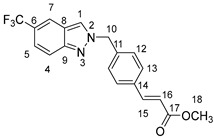 **Compound 9**	^1^H NMR (500 MHz, DMSO-*d*_6_) δ: H_1_ = 8.77 (s, 1H), H_7_ = 8.26 (s, 1H), H_4_ = 7.81 (d, *J* = 8.91 Hz, 1H), H_13_ = 7.72 (d, *J* = 8.23 Hz, 2H), H_15_ = 7.64 (d, *J* = 16.10 Hz, 1H), H_5_ = 7.45 (dd, *J* = 8.91 Hz, *J* = 1.59 Hz, 1H), H_12_ = 7.37 (d, *J* = 8.23 Hz, 2H), H_16_ = 6.63 (d, *J* = 16.10 Hz, 1H), H_10_ = 5.76 (s, 2H), H_18_ = 3.72 (s, 3H).
	^13^C NMR (125 MHz, DMSO-*d*_6_) δ: C_17_ = 167.06, C_9_ = 149.13, C_4_ = 144.37, C_11_ = 139.27, C_14_ = 134.23, C_13_ = 129.11, C_12_ = 128.77, C_1_ = 127.47, CF_3_ = 125.41 (q, *J* = 271.62 Hz), C_6_ = 122.20 (q, *J* = 31.37 Hz), C_5_ = 121.67 (q, *J* = 2.93 Hz), C_7_ = 120.68 (q, *J* = 5.05 Hz), C_8_ = 120.59, C_15_ = 118.99, C_16_ = 118.64, C_10_ = 56.65, C_18_ = 51.95

**Table 3:** In vitro antiproliferative activity of compounds against HCT-116, SKOV-3, MIA PaCa-2, HEK-293, and FHC cell lines.

Compound No.	Structure	^a^ IC_50_ (µM)				
		^b^ SKOV-3	^c^ MiaPaCa-2	^d^ HCT-116	^e^ HEK-293	^f^ FHC
**1**	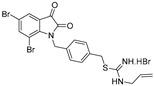	4.8 ± 0.2	2.4 ± 1.1	4.7 ± 1.1	4.1 ± 0.8	2.9 ± 0.3
**2**	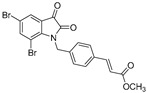	21.8 ± 2.4	22.3 ± 5.1	19.6 ± 3.3	24.1 ± 6.8	22.9 ± 3.3
**3** **(KS124)**	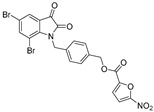	2.2 ± 0.2	3.8 ± 0.4	2.1 ± 0.1	8.7 ± 1.1	7.1 ± 0.8
**4**	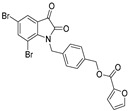	7.2 ± 1.3	7.1 ± 0.8	6.1 ± 1.2	5.3 ± 1.4	6.7 ± 1.9
**5**	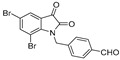	8.2 ± 1.1	5.4 ± 0.9	8.8 ± 2.3	7.5 ± 1.8	12.9 ± 3.3
**6**	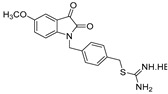	45.0 ± 2.5	>50	>50	>50	>50
**7**	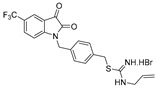	9.1 ± 1.3	10.3 ± 2.4	12.0 ± 3.1	9.7 ± 1.1	8.1 ± 0.8
**8**	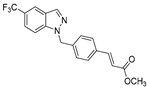	>50	>50	>50	>50	>50
**9**	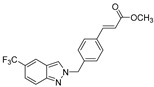	>50	>50	>50	>50	>50
**10**	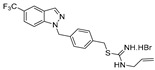	>50	48.4 ± 3.2	>50	>50	>50
**11**	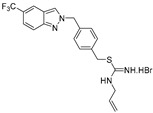	>50	>50	>50	>50	>50
**12**	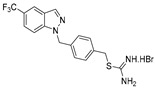	8.2 ± 2.7	4.7 ± 0.4	5.5 ± 0.1	4.7 ± 1.1	3.1 ± 0.8
**13**	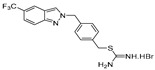	22.7 ± 2.3	33.2 ± 4.4	18.2 ± 3.1	18.9 ± 5.1	14.4 ± 2.3
**14**	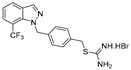	16.8 ± 3.5	12.3 ± 2.7	14.2 ± 2.3	17.7 ± 3.7	29.3 ± 4.9
**15**	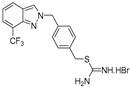	>50	>50	>50	>50	>50
**16**	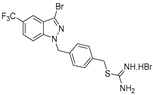	4.0 ± 1.3	6.8 ± 1.4	4.9 ± 1.1	5.7 ± 1.3	7.1 ± 1.1
**17**	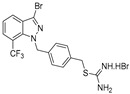	>50	>50	>50	>50	>50
**18**	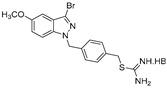	42.2 ± 5.8	44.6 ± 3.7	39.2 ± 3.9	>50	>50
**19**	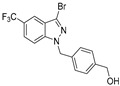	>50	>50	>50	>50	>50
**20**	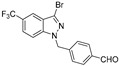	>50	>50	>50	>50	>50
**21**	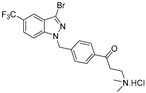	>50	>50	>50	>50	>50
**KS100**	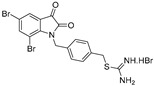	3.8 ± 0.8	2.8 ± 0.2	4.3 ± 0.7	4.3 ± 0.1	4.9 ± 0.8

^a^ IC_50_ = Compound concentration required to inhibit cell proliferation by 50%. Data are expressed as the mean ± SD from the dose-response curve of at least three independent experiments. ^b^ Ovarian cancer cells. ^c^ Pancreatic cancer cells. ^d^ Colon cancer cells. ^e^ Human embryonic kidney cells. ^f^ Normal fetal human cells.

**Table 4:** ALDH1A1, ALDH3A1, and ALDH1A3 enzymes inhibition study. Selected compounds were evaluated for ALDH1A1, ALDH3A1, and ALDH1A3 inhibitory activity. The % inhibition was calculated for each compound and compared to the DMSO control. ND: Not Determined.

Compounds	% Enzyme Inhibition at 500 nM		
	Acetaldehyde (Substrate)		4-Nitrobenzaldeyde (Substrate)
	ALDH1A1	ALDH3A1	ALDH1A3
**Control**	0 ± 0.02	0.05 ± 0.05	0 ± 0.01
**1**	35.37 ± 0.01	30.59 ± 0.02	ND
**3** **(KS124)**	51.32 ± 0.01	51.87 ± 0.05	36.65 ± 0.02
**4**	28.26 ± 0.01	22.82 ± 0.05	35.14 ± 0.07
**5**	24.82 ± 0.01	39.58 ± 0.05	ND
**12**	10.01 ± 0.01	−14.03 ± 0.06	ND
**16**	9.46 ± 0.01	−9.33 ± 0.04	ND
**17**	10.99 ± 0.0015	−25.33 ± 0.05	ND
**KS100**	45.93 ± 0.01	37.04 ± 0.09	13.83 ± 0.01
673A-IC_50_ [53]	246 nM	ND	348 nM
CB7-IC_50_ [33]	ND	200 nM	ND

**Table 5:** Autodock vina configuration parameter.

Configuration parameter			
Config	ALDH1A1 (4X4L)	ALDH1A3 (7QK7)	ALDH3A1 (3SZB)
center_x	−25.6	22.0	7.9
center_y	38.1	11.5	17.7
center_z	30.2	23.9	66.7
size_x	40	40	40
size_y	40	40	40
size_z	40	40	40
exhaustiveness	32	32	32

## Data Availability

All data are contained within the article and Supplementary Materials.

## Supplementary Materials

The following supporting information can be downloaded at: https://www.mdpi.com/article/10.3390/molecules29133114/s1. Copies of the ^1^H NMR and ^13^C NMR spectra for new compounds and cytotoxicity data

## Abbreviations

ALDHs: aldehyde dehydrogenases; 4-HNE, 4-hydroxy-2-nonenal; MDA, malondialdehyde; ROS, reactive oxygen species.

## References

[1] Mizumoto A., Ohashi S., Hirohashi K., Amanuma Y., Matsuda T., Muto M. (2017). Molecular Mechanisms of Acetaldehyde-Mediated Carcinogenesis in Squamous Epithelium. Int. J. Mol. Sci..

[2] Vasiliou V., Pappa A., Petersen D.R. (2000). Role of aldehyde dehydrogenases in endogenous and xenobiotic metabolism. Chem. Biol. Interact..

[3] O’Brien P.J., Siraki A.G., Shangari N. (2005). Aldehyde sources, metabolism, molecular toxicity mechanisms, and possible effects on human health. Crit. Rev. Toxicol..

[4] Esterbauer H., Schaur R.J., Zollner H. (1991). Chemistry and biochemistry of 4-hydroxynonenal, malonaldehyde and related aldehydes. Free Radic. Biol. Med..

[5] Nadkarni D.V., Sayre L.M. (1995). Structural definition of early lysine and histidine adduction chemistry of 4-hydroxynonenal. Chem. Res. Toxicol..

[6] Brooks P.J., Theruvathu J.A. (2005). DNA adducts from acetaldehyde: Implications for alcohol-related carcinogenesis. Alcohol.

[7] LoPachin R.M., Gavin T. (2014). Molecular mechanisms of aldehyde toxicity: A chemical perspective. Chem. Res. Toxicol..

[8] Allison S.E., Chen Y., Petrovic N., Zhang J., Bourget K., Mackenzie P.I., Murray M. (2017). Activation of ALDH1A1 in MDA-MB-468 breast cancer cells that over-express CYP2J2 protects against paclitaxel-dependent cell death mediated by reactive oxygen species. Biochem. Pharmacol..

[9] Zanoni M., Bravaccini S., Fabbri F., Arienti C. (2022). Emerging Roles of Aldehyde Dehydrogenase Isoforms in Anti-cancer Therapy Resistance. Front. Med..

[10] Ajani J.A., Wang X., Song S., Suzuki A., Taketa T., Sudo K., Wadhwa R., Hofstetter W.L., Komaki R., Maru D.M. (2014). ALDH-1 expression levels predict response or resistance to preoperative chemoradiation in resectable esophageal cancer patients. Mol. Oncol..

[11] Huang C.P., Tsai M.F., Chang T.H., Tang W.C., Chen S.Y., Lai H.H., Lin T.Y., Yang J.C., Yang P.C., Shih J.Y. (2013). ALDH-positive lung cancer stem cells confer resistance to epidermal growth factor receptor tyrosine kinase inhibitors. Cancer Lett..

[12] Charafe-Jauffret E., Ginestier C., Iovino F., Tarpin C., Diebel M., Esterni B., Houvenaeghel G., Extra J.M., Bertucci F., Jacquemier J. (2010). Aldehyde dehydrogenase 1-positive cancer stem cells mediate metastasis and poor clinical outcome in inflammatory breast cancer. Clin. Cancer Res..

[13] Marcato P., Dean C.A., Giacomantonio C.A., Lee P.W. (2011). Aldehyde dehydrogenase: Its role as a cancer stem cell marker comes down to the specific isoform. Cell Cycle.

[14] Durinikova E., Kozovska Z., Poturnajova M., Plava J., Cierna Z., Babelova A., Bohovic R., Schmidtova S., Tomas M., Kucerova L. (2018). ALDH1A3 upregulation and spontaneous metastasis formation is associated with acquired chemoresistance in colorectal cancer cells. BMC Cancer.

[15] Chen X., Li Q., Liu X., Liu C., Liu R., Rycaj K., Zhang D., Liu B., Jeter C., Calhoun-Davis T. (2016). Defining a Population of Stem-like Human Prostate Cancer Cells That Can Generate and Propagate Castration-Resistant Prostate Cancer. Clin. Cancer Res..

[16] Lin L., Jou D., Wang Y., Ma H., Liu T., Fuchs J., Li P.K., Lu J., Li C., Lin J. (2016). STAT3 as a potential therapeutic target in ALDH+ and CD44+/CD24+ stem cell-like pancreatic cancer cells. Int. J. Oncol..

[17] Sarvi S., Crispin R., Lu Y., Zeng L., Hurley T.D., Houston D.R., von Kriegsheim A., Chen C.H., Mochly-Rosen D., Ranzani M. (2018). ALDH1 Bio-activates Nifuroxazide to Eradicate ALDH(High) Melanoma-Initiating Cells. Cell Chem. Biol..

[18] Flahaut M., Jauquier N., Chevalier N., Nardou K., Balmas Bourloud K., Joseph J.M., Barras D., Widmann C., Gross N., Renella R. (2016). Aldehyde dehydrogenase activity plays a Key role in the aggressive phenotype of neuroblastoma. BMC Cancer.

[19] Xia J., Li S., Liu S., Zhang L. (2023). Aldehyde dehydrogenase in solid tumors and other diseases: Potential biomarkers and therapeutic targets. MedComm.

[20] Muzio G., Maggiora M., Paiuzzi E., Oraldi M., Canuto R.A. (2012). Aldehyde dehydrogenases and cell proliferation. Free Radic. Biol. Med..

[21] Vlashi E., Pajonk F. (2015). Cancer stem cells, cancer cell plasticity and radiation therapy. Semin. Cancer Biol..

[22] Bazewicz C.G., Dinavahi S.S., Schell T.D., Robertson G.P. (2019). Aldehyde dehydrogenase in regulatory T-cell development, immunity and cancer. Immunology.

[23] Rodriguez-Zavala J.S., Calleja L.F., Moreno-Sanchez R., Yoval-Sanchez B. (2019). Role of Aldehyde Dehydrogenases in Physiopathological Processes. Chem. Res. Toxicol..

[24] Oh T., Kwon M., Yu J.S., Jang M., Kim G.H., Kim K.H., Ko S.K., Ahn J.S. (2020). Ent-Peniciherqueinone Suppresses Acetaldehyde-Induced Cytotoxicity and Oxidative Stress by Inducing ALDH and Suppressing MAPK Signaling. Pharmaceutics.

[25] McLean M.E., MacLean M.R., Cahill H.F., Arun R.P., Walker O.L., Wasson M.D., Fernando W., Venkatesh J., Marcato P. (2023). The Expanding Role of Cancer Stem Cell Marker ALDH1A3 in Cancer and Beyond. Cancers.

[26] Koppaka V., Thompson D.C., Chen Y., Ellermann M., Nicolaou K.C., Juvonen R.O., Petersen D., Deitrich R.A., Hurley T.D., Vasiliou V. (2012). Aldehyde dehydrogenase inhibitors: A comprehensive review of the pharmacology, mechanism of action, substrate specificity, and clinical application. Pharmacol. Rev..

[27] Kimble-Hill A.C., Parajuli B., Chen C.H., Mochly-Rosen D., Hurley T.D. (2014). Development of selective inhibitors for aldehyde dehydrogenases based on substituted indole-2,3-diones. J. Med. Chem..

[28] Morgan C.A., Hurley T.D. (2015). Characterization of two distinct structural classes of selective aldehyde dehydrogenase 1A1 inhibitors. J. Med. Chem..

[29] Yang S.M., Yasgar A., Miller B., Lal-Nag M., Brimacombe K., Hu X., Sun H., Wang A., Xu X., Nguyen K. (2015). Discovery of NCT-501, a Potent and Selective Theophylline-Based Inhibitor of Aldehyde Dehydrogenase 1A1 (ALDH1A1). J. Med. Chem..

[30] Yang S.M., Martinez N.J., Yasgar A., Danchik C., Johansson C., Wang Y., Baljinnyam B., Wang A.Q., Xu X., Shah P. (2018). Discovery of Orally Bioavailable, Quinoline-Based Aldehyde Dehydrogenase 1A1 (ALDH1A1) Inhibitors with Potent Cellular Activity. J. Med. Chem..

[31] Kulsum S., Sudheendra H.V., Pandian R., Ravindra D.R., Siddappa G., Chevour P., Ramachandran B., Sagar M., Jayaprakash A., Mehta A. (2017). Cancer stem cell mediated acquired chemoresistance in head and neck cancer can be abrogated by aldehyde dehydrogenase 1 A1 inhibition. Mol. Carcinog..

[32] Huddle B.C., Grimley E., Buchman C.D., Chtcherbinine M., Debnath B., Mehta P., Yang K., Morgan C.A., Li S., Felton J. (2018). Structure-Based Optimization of a Novel Class of Aldehyde Dehydrogenase 1A (ALDH1A) Subfamily-Selective Inhibitors as Potential Adjuncts to Ovarian Cancer Chemotherapy. J. Med. Chem..

[33] Parajuli B., Fishel M.L., Hurley T.D. (2014). Selective ALDH3A1 inhibition by benzimidazole analogues increase mafosfamide sensitivity in cancer cells. J. Med. Chem..

[34] Li B., Yang K., Liang D., Jiang C., Ma Z. (2021). Discovery and development of selective aldehyde dehydrogenase 1A1 (ALDH1A1) inhibitors. Eur. J. Med. Chem..

[35] Quattrini L., Gelardi E.L.M., Petrarolo G., Colombo G., Ferraris D.M., Picarazzi F., Rizzi M., Garavaglia S., La Motta C. (2020). Progress in the Field of Aldehyde Dehydrogenase Inhibitors: Novel Imidazo [1,2-a]pyridines against the 1A Family. ACS Med. Chem. Lett..

[36] Quattrini L., Gelardi E.L.M., Coviello V., Sartini S., Ferraris D.M., Mori M., Nakano I., Garavaglia S., La Motta C. (2020). Imidazo [1,2-a]pyridine Derivatives as Aldehyde Dehydrogenase Inhibitors: Novel Chemotypes to Target Glioblastoma Stem Cells. J. Med. Chem..

[37] Gelardi E.L.M., Colombo G., Picarazzi F., Ferraris D.M., Mangione A., Petrarolo G., Aronica E., Rizzi M., Mori M., La Motta C. (2021). A Selective Competitive Inhibitor of Aldehyde Dehydrogenase 1A3 Hinders Cancer Cell Growth, Invasiveness and Stemness In Vitro. Cancers.

[38] Matsunaga N., Ogino T., Hara Y., Tanaka T., Koyanagi S., Ohdo S. (2018). Optimized Dosing Schedule Based on Circadian Dynamics of Mouse Breast Cancer Stem Cells Improves the Antitumor Effects of Aldehyde Dehydrogenase Inhibitor. Cancer Res..

[39] Morgan C.A., Parajuli B., Buchman C.D., Dria K., Hurley T.D. (2015). N,N-diethylaminobenzaldehyde (DEAB) as a substrate and mechanism-based inhibitor for human ALDH isoenzymes. Chem. Biol. Interact..

[40] Fournet G., Martin G., Quash G. (2013). alpha,beta-Acetylenic amino thiolester inhibitors of aldehyde dehydrogenases 1&3: Suppressors of apoptogenic aldehyde oxidation and activators of apoptosis. Curr. Med. Chem..

[41] Perez-Alea M., McGrail K., Sanchez-Redondo S., Ferrer B., Fournet G., Cortes J., Munoz E., Hernandez-Losa J., Tenbaum S., Martin G. (2017). ALDH1A3 is epigenetically regulated during melanocyte transformation and is a target for melanoma treatment. Oncogene.

[42] Venton G., Perez-Alea M., Baier C., Fournet G., Quash G., Labiad Y., Martin G., Sanderson F., Poullin P., Suchon P. (2016). Aldehyde dehydrogenases inhibition eradicates leukemia stem cells while sparing normal progenitors. Blood Cancer J..

[43] Thomas M.L., de Antueno R., Coyle K.M., Sultan M., Cruickshank B.M., Giacomantonio M.A., Giacomantonio C.A., Duncan R., Marcato P. (2016). Citral reduces breast tumor growth by inhibiting the cancer stem cell marker ALDH1A3. Mol. Oncol..

[44] Khanna M., Chen C.H., Kimble-Hill A., Parajuli B., Perez-Miller S., Baskaran S., Kim J., Dria K., Vasiliou V., Mochly-Rosen D. (2011). Discovery of a novel class of covalent inhibitor for aldehyde dehydrogenases. J. Biol. Chem..

[45] Kim J., Shin J.H., Chen C.H., Cruz L., Farnebo L., Yang J., Borges P., Kang G., Mochly-Rosen D., Sunwoo J.B. (2017). Targeting aldehyde dehydrogenase activity in head and neck squamous cell carcinoma with a novel small molecule inhibitor. Oncotarget.

[46] Okazaki S., Shintani S., Hirata Y., Suina K., Semba T., Yamasaki J., Umene K., Ishikawa M., Saya H., Nagano O. (2018). Synthetic lethality of the ALDH3A1 inhibitor dyclonine and xCT inhibitors in glutathione deficiency-resistant cancer cells. Oncotarget.

[47] Zeng S., Kapur A., Patankar M.S., Xiong M.P. (2015). Formulation, Characterization, and Antitumor Properties of Trans- and Cis-Citral in the 4T1 Breast Cancer Xenograft Mouse Model. Pharm. Res..

[48] Dinavahi S.S., Gowda R., Bazewicz C.G., Battu M.B., Lin J.M., Chitren R.J., Pandey M.K., Amin S., Robertson G.P., Gowda K. (2020). Design, synthesis characterization and biological evaluation of novel multi-isoform ALDH inhibitors as potential anticancer agents. Eur. J. Med. Chem..

[49] Dinavahi S.S., Gowda R., Gowda K., Bazewicz C.G., Chirasani V.R., Battu M.B., Berg A., Dokholyan N.V., Amin S., Robertson G.P. (2020). Development of a Novel Multi-Isoform ALDH Inhibitor Effective as an Antimelanoma Agent. Mol. Cancer Ther..

[50] Jackson B., Brocker C., Thompson D.C., Black W., Vasiliou K., Nebert D.W., Vasiliou V. (2011). Update on the aldehyde dehydrogenase gene (ALDH) superfamily. Hum. Genom..

[51] Januchowski R., Wojtowicz K., Zabel M. (2013). The role of aldehyde dehydrogenase (ALDH) in cancer drug resistance. Biomed. Pharmacother..

[52] Dinavahi S.S., Bazewicz C.G., Gowda R., Robertson G.P. (2019). Aldehyde Dehydrogenase Inhibitors for Cancer Therapeutics. Trends Pharmacol. Sci..

[53] Chefetz I., Grimley E., Yang K., Hong L., Vinogradova E.V., Suciu R., Kovalenko I., Karnak D., Morgan C.A., Chtcherbinine M. (2019). A Pan-ALDH1A Inhibitor Induces Necroptosis in Ovarian Cancer Stem-like Cells. Cell Rep..

[54] Van Engeland M., Nieland L.J., Ramaekers F.C., Schutte B., Reutelingsperger C.P. (1998). Annexin V-affinity assay: A review on an apoptosis detection system based on phosphatidylserine exposure. Cytom. J. Int. Soc. Anal. Cytol..

[55] Ozben T. (2007). Oxidative stress and apoptosis: Impact on cancer therapy. J. Pharm. Sci..

[56] Zhitkovich A. (2019). N-Acetylcysteine: Antioxidant, Aldehyde Scavenger, and More. Chem. Res. Toxicol..

[57] Trott O., Olson A.J. (2010). AutoDock Vina: Improving the speed and accuracy of docking with a new scoring function, efficient optimization, and multithreading. J. Comput. Chem..

[58] Pettersen E.F., Goddard T.D., Huang C.C., Couch G.S., Greenblatt D.M., Meng E.C., Ferrin T.E. (2004). UCSF Chimera--a visualization system for exploratory research and analysis. J. Comput. Chem..

[59] Nikhil K., Raza A., Haymour H.S., Flueckiger B.V., Chu J., Shah K. (2020). Aurora Kinase A-YBX1 Synergy Fuels Aggressive Oncogenic Phenotypes and Chemoresistance in Castration-Resistant Prostate Cancer. Cancers.

