# Supplementary material for: Identification of Novel Isatin Derivative Bearing a Nitrofuran Moiety as Potent Multi-Isoform Aldehyde Dehydrogenase Inhibitor
Source: Molecules. 2024 Jun 29;29(13):3114. doi: 10.3390/molecules29133114 (PMC11243058; doi:10.3390/molecules29133114)
Supplement: Supplementary file 1 [file molecules-29-03114-s001.zip › molecules-3050990-supplementary.pdf]

Supplementary Data

Identification of Novel Isatin Derivative Bearing a Nitrofuran Moiety as Potent Multi-Isoform Aldehyde Dehydrogenase Inhibitor

Krishne Gowda [1,*], Asif Raza [1], Venugopal Vangala [1], Nazir Ahmad Lone [1], Jyh Ming Lin [2], Jaikee Kumar Singh [3], Sandeep Kumar Srivastava [3], Todd D. Schell [4], Gavin P. Robertson [1,5], Shantu Amin [1] and Arun K. Sharma [1,*]

[1] Department of Pharmacology, Penn State Cancer Institute, Penn State College of Medicine, Hershey, PA 17033, USA
[2] Department of Biochemistry and Molecular Biology, Penn State Cancer Institute, Penn State College of Medicine Hershey, Hershey, PA 17033, USA
[3] Department of Biosciences, Manipal University Jaipur, Jaipur 303007, India; sandeepkumar.srivastava@jaipur.manipal.edu (S.K.S.)
[4] Department of Microbiology and Immunology, Penn State Cancer Institute, Penn State College of Medicine Hershey, Hershey, PA 17033, USA
[5] Departments of Pathology, Dermatology, Surgery, Melanoma Skin Cancer Center, Penn State Cancer Institute, Penn State College of Medicine Hershey, Hershey, PA 17033, USA
* Correspondence: kgowda@pennstatehealth.psu.edu (K.G.); asharma1@pennstatehealth.psu.edu (A.K.S.)

S. No.	Description	Page No.
1	Cellular IC_{50}-Dose response-ovary, pancreatic, and colon cancer cell lines	S2
2	Cellular IC_{50}-Dose response-normal cell lines	S3
3	HMBC Spectrum for compounds **8** and **9**	S4-5
4	^{1}H and ^{13}C - NMR spectra for the compounds **(1-21)**	S6-S26

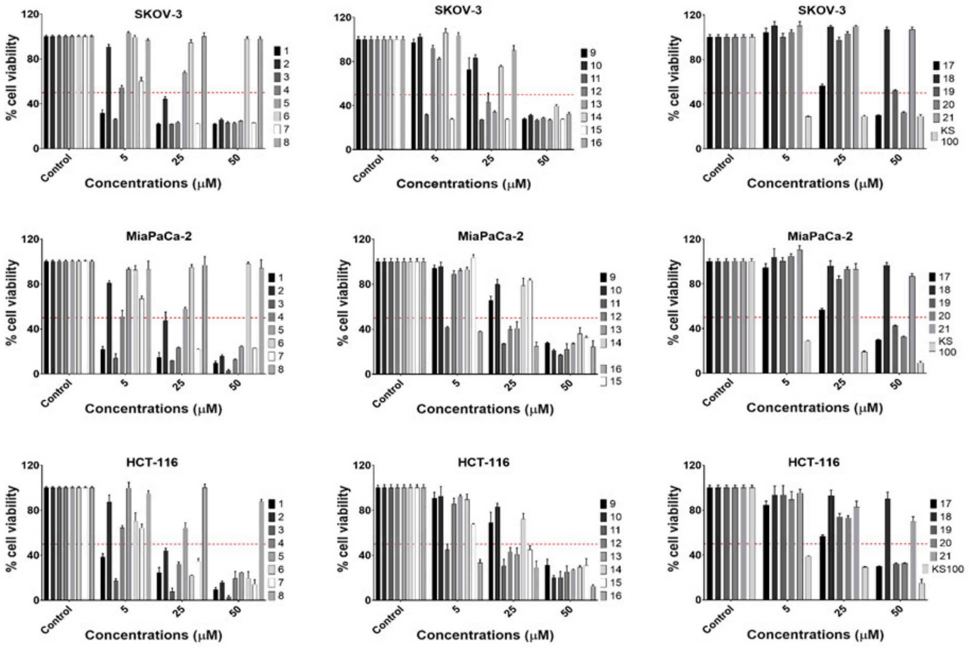

Figure S1. Cellular IC_{50} dose response for ovary, pancreatic, and colon cancer cell lines

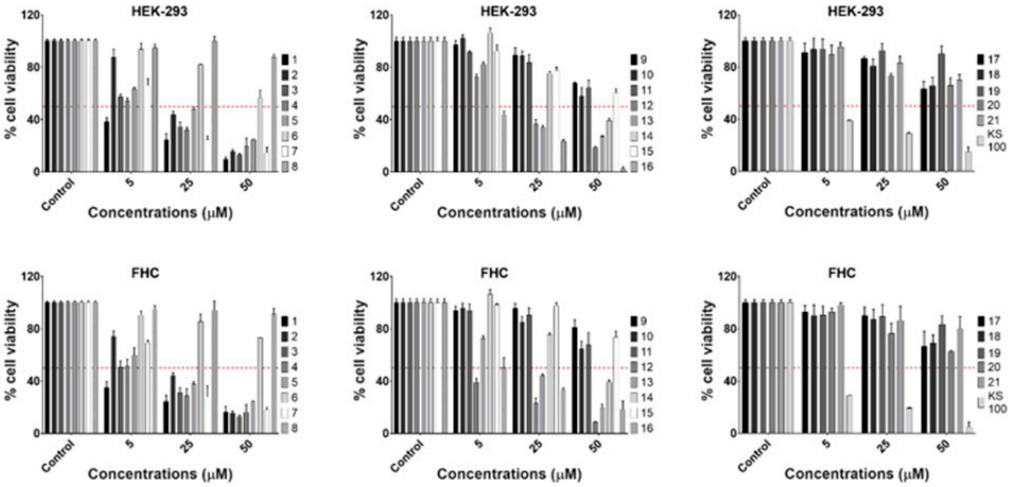

Figure S2. Cellular IC_{50} dose response for normal cell lines

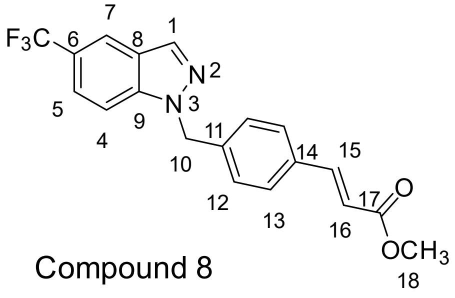

Compound 8

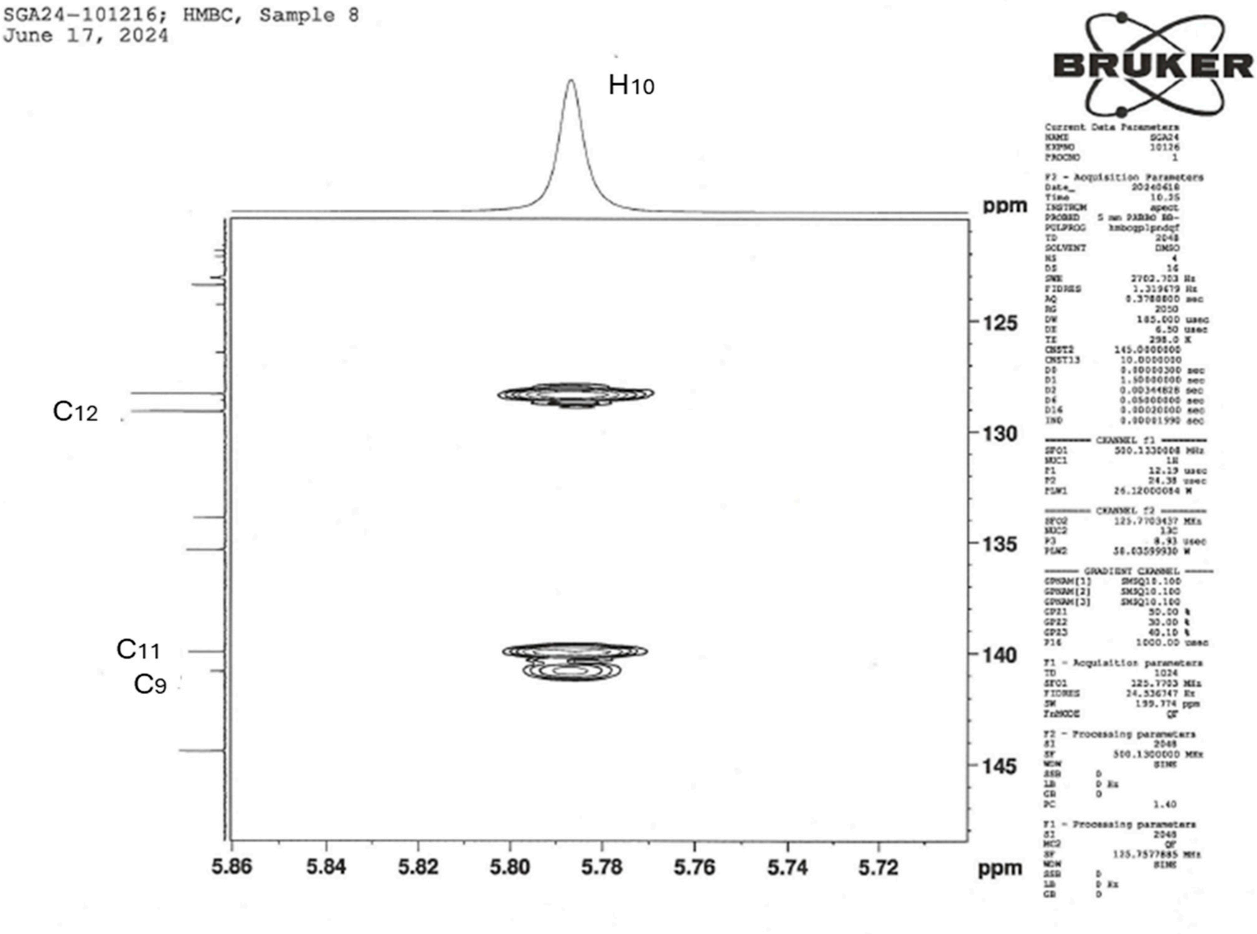

Figure S3. HMBC spectrum- Compound **8**

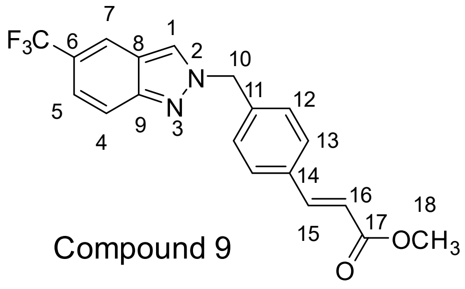

Compound 9

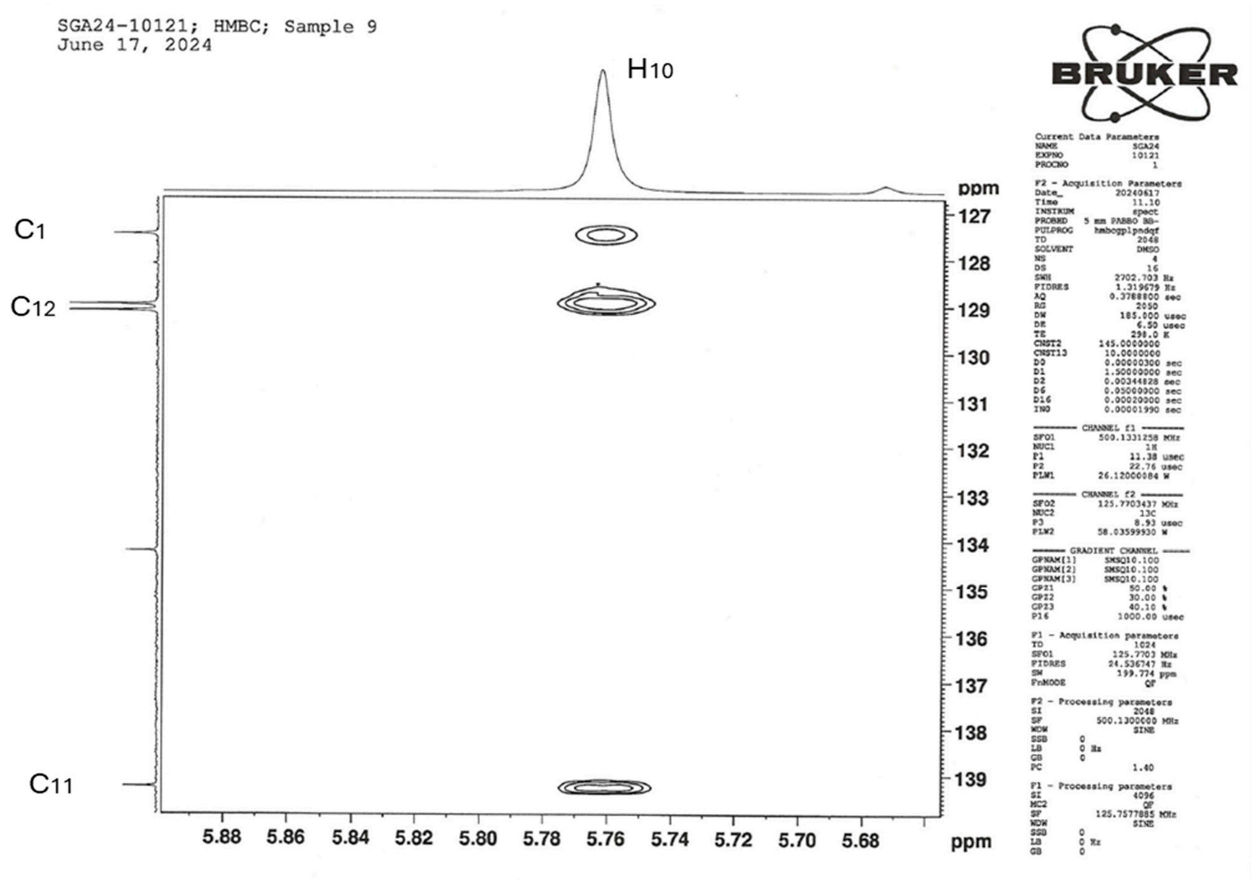

Figure S4. HMBC spectrum- Compound **9**

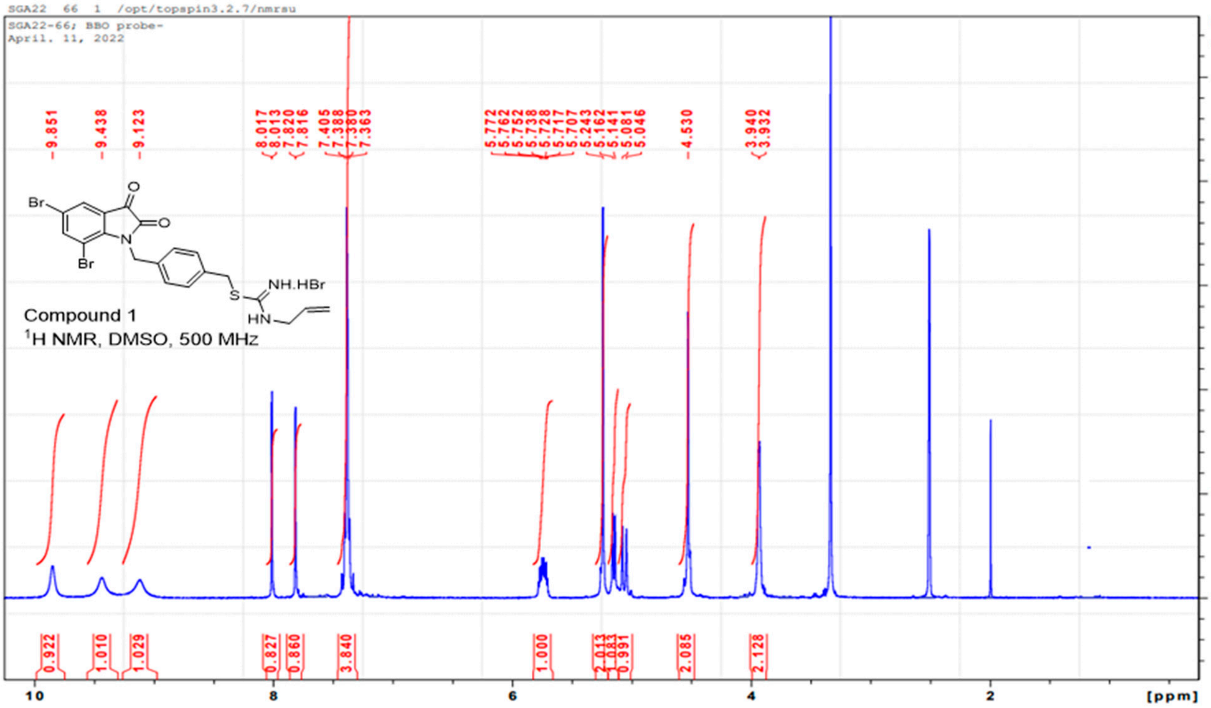
SGA22 66 1 /opt/topspin3.2.7/nmrsu
SGA22-66; BBO probe-
April. 11, 2022
Compound 1
^{1}H NMR, DMSO, 500 MHz
9.851
9.438
9.123
8.017
8.013
7.820
7.816
7.405
7.388
7.380
7.363
5.772
5.762
5.752
5.738
5.728
5.717
5.707
5.243
5.162
5.141
5.081
5.046
4.530
3.940
3.932
0.922
1.010
1.029
0.827
0.860
3.840
1.000
2.013
1.083
0.991
2.085
2.128
[ppm]
[rel]

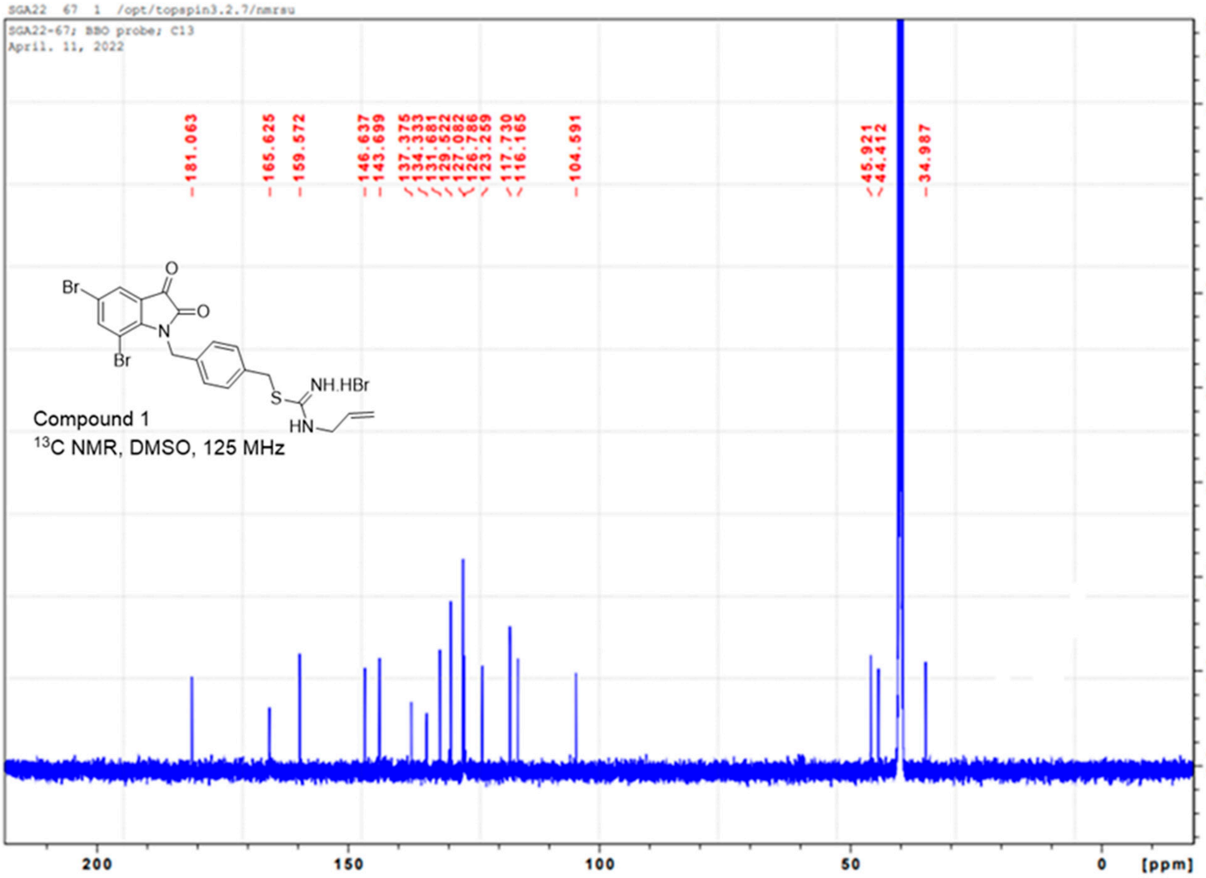
SGA22 67 1 /opt/topspin3.2.7/nmrsu
SGA22-67; BBO probe; C13
April. 11, 2022
Compound 1
^{13}C NMR, DMSO, 125 MHz
181.063
165.625
159.572
146.637
143.699
137.375
134.333
131.681
129.522
127.082
126.786
123.259
117.730
116.165
104.591
45.921
44.412
34.987
[ppm]
[rel]

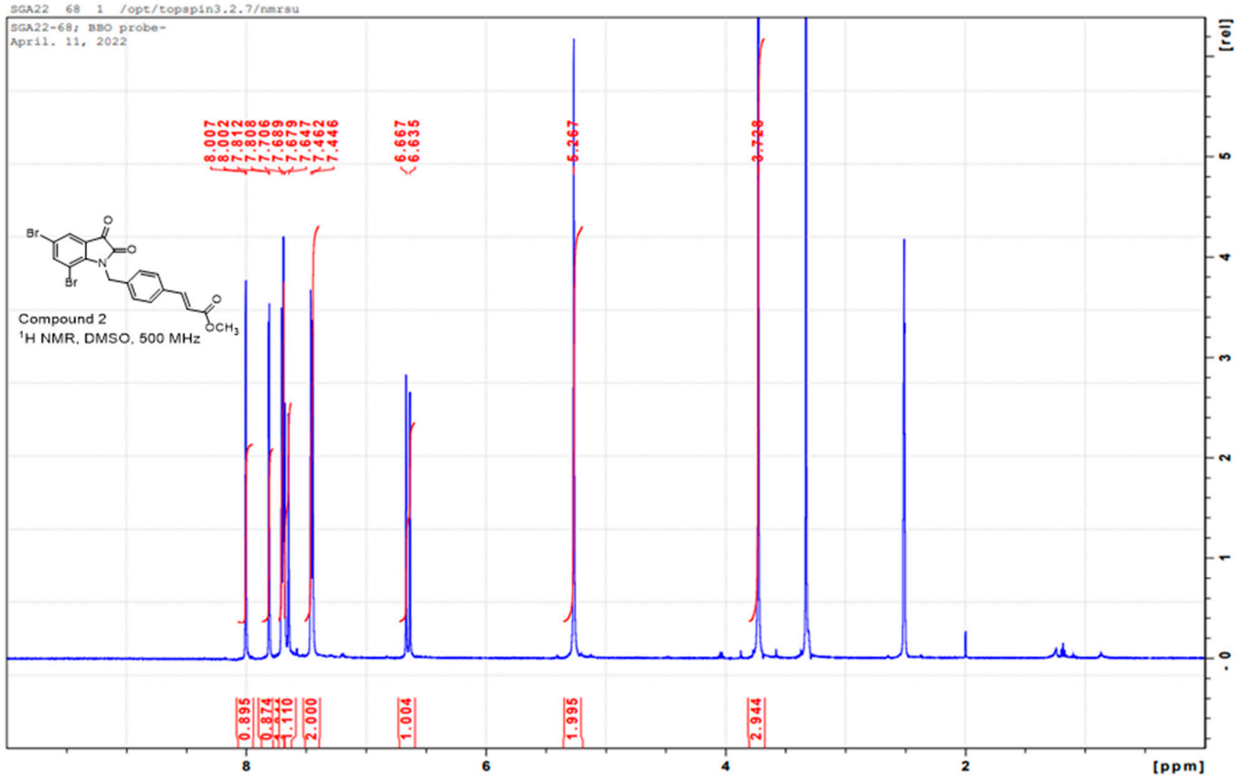
SGA22 68 1 /opt/topspin3.2.7/nmrsu
SGA22-68; BBO probe-
April. 11, 2022
8.007
8.002
7.812
7.808
7.706
7.689
7.679
7.647
7.462
7.446
6.667
6.635
5.267
3.728
Compound 2
1H NMR, DMSO, 500 MHz
0.895
0.874
1.110
2.000
1.004
1.995
2.944
[ppm]
[rel]

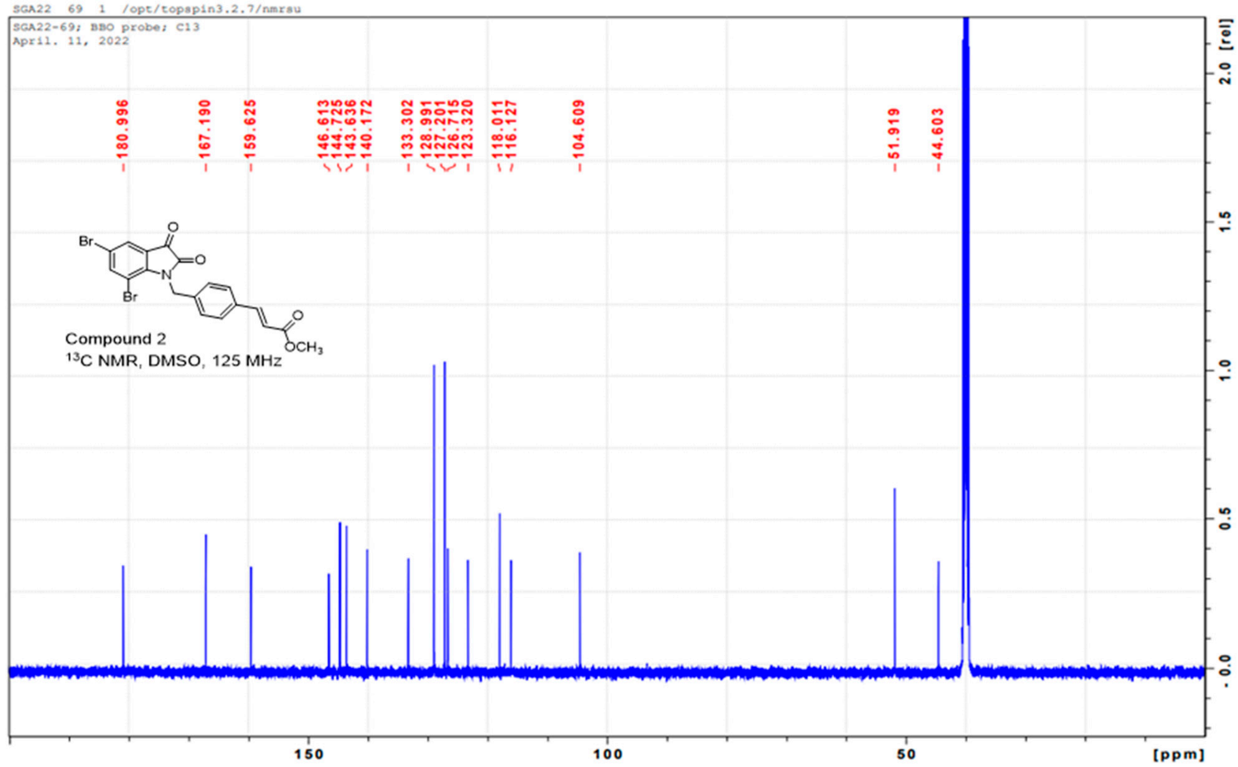
SGA22 69 1 /opt/topspin3.2.7/nmrsu
SGA22-69; BBO probe; C13
April. 11, 2022
180.996
167.190
159.625
146.613
144.725
143.636
140.172
133.302
128.991
127.201
126.715
123.320
118.011
116.127
104.609
51.919
44.603
Compound 2
13C NMR, DMSO, 125 MHz
[ppm]
[rel]

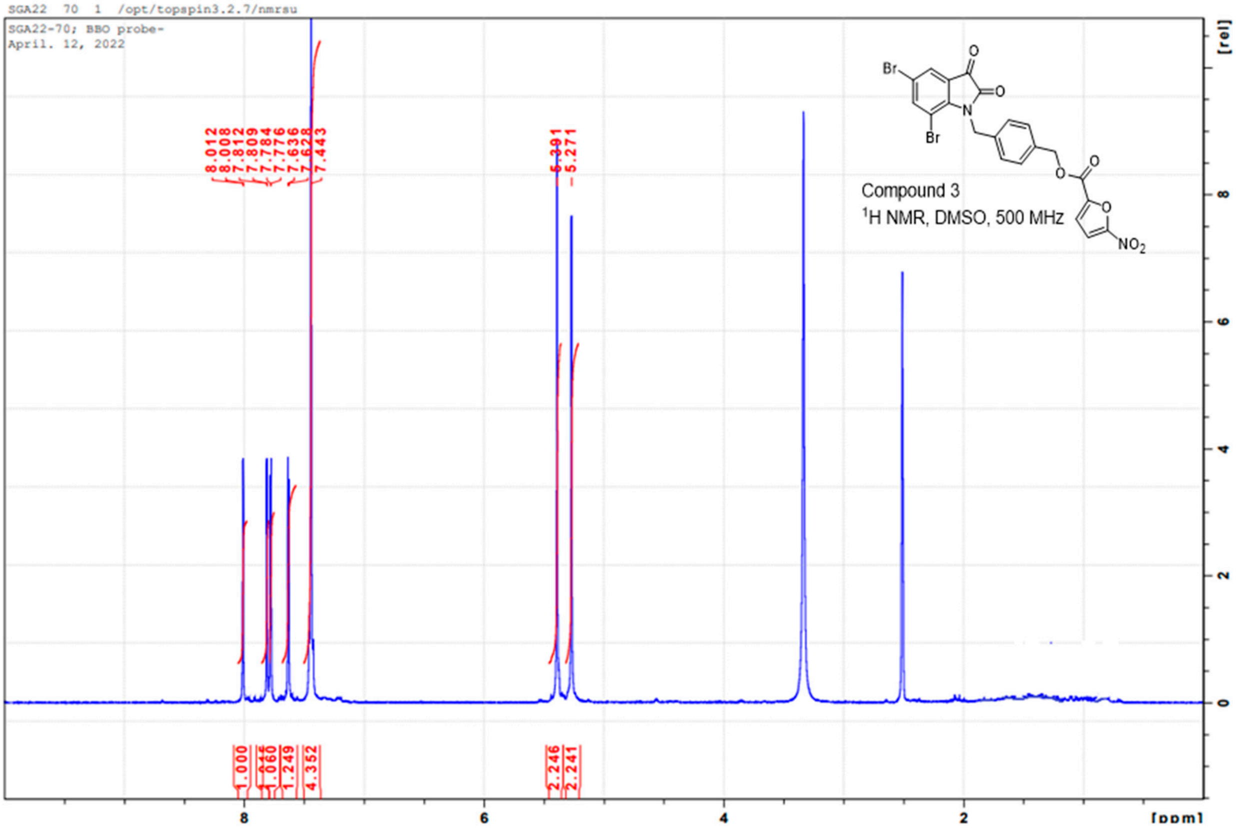
SGA22 70 1 /opt/topspin3.2.7/nmrsu
SGA22-70; BBO probe-
April. 12, 2022
8.012
8.008
7.812
7.809
7.784
7.776
7.636
7.628
7.443
5.271
1.000
1.060
1.249
4.352
2.246
2.241
Compound 3
1H NMR, DMSO, 500 MHz
[rel]
[ppm]

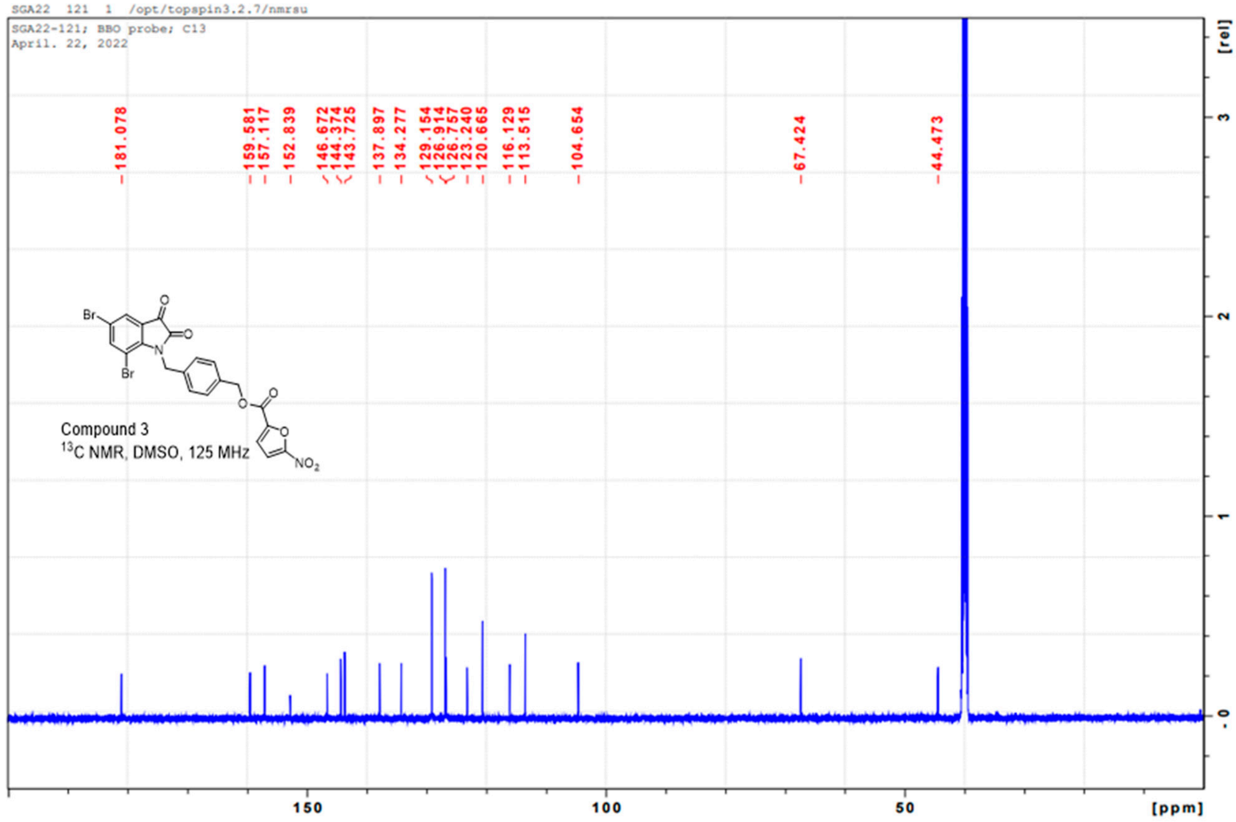
SGA22 121 1 /opt/topspin3.2.7/nmrsu
SGA22-121; BBO probe; C13
April. 22, 2022
181.078
159.581
157.117
152.839
146.672
144.374
143.725
137.897
134.277
129.154
126.914
126.757
123.240
120.665
116.129
113.515
104.654
67.424
44.473
Compound 3
13C NMR, DMSO, 125 MHz
[rel]
[ppm]

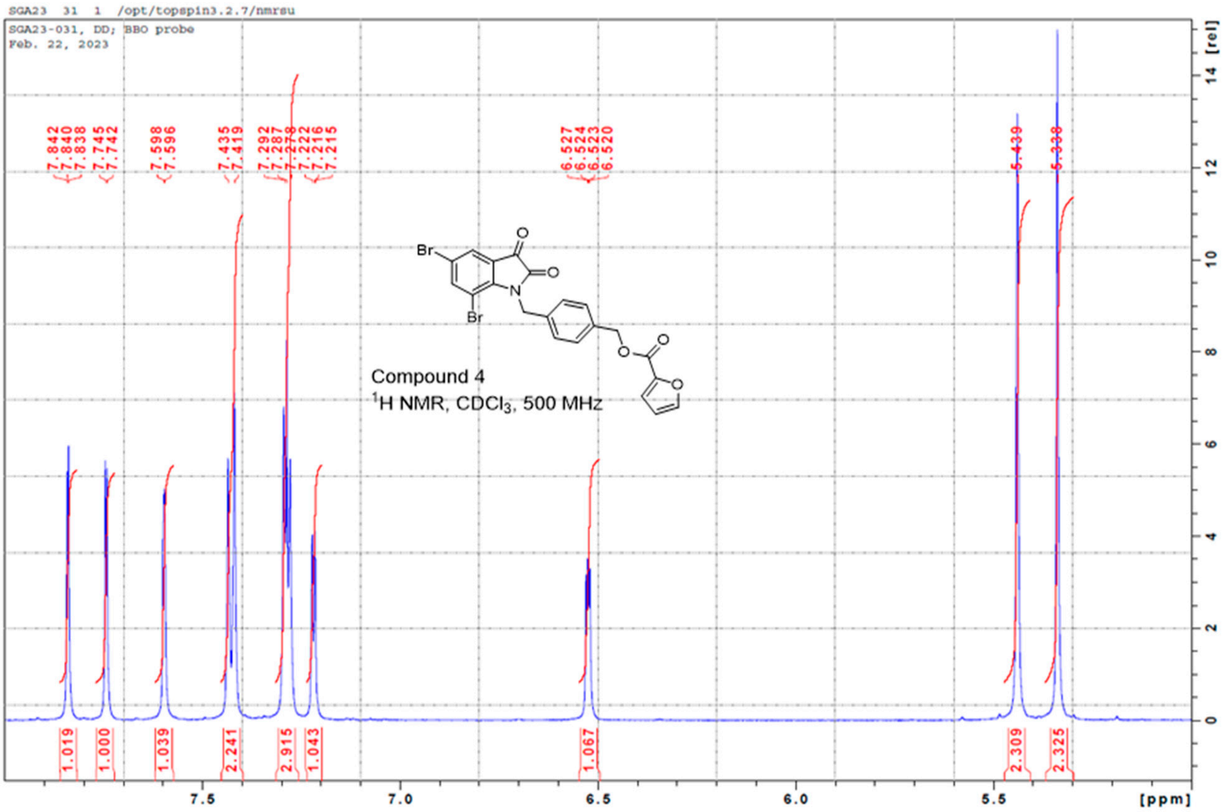

SGA23 31 1 /opt/topspin3.2.7/nmrsu
SGA23-031, DD; BBO probe
Feb. 22, 2023
7.842
7.840
7.838
7.745
7.742
7.598
7.596
7.435
7.419
7.292
7.287
7.222
7.216
7.215
6.527
6.524
6.523
6.520
5.439
5.338
Br
O
O
N
Br
O
O
O
Compound 4
¹H NMR, CDCl₃, 500 MHz
1.019
1.000
1.039
2.241
2.915
1.043
1.067
2.309
2.325
7.5
7.0
6.5
6.0
5.5
[ppm]
[rel]

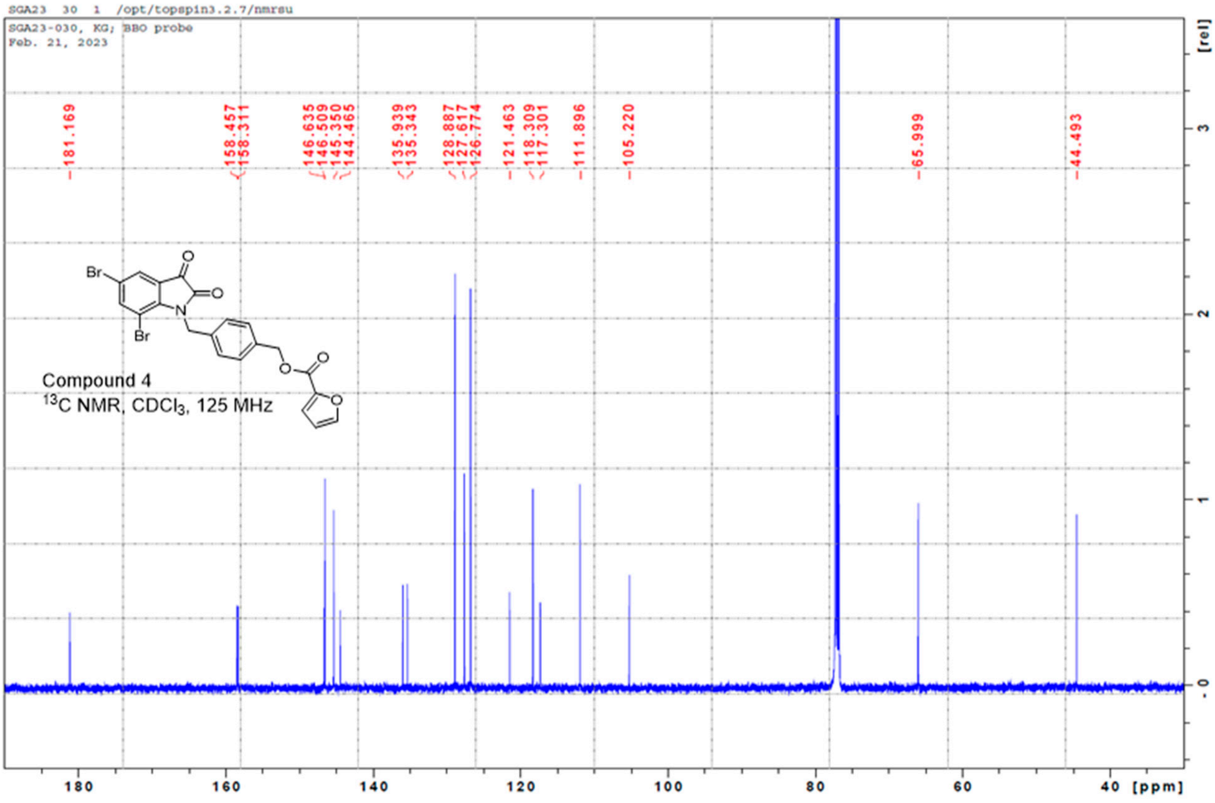

SGA23 30 1 /opt/topspin3.2.7/nmrsu
SGA23-030, KG; BBO probe
Feb. 21, 2023
181.169
158.457
158.311
146.635
146.509
145.350
144.465
135.939
135.343
128.887
127.617
126.774
121.463
118.309
117.301
111.896
105.220
65.999
44.493
Br
O
O
N
Br
O
O
O
Compound 4
¹³C NMR, CDCl₃, 125 MHz
180
160
140
120
100
80
60
40
[ppm]
[rel]

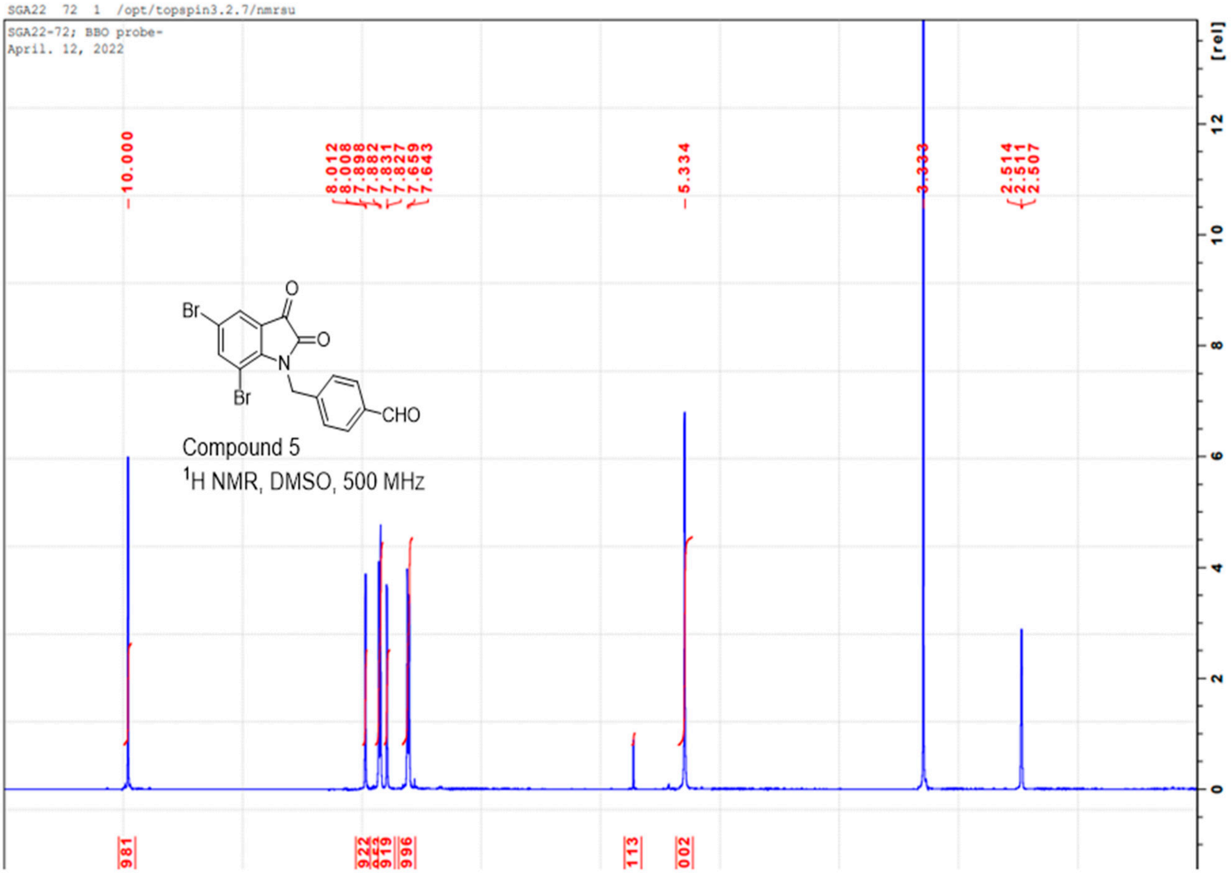
SGA22 72 1 /opt/topspin3.2.7/nmrsu
SGA22-72; BBO probe-
April. 12, 2022
10.000
8.012
8.008
7.898
7.882
7.831
7.827
7.659
7.643
5.334
3.333
2.514
2.511
2.507
Br
Br
O
O
N
CHO
Compound 5
1H NMR, DMSO, 500 MHz
[rel]

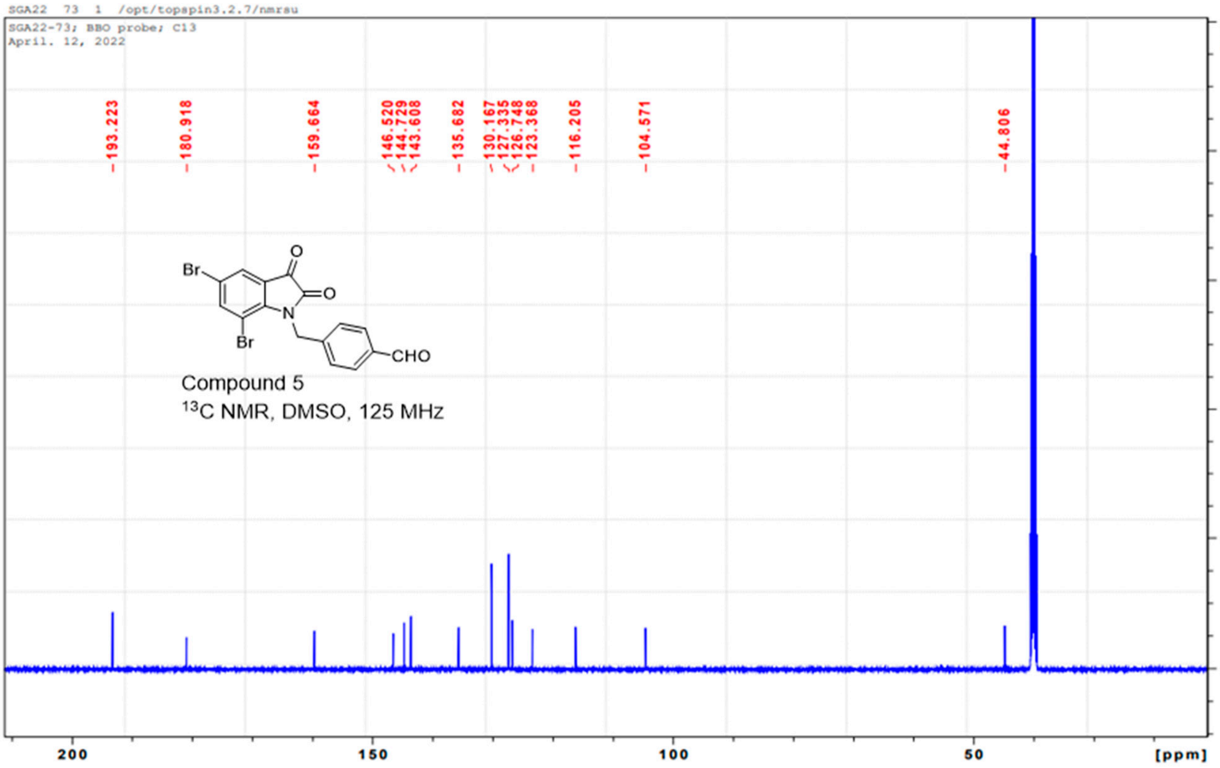
SGA22 73 1 /opt/topspin3.2.7/nmrsu
SGA22-73; BBO probe; C13
April. 12, 2022
193.223
180.918
159.664
146.520
144.729
143.608
135.682
130.167
127.335
126.748
123.368
116.205
104.571
44.806
Br
Br
O
O
N
CHO
Compound 5
13C NMR, DMSO, 125 MHz
200
150
100
50
[ppm]
[rel]

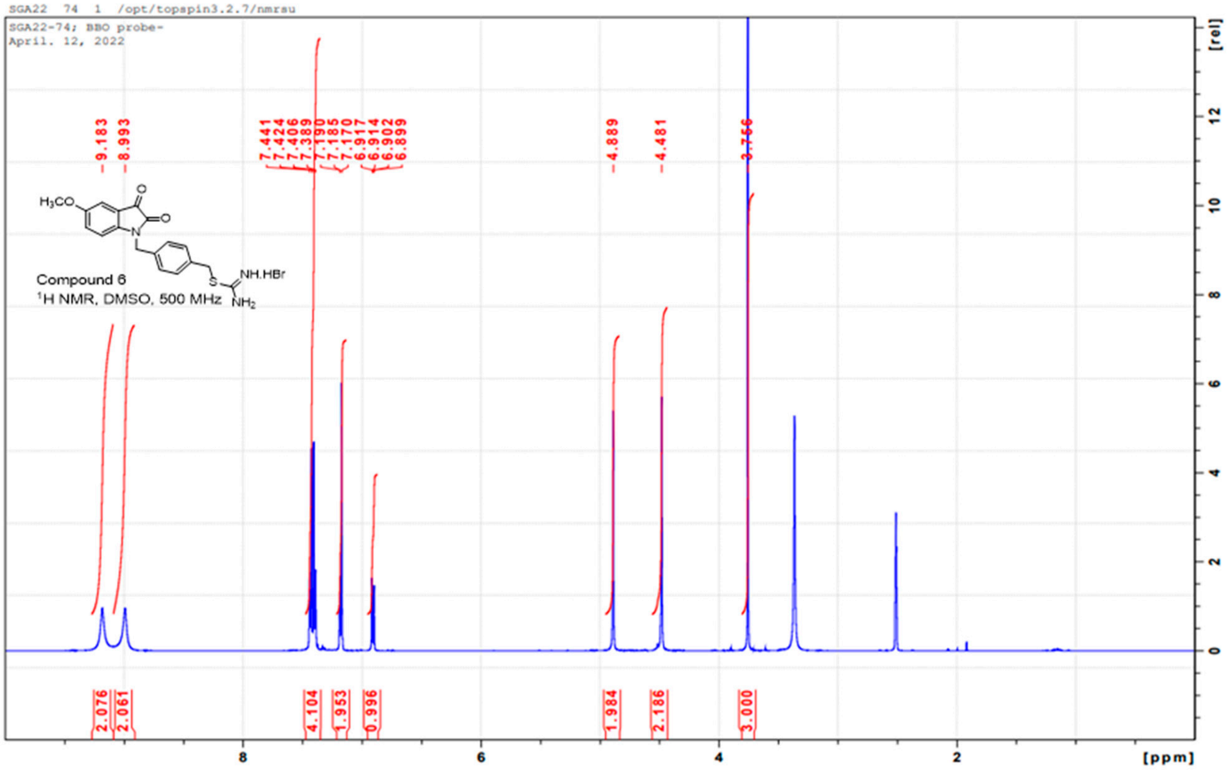

SGA22 74 1 /opt/topspin3.2.7/nmrsu
SGA22-74; BBO probe-
April. 12, 2022
Compound 6
^{1}H NMR, DMSO, 500 MHz
9.183
8.993
7.441
7.424
7.406
7.389
7.190
7.185
7.170
6.917
6.914
6.902
6.899
4.889
4.481
3.756
2.076
2.061
4.104
1.953
0.996
1.984
2.186
3.000
[ppm]
[rel]

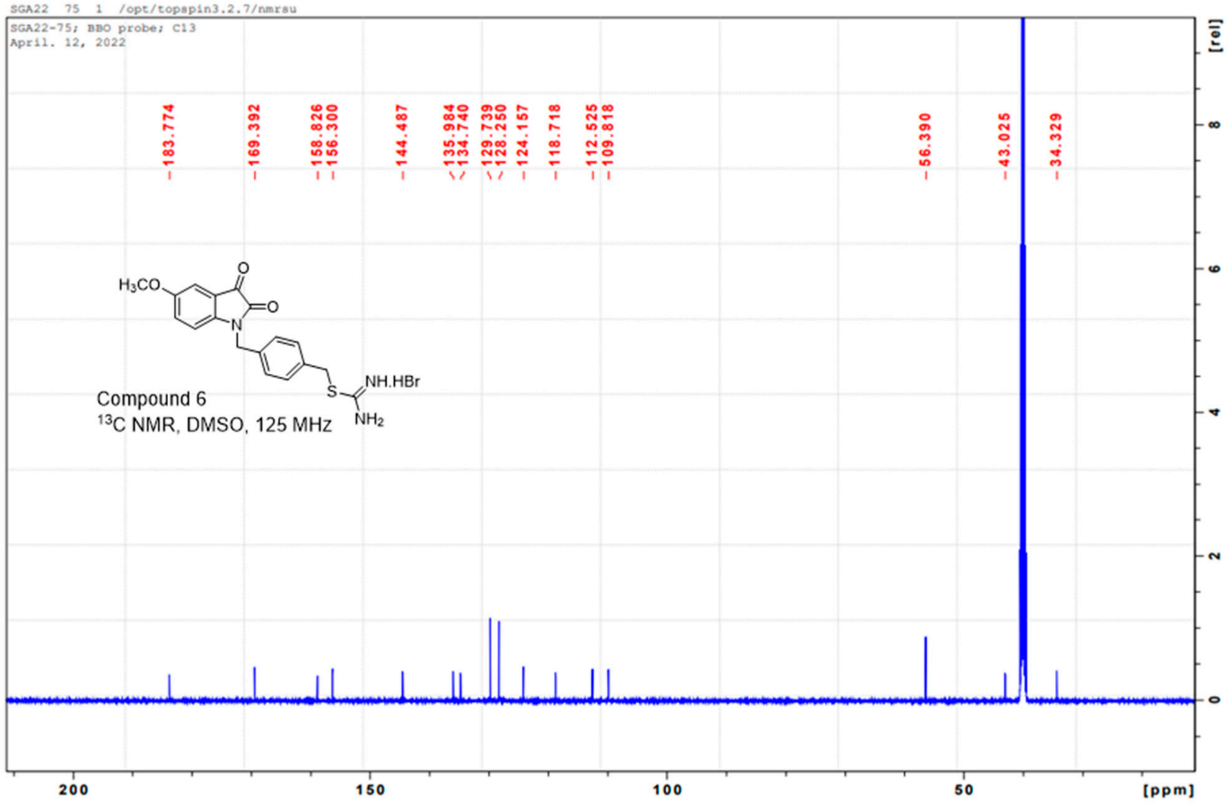

SGA22 75 1 /opt/topspin3.2.7/nmrsu
SGA22-75; BBO probe; C13
April. 12, 2022
Compound 6
^{13}C NMR, DMSO, 125 MHz
183.774
169.392
158.826
156.300
144.487
135.984
134.740
129.739
128.250
124.157
118.718
112.525
109.818
56.390
43.025
34.329
[ppm]
[rel]

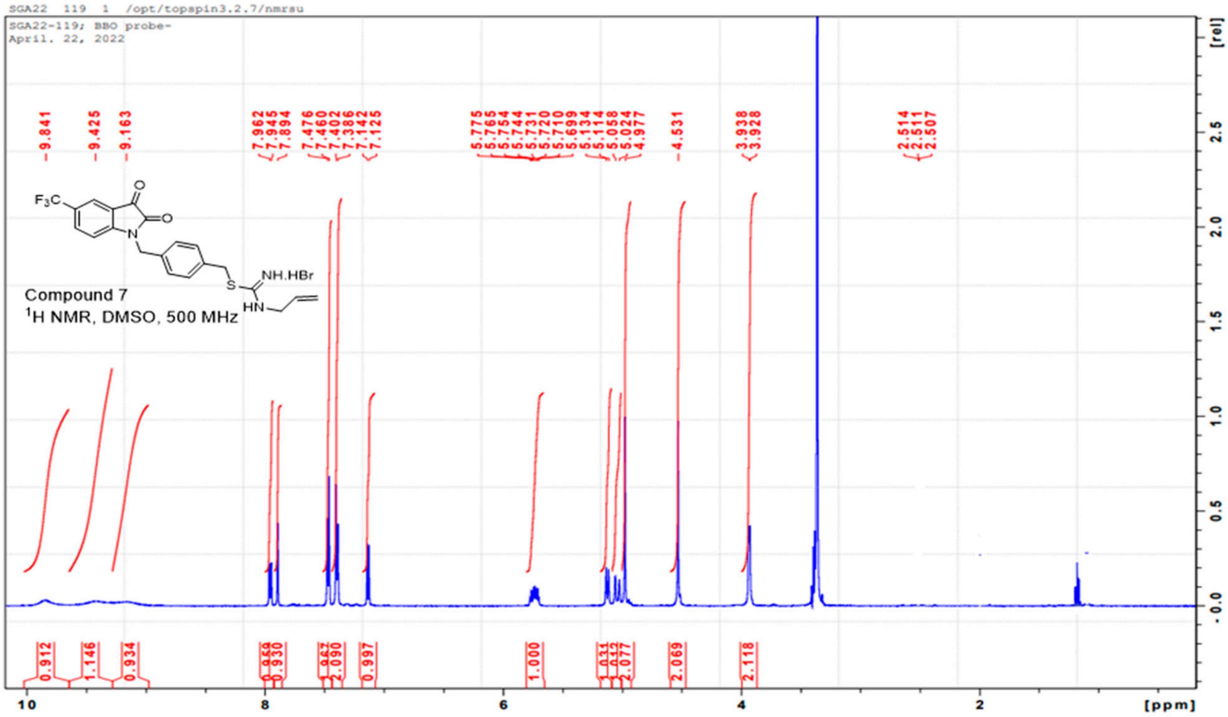
SGA22 119 1 /opt/topspin3.2.7/nmrsu
SGA22-119; BBO probe-
April. 22, 2022
Compound 7
^{1}H NMR, DMSO, 500 MHz
F$_3$C
NH.HBr
HN
9.841
9.425
9.163
7.962
7.945
7.894
7.476
7.460
7.402
7.386
7.142
7.125
5.775
5.765
5.754
5.744
5.731
5.720
5.710
5.699
5.134
5.114
5.058
5.024
4.977
4.531
3.938
3.928
2.514
2.511
2.507
0.912
1.146
0.934
0.956
0.930
1.967
2.090
0.997
1.000
1.031
1.012
2.077
2.069
2.118
[ppm]
[rel]

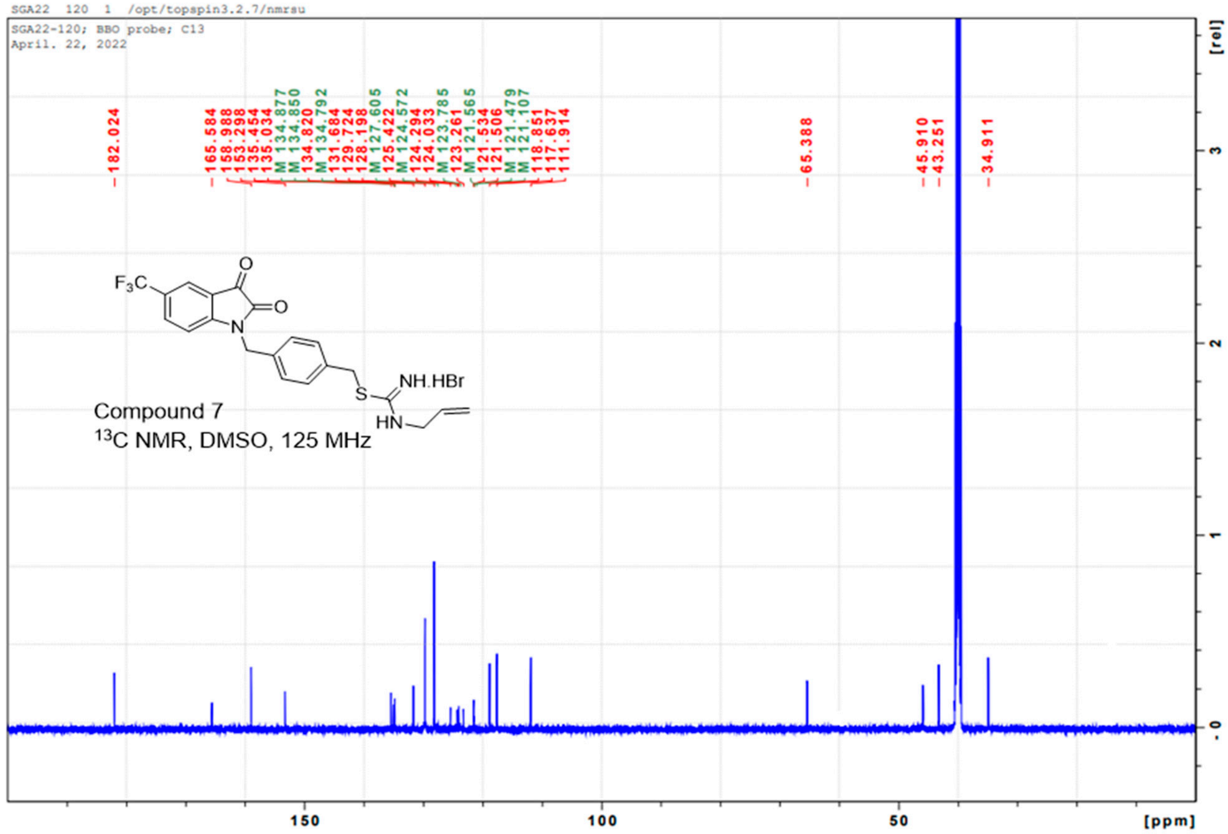
SGA22 120 1 /opt/topspin3.2.7/nmrsu
SGA22-120; BBO probe; C13
April. 22, 2022
Compound 7
^{13}C NMR, DMSO, 125 MHz
F$_3$C
NH.HBr
HN
182.024
165.584
158.988
153.298
135.454
135.034
M 134.877
M 134.850
M 134.820
M 134.792
131.684
129.724
128.198
M 127.605
M 125.422
M 124.572
124.094
124.033
M 123.785
M 123.261
M 121.565
121.534
121.506
M 121.479
M 121.107
118.851
117.637
111.914
65.388
45.910
43.251
34.911
[ppm]
[rel]

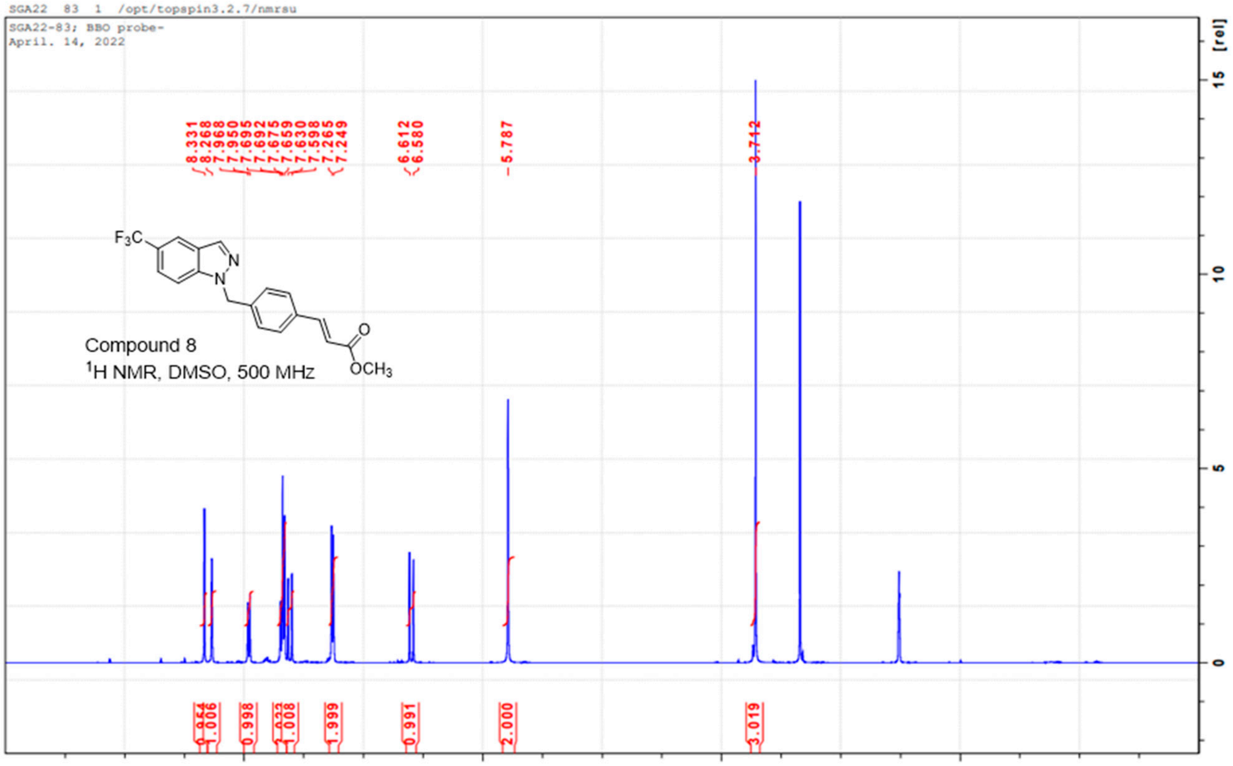
SGA22 83 1 /opt/topspin3.2.7/nmrsu
SGA22-83; BBO probe-
April. 14, 2022
Compound 8
^{1}H NMR, DMSO, 500 MHz
8.331
8.268
7.968
7.950
7.695
7.692
7.675
7.659
7.630
7.598
7.265
7.249
6.612
6.580
5.787
3.712
0.964
1.006
0.998
1.023
1.008
1.999
0.991
2.000
3.019
[rel]

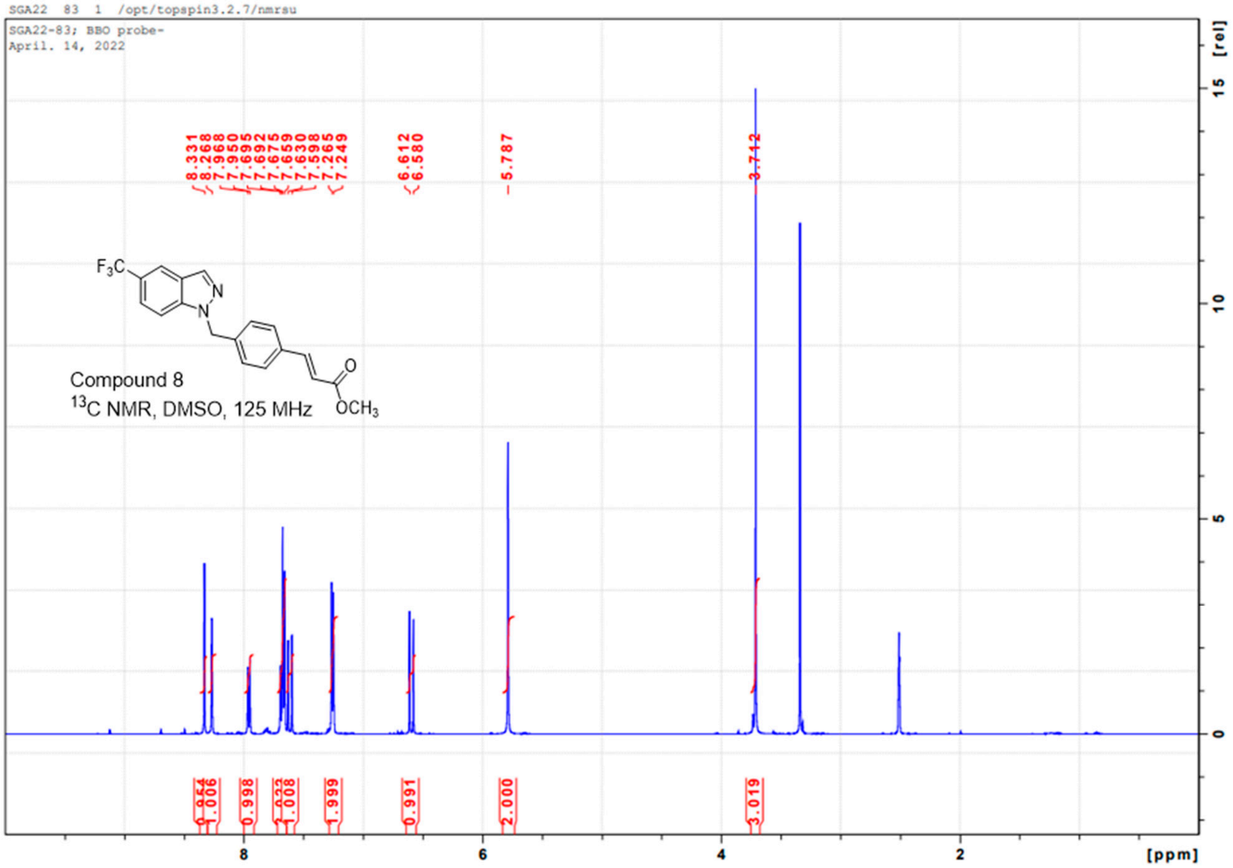
SGA22 83 1 /opt/topspin3.2.7/nmrsu
SGA22-83; BBO probe-
April. 14, 2022
Compound 8
^{13}C NMR, DMSO, 125 MHz
[rel]
[ppm]

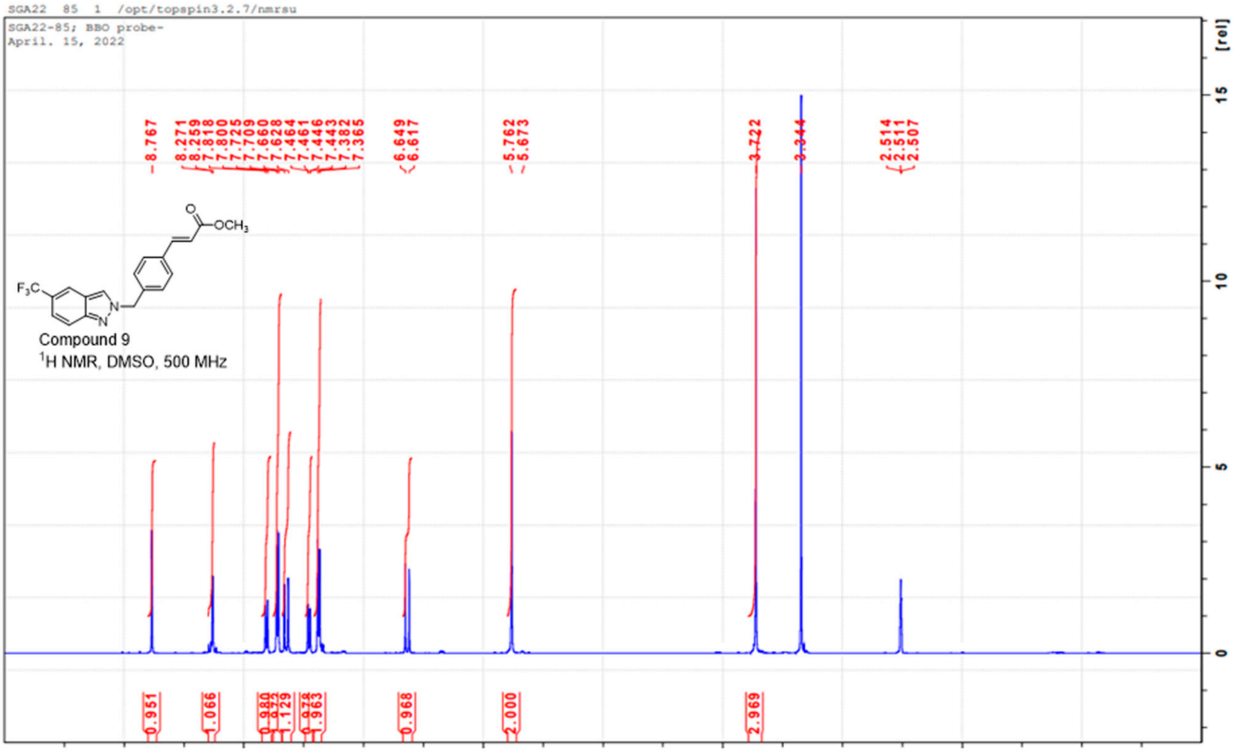
SGA22 85 1 /opt/topspin3.2.7/nmrsu
SGA22-85; BBO probe-
April. 15, 2022
8.767
8.271 8.259 7.818 7.800 7.725 7.709 7.660 7.628 7.464 7.461 7.446 7.443 7.382 7.365
6.649 6.617
5.762 5.673
3.722
3.344
2.514 2.511 2.507
OCH3
F3C
N
Compound 9
1H NMR, DMSO, 500 MHz
0.951
1.066
0.980
1.129
0.974
1.963
0.968
2.000
2.969
[rel]

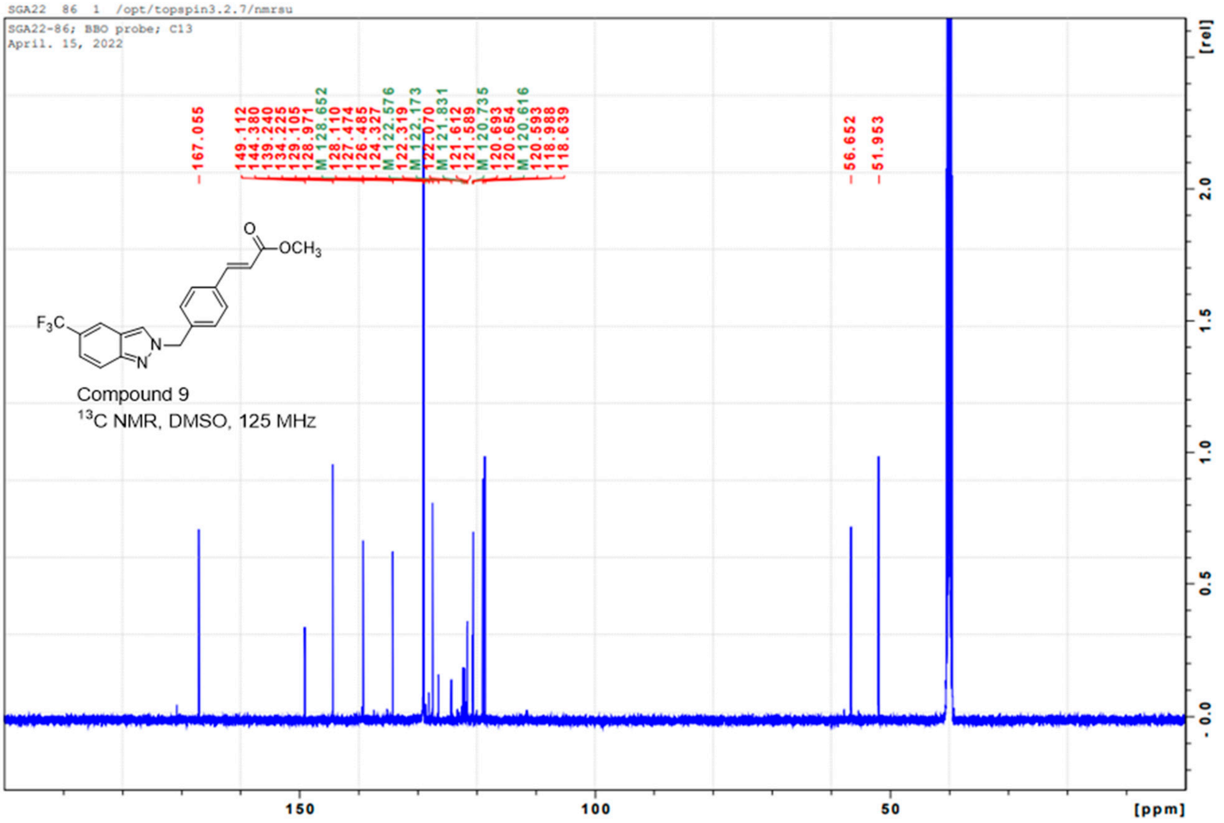
SGA22 86 1 /opt/topspin3.2.7/nmrsu
SGA22-86; BBO probe; C13
April. 15, 2022
167.055
149.112 144.380 139.240 134.225 129.105 128.971 M 128.652 128.110 127.474 126.485 124.327 M 122.576 122.319 M 122.173 122.070 M 121.831 121.612 121.589 M 120.735 120.693 120.654 M 120.616 120.593 118.988 118.639
56.652
51.953
OCH3
F3C
N
Compound 9
13C NMR, DMSO, 125 MHz
[rel]
150
100
50
[ppm]

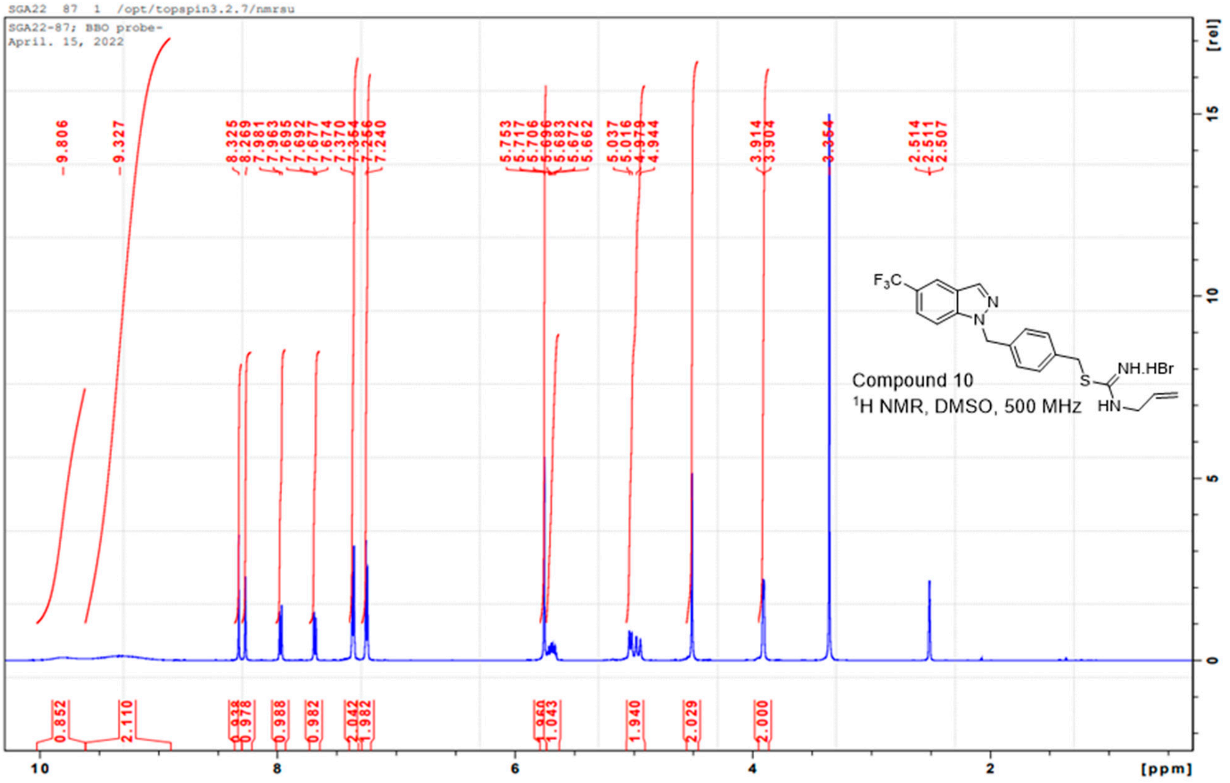
SGA22 87 1 /opt/topspin3.2.7/nmrsu
SGA22-87; BBO probe-
April. 15, 2022
F3C
N
N
NH.HBr
S
HN
Compound 10
1H NMR, DMSO, 500 MHz
[rel]
[ppm]

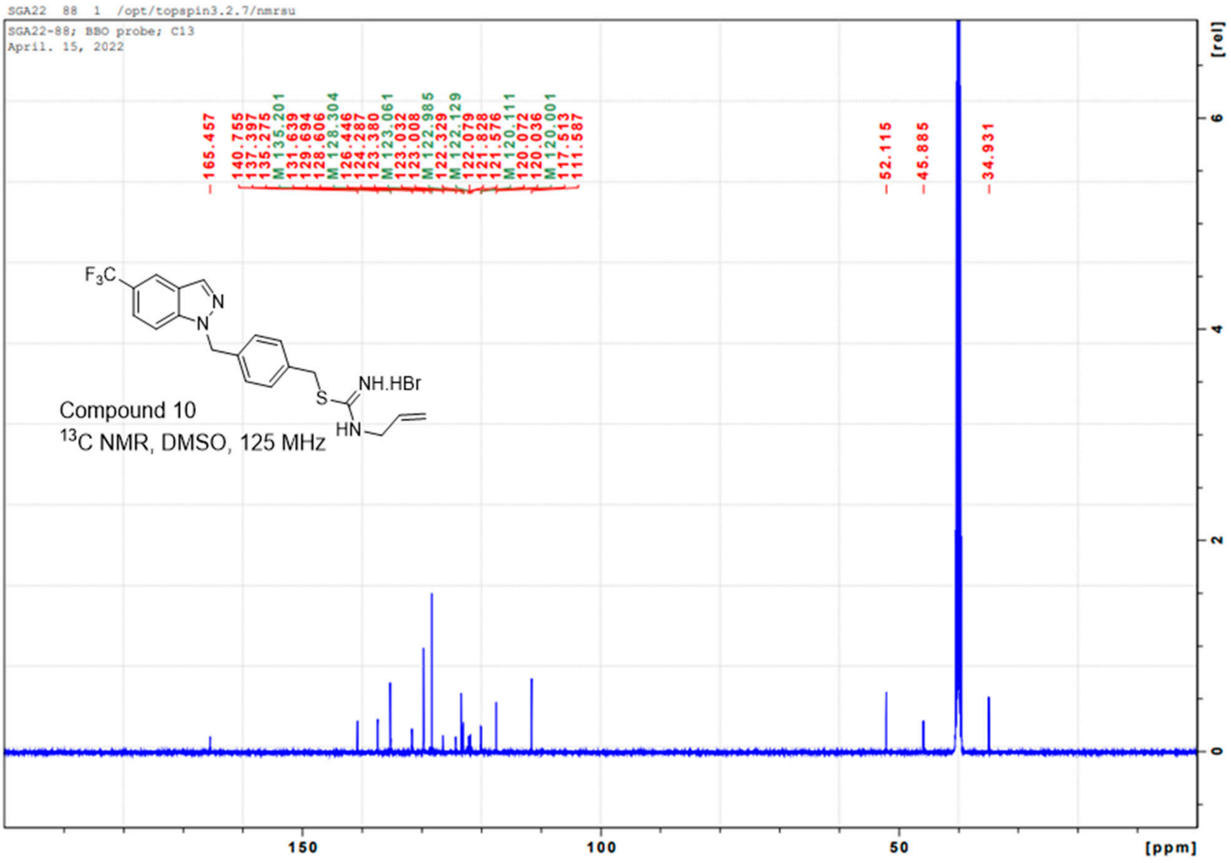
SGA22 88 1 /opt/topspin3.2.7/nmrsu
SGA22-88; BBO probe; C13
April. 15, 2022
F3C
N
N
NH.HBr
S
HN
Compound 10
13C NMR, DMSO, 125 MHz
[rel]
[ppm]

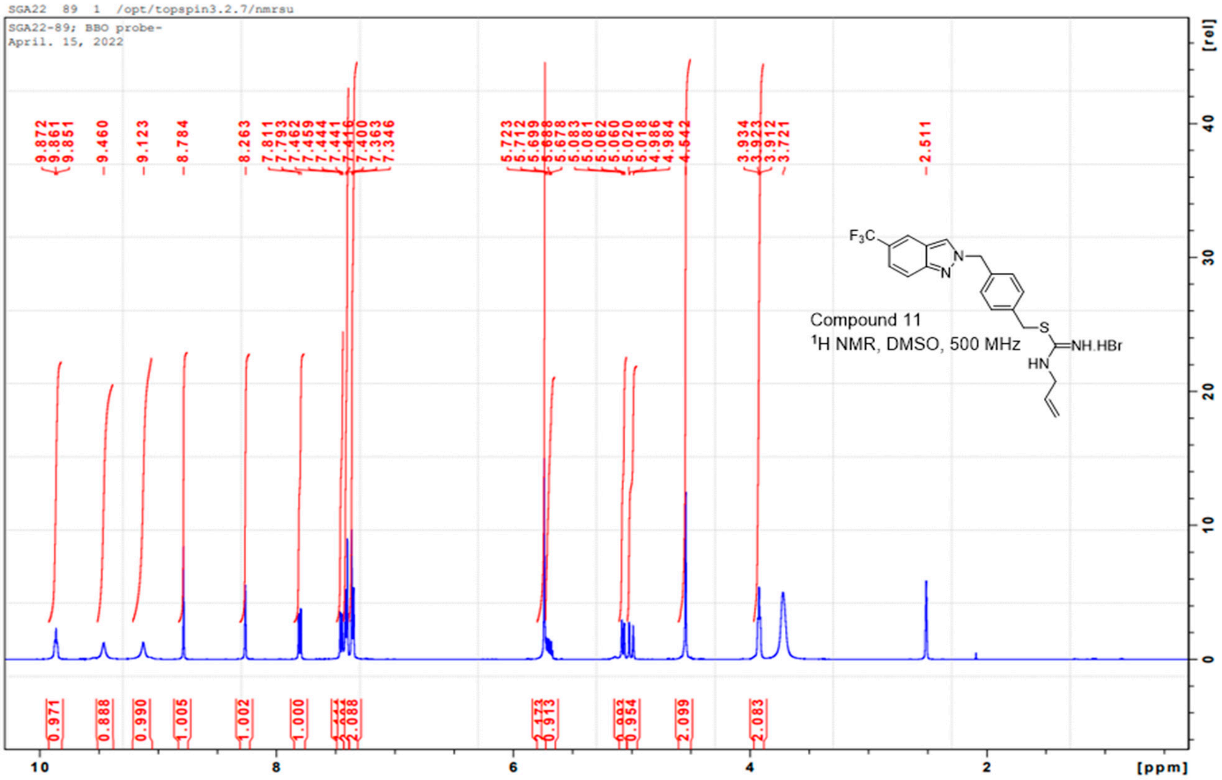
SGA22 89 1 /opt/topspin3.2.7/nmrsu
SGA22-89; BBO probe-
April. 15, 2022
Compound 11
^{1}H NMR, DMSO, 500 MHz
F3C
N
NH.HBr
HN
S
9.872 9.861 9.851 9.460 9.123 8.784 8.263 7.811 7.793 7.462 7.459 7.444 7.441 7.416 7.400 7.363 7.346
5.723 5.712 5.699 5.688 5.678 5.083 5.081 5.062 5.060 5.020 5.018 4.986 4.984 4.542
3.934 3.923 3.912 3.721 2.511
0.971 0.888 0.990 1.005 1.002 1.000 2.088 0.913 0.954 2.099 2.083
[ppm]
[rel]

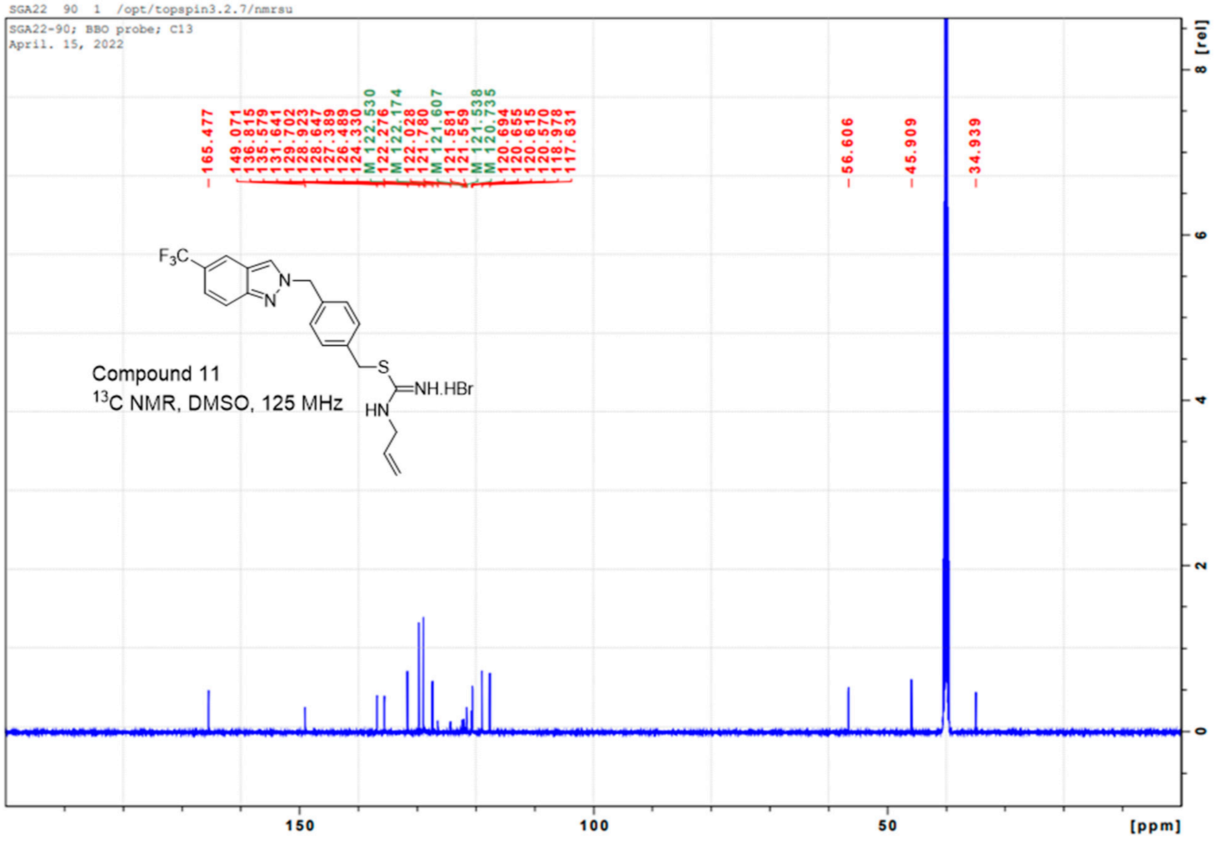
SGA22 90 1 /opt/topspin3.2.7/nmrsu
SGA22-90; BBO probe; C13
April. 15, 2022
Compound 11
^{13}C NMR, DMSO, 125 MHz
F3C
N
NH.HBr
HN
S
165.477 149.071 136.815 135.579 131.641 129.702 128.923 128.647 127.389 126.489 124.330 M 122.530 122.276 M 122.174 122.028 121.780 M 121.607 121.581 121.559 M 121.538 M 120.735 120.694 120.655 120.615 120.570 118.978 117.631
56.606 45.909 34.939
[ppm]
[rel]

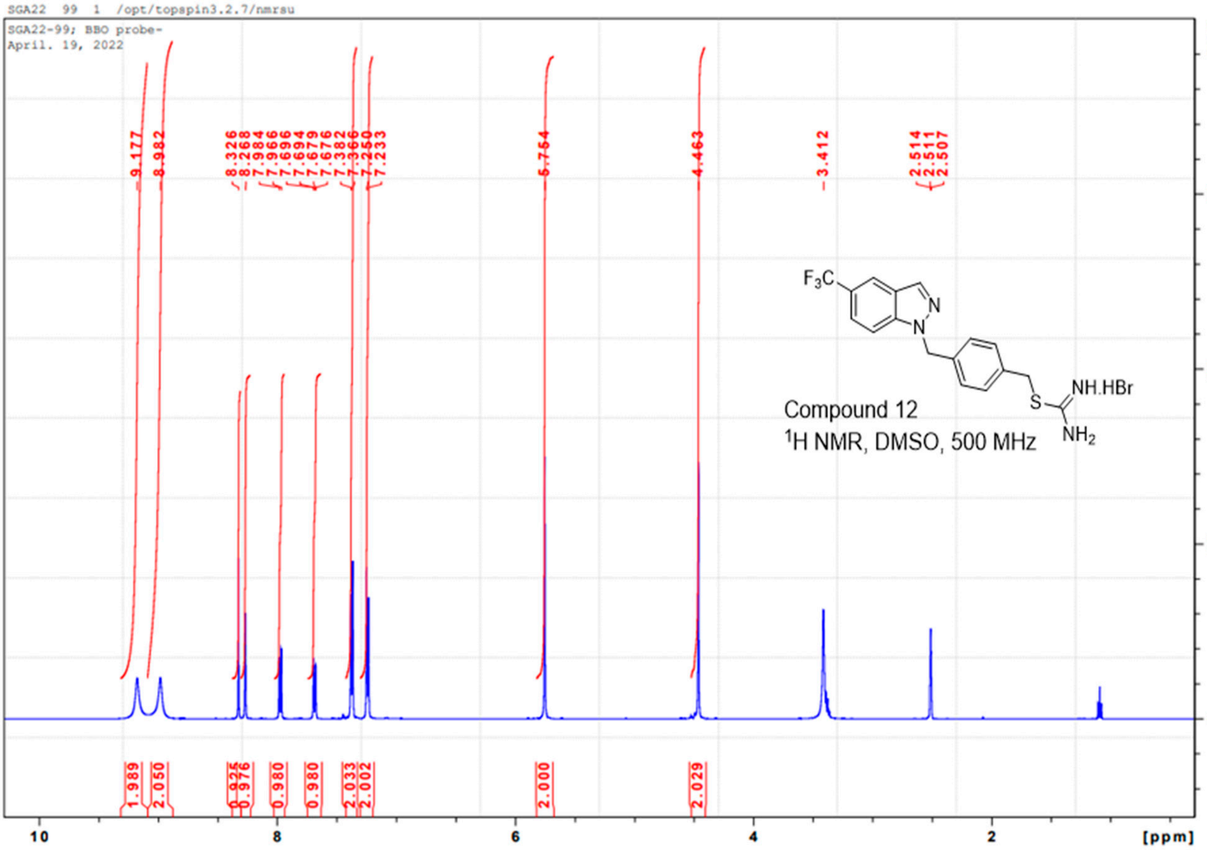
SGA22 99 1 /opt/topspin3.2.7/nmrsu
SGA22-99; BBO probe-
April. 19, 2022
Compound 12
^{1}H NMR, DMSO, 500 MHz
F_3C
N
N
NH.HBr
S
NH_2
9.177
8.982
8.326
8.268
7.984
7.966
7.696
7.694
7.679
7.676
7.382
7.366
7.250
7.233
5.754
4.463
3.412
2.514
2.511
2.507
1.989
2.050
0.975
0.976
0.980
0.980
2.033
2.002
2.000
2.029
[ppm]
[rel]

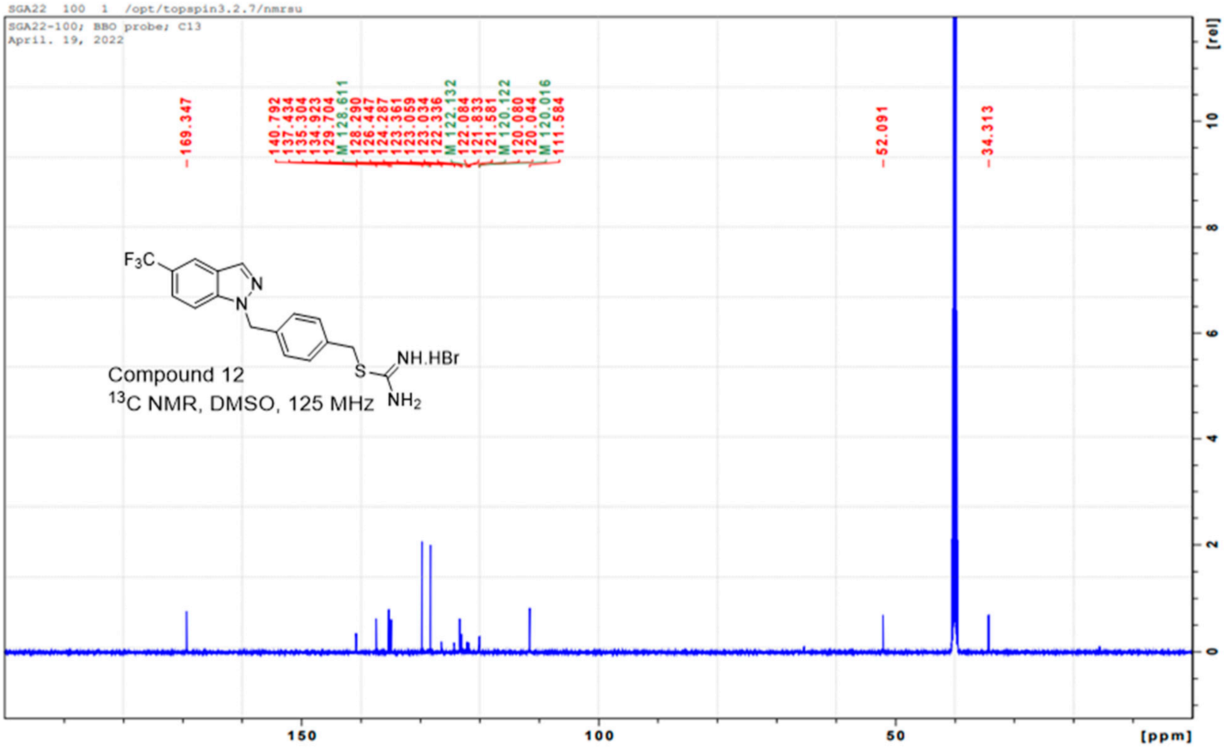
SGA22 100 1 /opt/topspin3.2.7/nmrsu
SGA22-100; BBO probe; C13
April. 19, 2022
Compound 12
^{13}C NMR, DMSO, 125 MHz
F_3C
N
N
NH.HBr
S
NH_2
169.347
140.792
137.434
135.304
134.923
129.704
M 128.611
128.290
126.447
124.287
123.361
123.059
123.034
122.336
M 122.132
122.084
121.833
121.581
M 120.122
120.080
120.044
M 120.016
111.584
52.091
34.313
[ppm]
[rel]

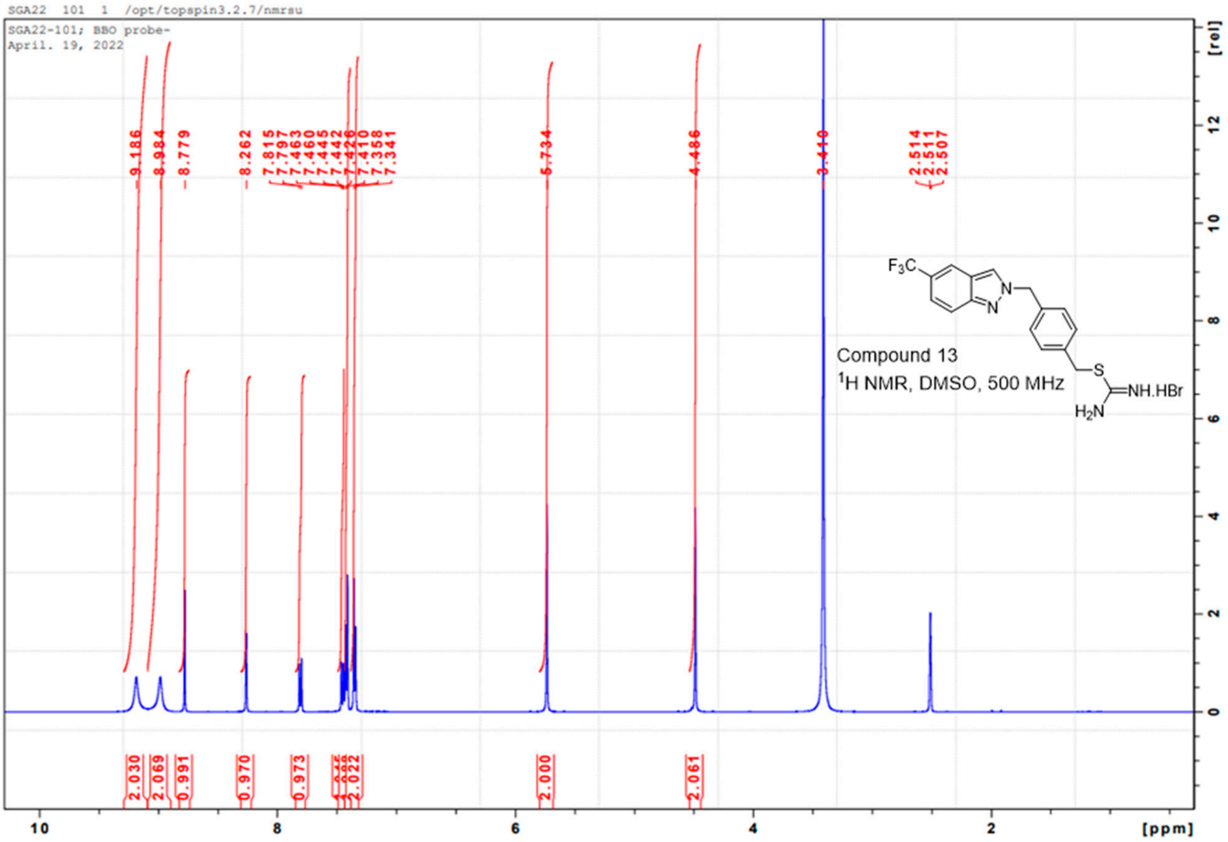
SGA22 101 1 /opt/topspin3.2.7/nmrsu
SGA22-101; BBO probe-
April. 19, 2022
9.186
8.984
8.779
8.262
7.815
7.797
7.463
7.460
7.445
7.442
7.410
7.358
7.341
5.734
4.486
3.410
2.514
2.511
2.507
F3C
N
N
S
NH.HBr
H2N
Compound 13
1H NMR, DMSO, 500 MHz
2.030
2.069
0.991
0.970
0.973
2.022
2.000
2.061
[rel]
[ppm]

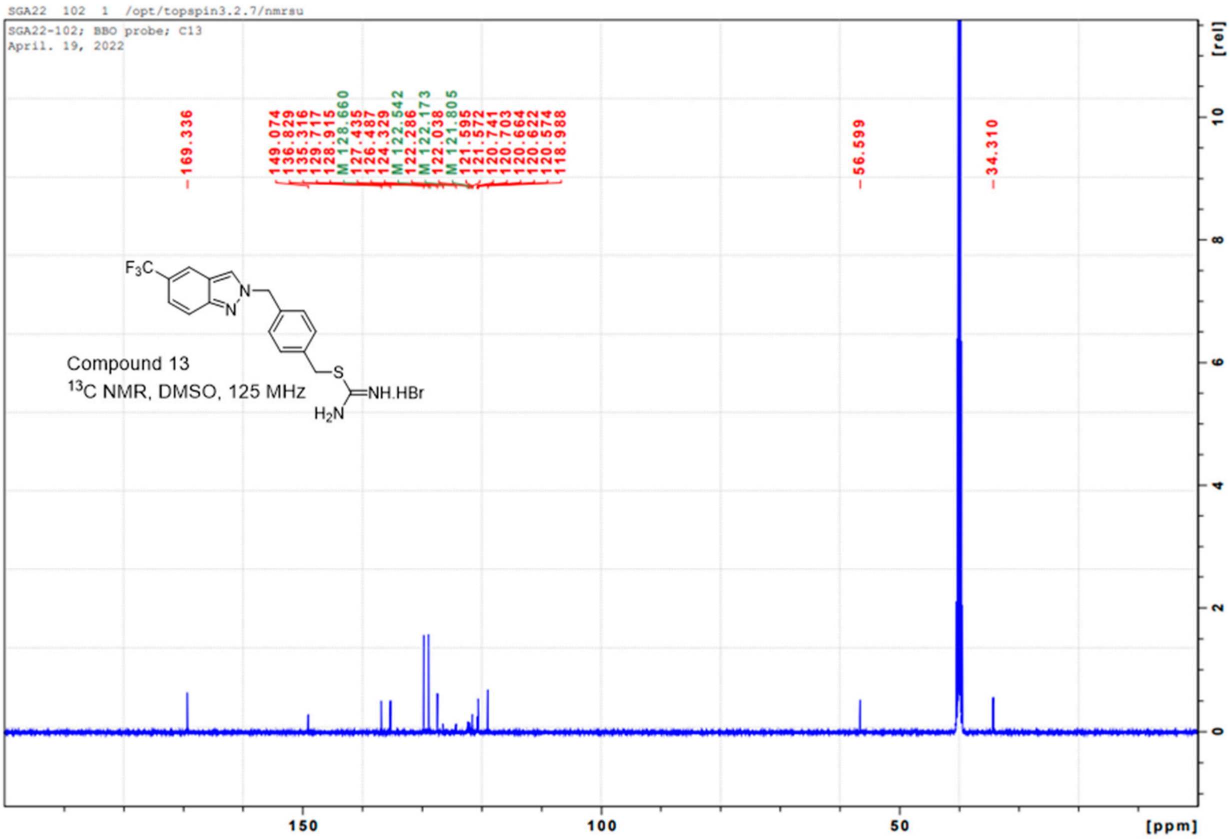
SGA22 102 1 /opt/topspin3.2.7/nmrsu
SGA22-102; BBO probe; C13
April. 19, 2022
169.336
149.074
136.829
135.716
128.915
M 128.660
127.435
126.487
124.329
M 122.542
122.286
M 122.173
122.038
M 121.805
121.595
121.572
120.741
120.703
120.664
120.622
120.574
118.988
56.599
34.310
F3C
N
N
S
NH.HBr
H2N
Compound 13
13C NMR, DMSO, 125 MHz
[rel]
[ppm]

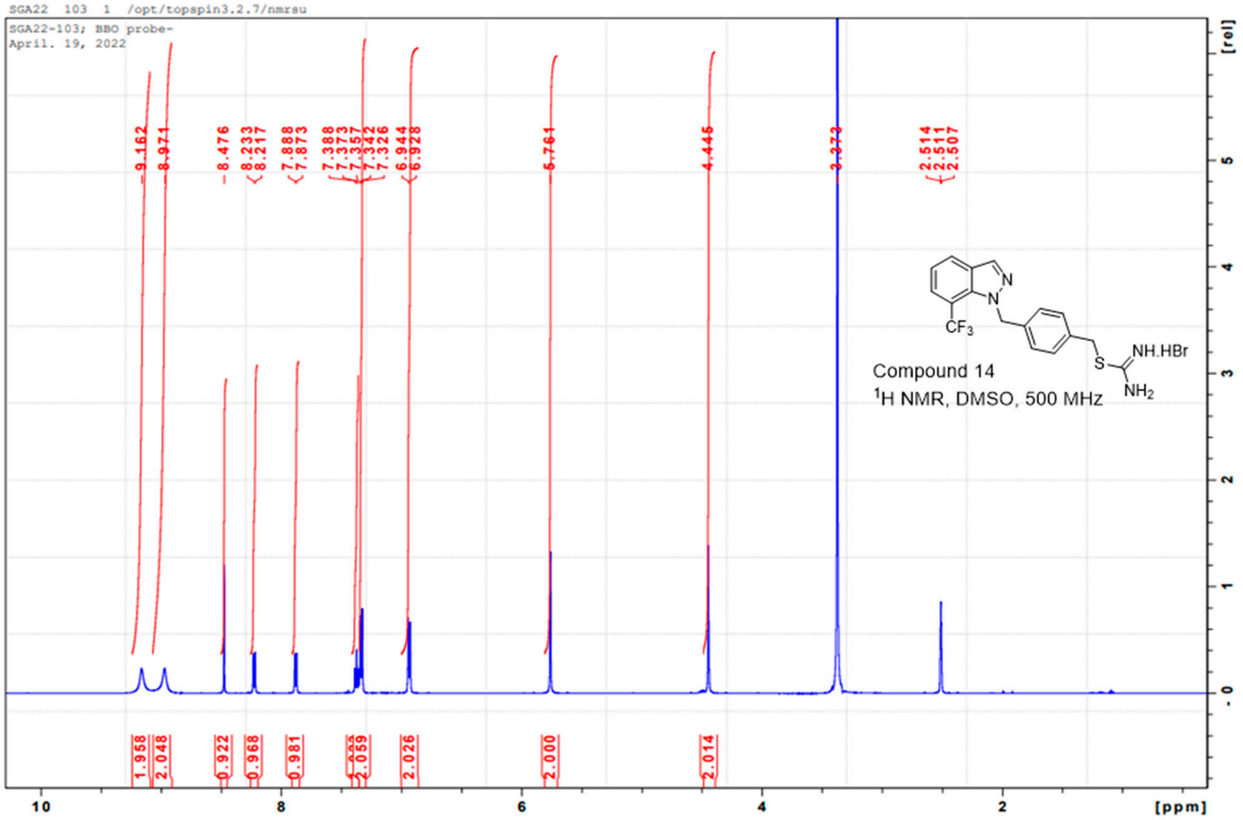

SGA22 103 1 /opt/topspin3.2.7/nmrsu
SGA22-103; BBO probe-
April. 19, 2022
Compound 14
^{1}H NMR, DMSO, 500 MHz
NH.HBr
NH2
CF3
[ppm]
[rel]

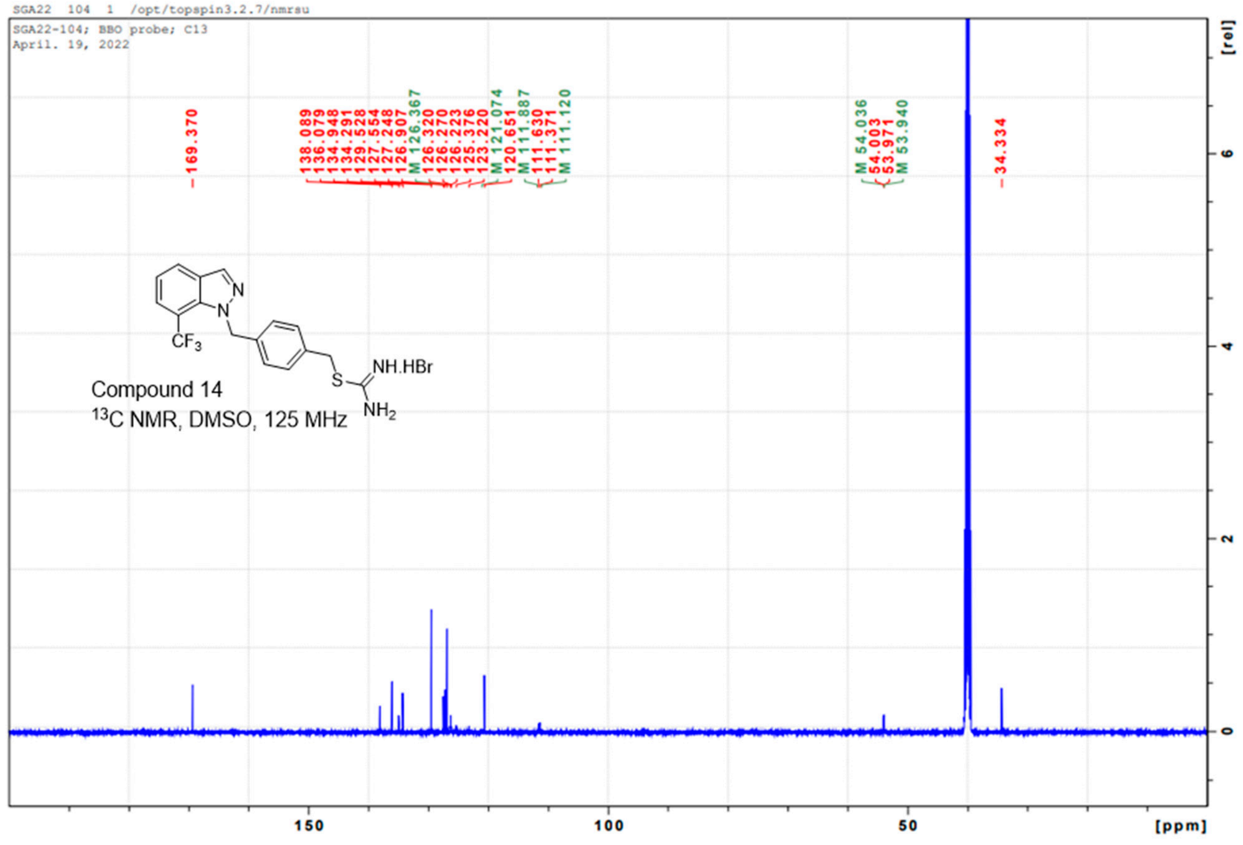

SGA22 104 1 /opt/topspin3.2.7/nmrsu
SGA22-104; BBO probe; C13
April. 19, 2022
Compound 14
^{13}C NMR, DMSO, 125 MHz
NH.HBr
NH2
CF3
[ppm]
[rel]

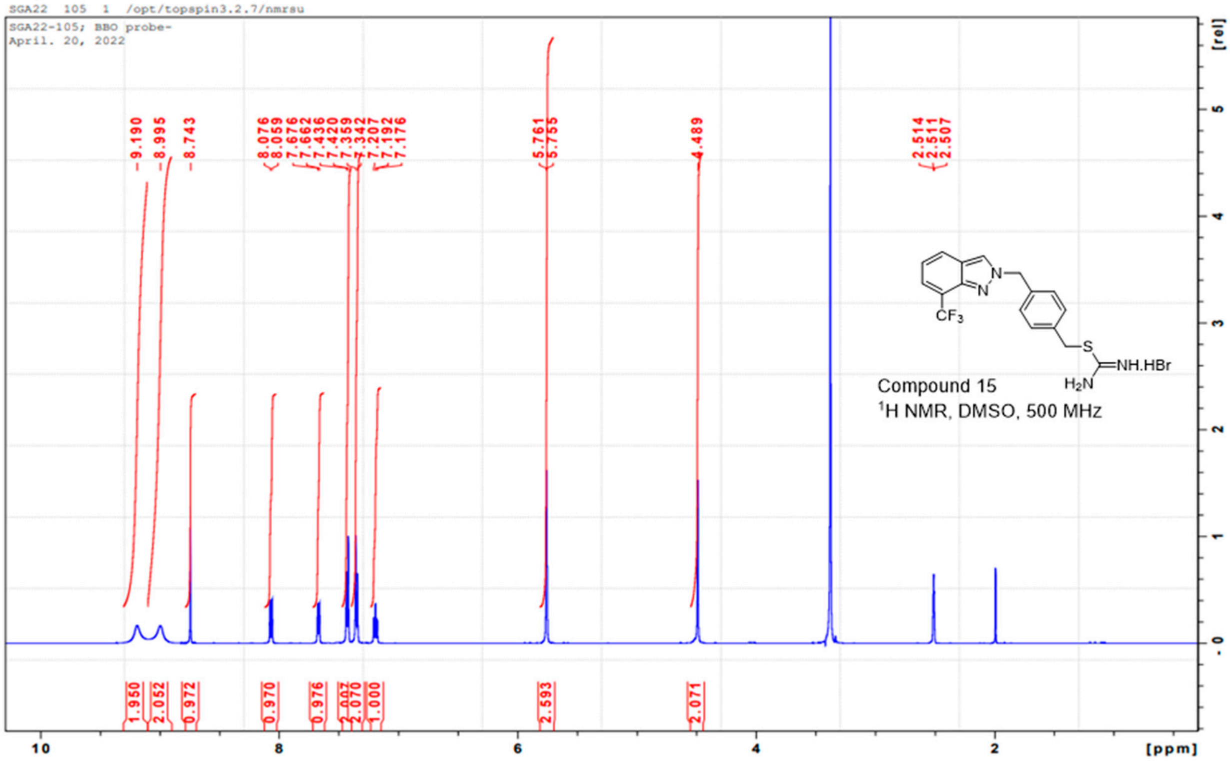
SGA22 105 1 /opt/topspin3.2.7/nmrsu
SGA22-105; BBO probe-
April. 20, 2022
9.190
8.995
8.743
8.076
8.059
7.676
7.662
7.436
7.420
7.359
7.342
7.207
7.192
7.176
5.761
5.755
4.489
2.514
2.511
2.507
N
N
CF3
S
NH.HBr
H2N
Compound 15
1H NMR, DMSO, 500 MHz
1.950
2.052
0.972
0.970
0.976
2.007
2.070
1.000
2.593
2.071
[rel]
[ppm]

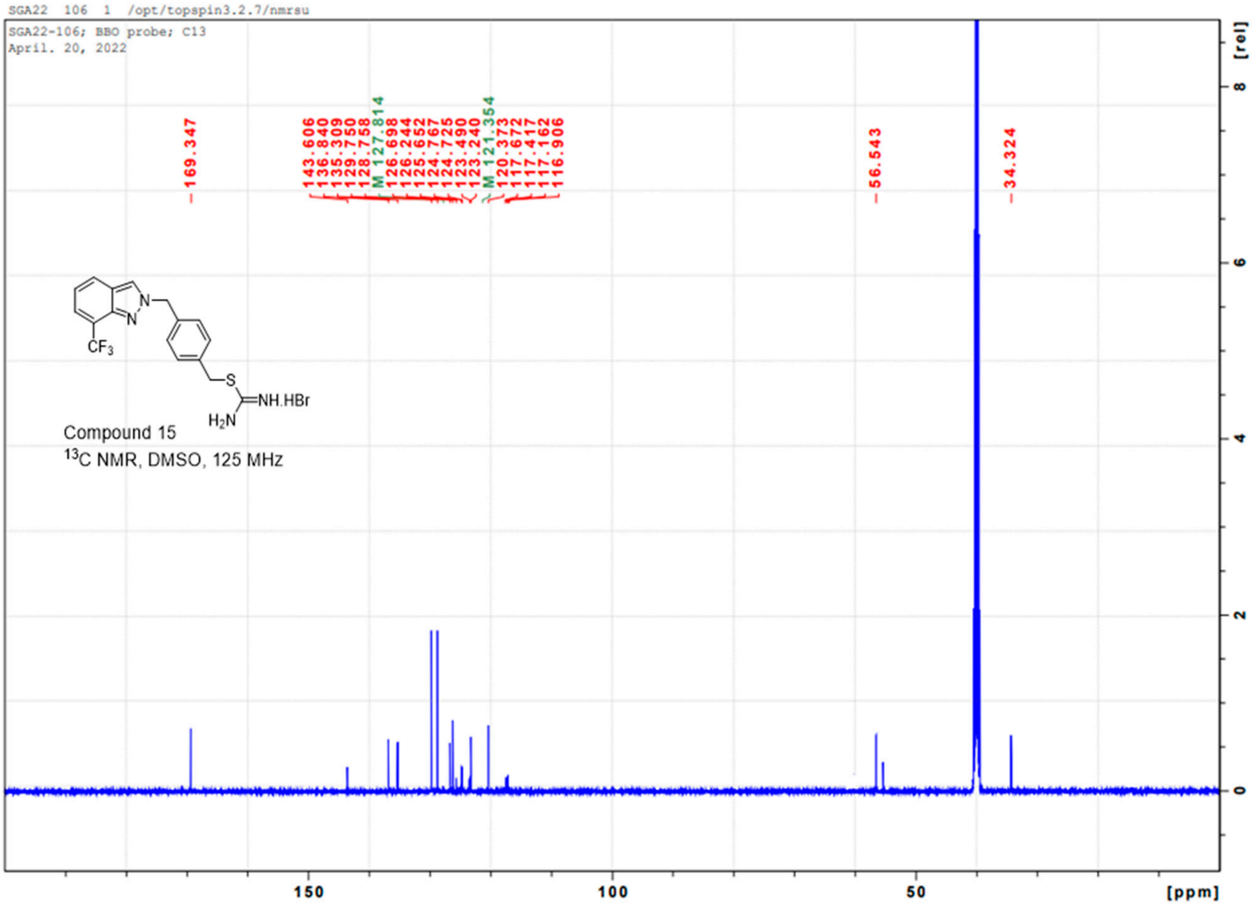
SGA22 106 1 /opt/topspin3.2.7/nmrsu
SGA22-106; BBO probe; C13
April. 20, 2022
169.347
143.606
136.840
135.309
129.750
128.758
M 127.814
126.698
126.244
125.652
124.767
124.725
123.490
123.240
M 121.354
120.373
117.672
117.417
117.162
116.906
56.543
34.324
N
N
CF3
S
NH.HBr
H2N
Compound 15
13C NMR, DMSO, 125 MHz
[rel]
[ppm]

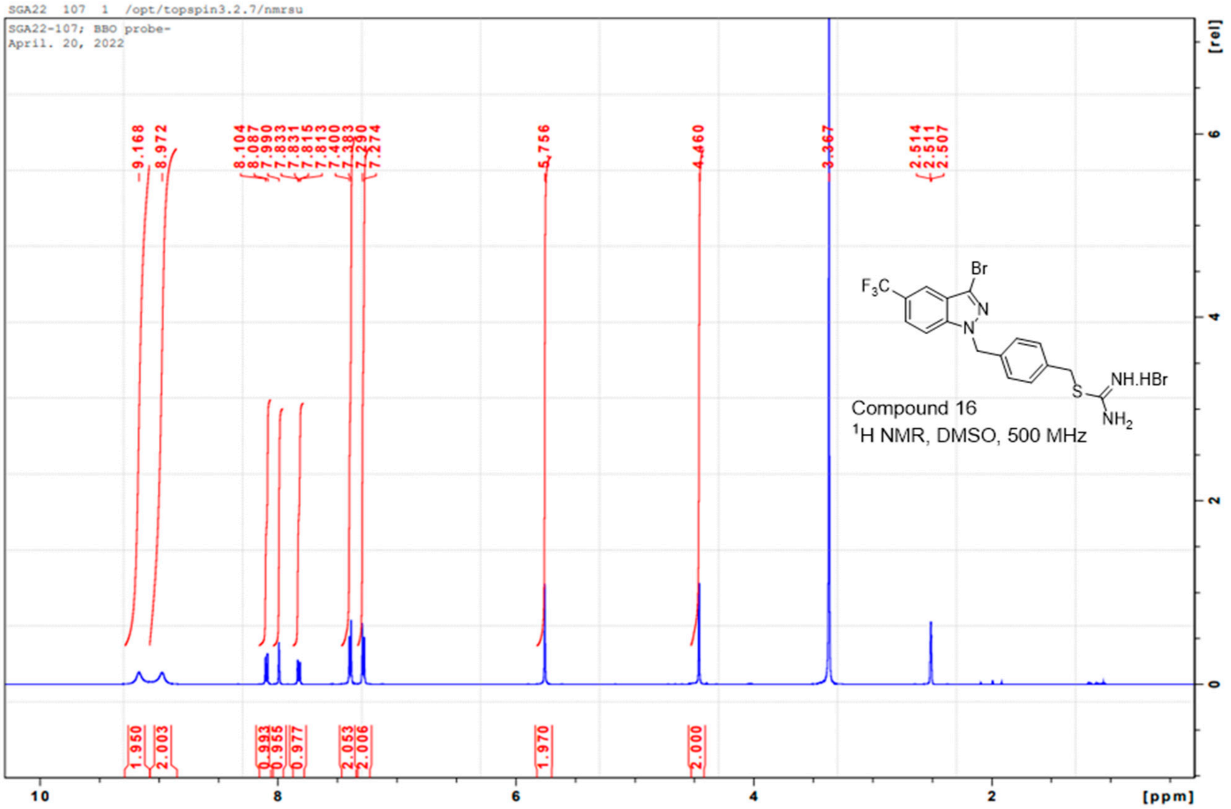
SGA22 107 1 /opt/topspin3.2.7/nmrsu
SGA22-107; BBO probe-
April. 20, 2022
9.168
8.972
8.104
8.087
7.990
7.833
7.831
7.815
7.813
7.400
7.383
7.290
7.274
5.756
4.460
3.367
2.514
2.511
2.507
1.950
2.003
0.993
0.955
0.977
2.053
2.006
1.970
2.000
Br
N
NH.HBr
S
NH_2
Compound 16
^{1}H NMR, DMSO, 500 MHz
[rel]
[ppm]

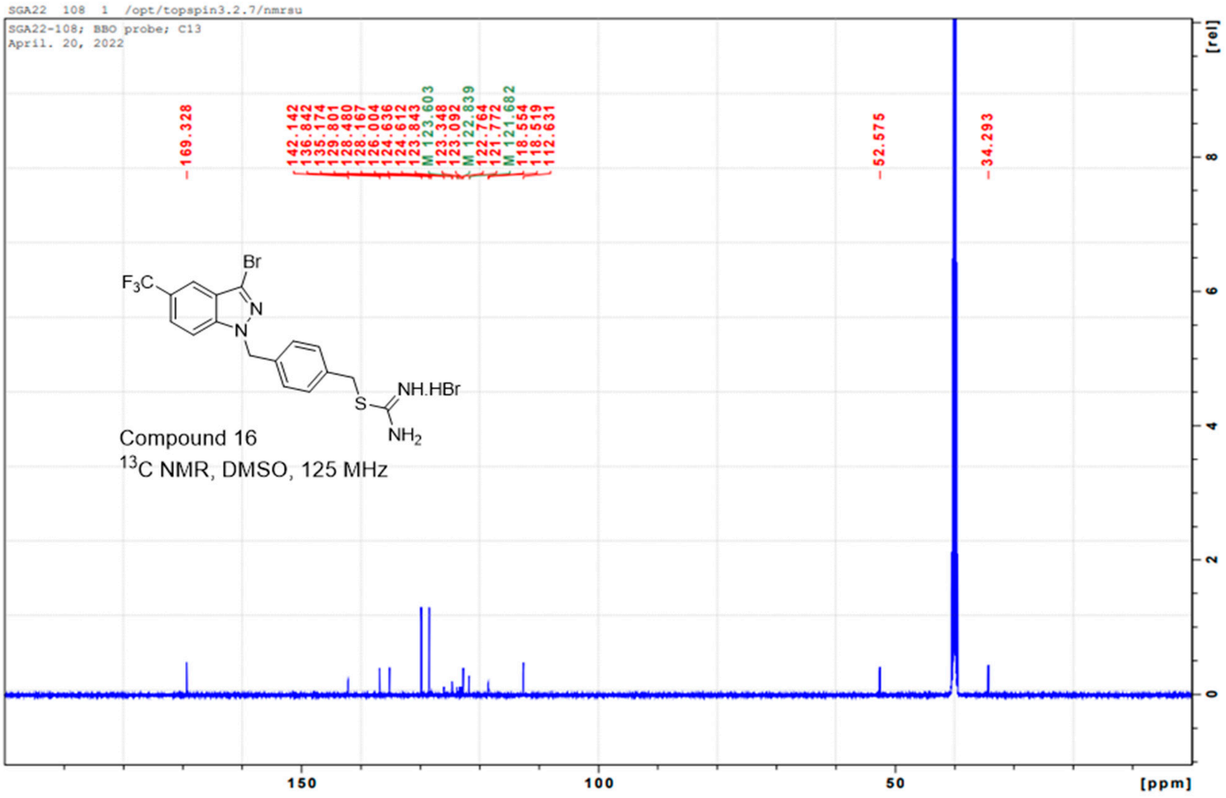
SGA22 108 1 /opt/topspin3.2.7/nmrsu
SGA22-108; BBO probe; C13
April. 20, 2022
169.328
142.142
136.842
135.174
129.801
128.480
128.167
126.004
124.636
124.612
123.843
M 123.603
123.348
123.092
M 122.839
122.764
121.772
M 121.682
118.554
118.519
112.631
52.575
34.293
Br
N
NH.HBr
S
NH_2
Compound 16
^{13}C NMR, DMSO, 125 MHz
[rel]
[ppm]

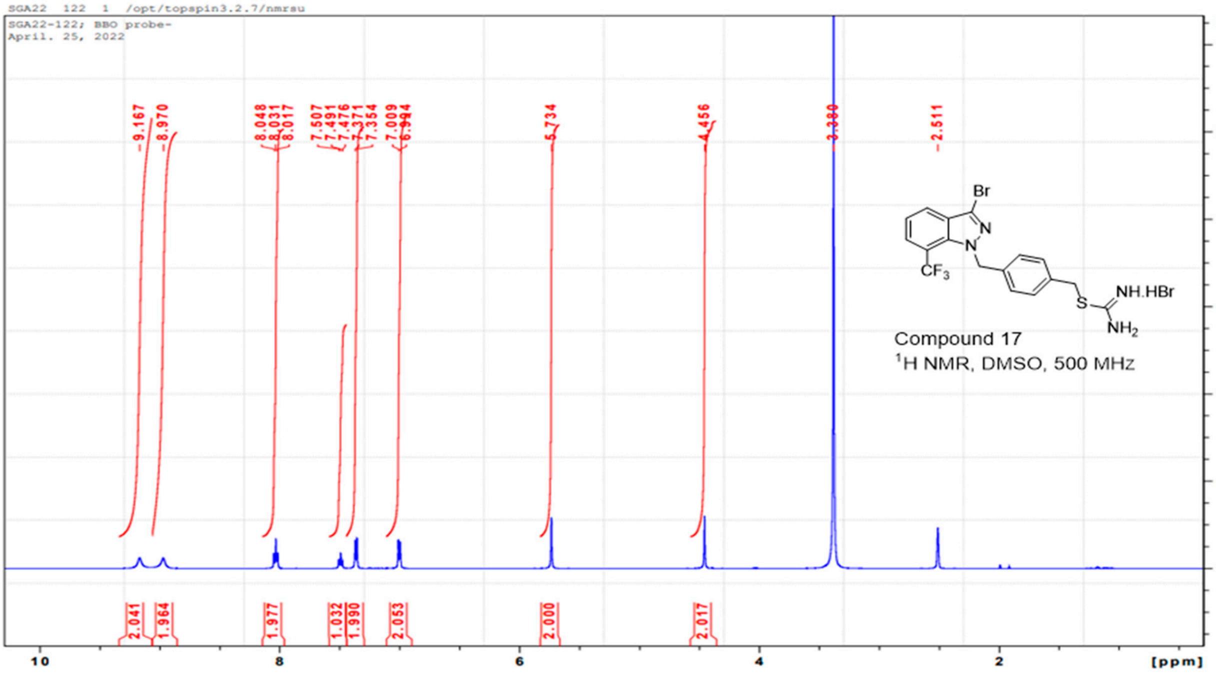
SGA22 122 1 /opt/topspin3.2.7/nmrsu
SGA22-122; BBO probe-
April. 25, 2022
Compound 17
^{1}H NMR, DMSO, 500 MHz
Br
N
N
CF3
NH.HBr
S
NH2
9.167
8.970
8.048
8.031
8.017
7.507
7.491
7.476
7.371
7.354
7.009
6.994
5.734
4.456
3.380
2.511
2.041
1.964
1.977
1.032
1.990
2.053
2.000
2.017
[ppm]
[rel]

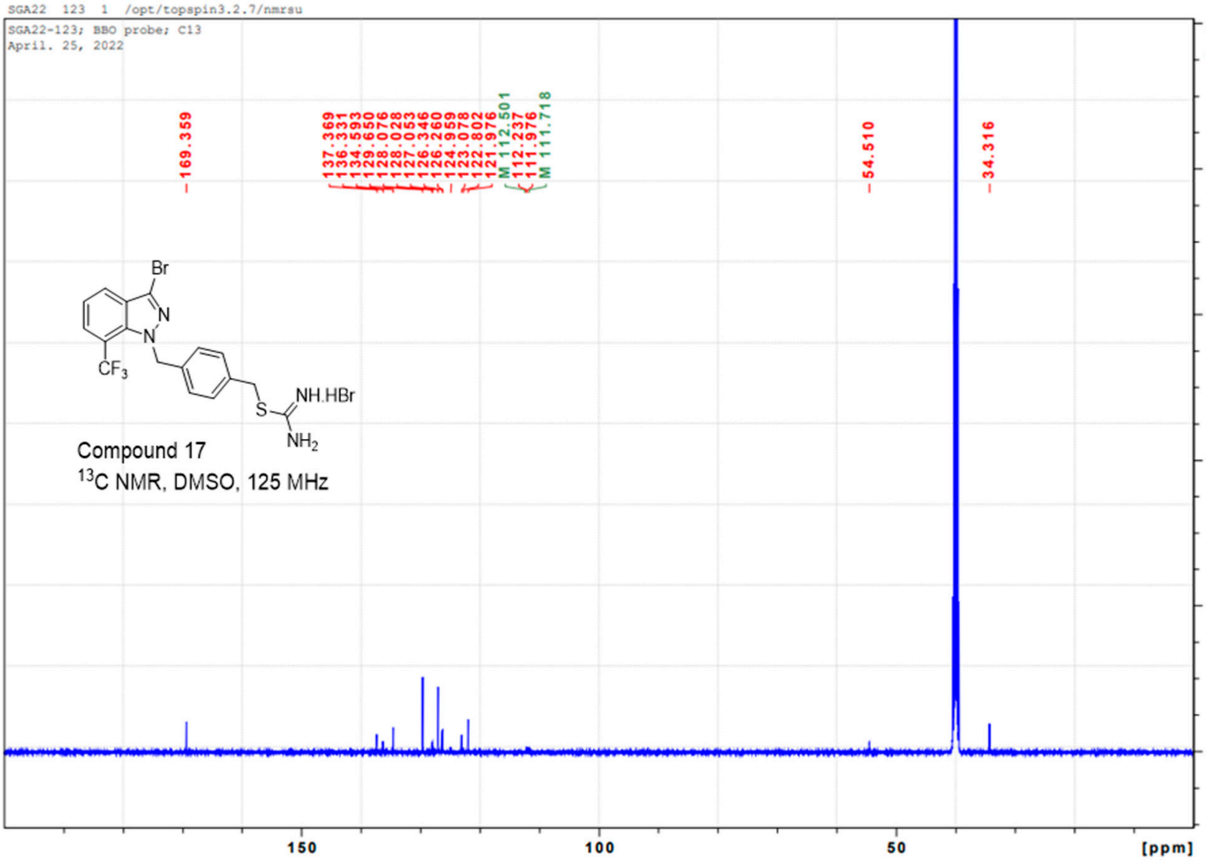
SGA22 123 1 /opt/topspin3.2.7/nmrsu
SGA22-123; BBO probe; C13
April. 25, 2022
Compound 17
^{13}C NMR, DMSO, 125 MHz
Br
N
N
CF3
NH.HBr
S
NH2
169.359
137.369
136.331
134.593
129.650
128.076
128.028
127.053
126.346
126.260
124.959
123.078
122.802
121.976
M 112.501
112.237
111.976
M 111.718
54.510
34.316
[ppm]
[rel]

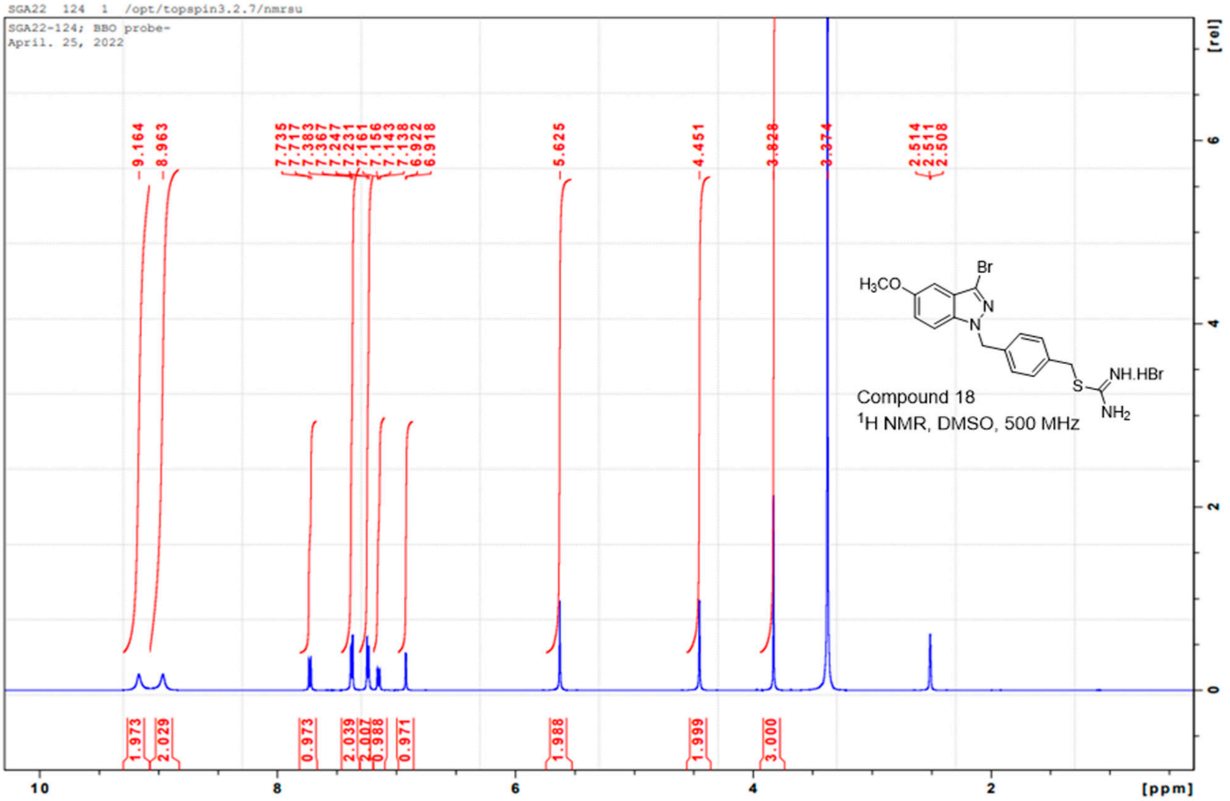
SGA22 124 1 /opt/topspin3.2.7/nmrsu
SGA22-124; BBO probe-
April. 25, 2022
Compound 18
^{1}H NMR, DMSO, 500 MHz
[ppm]
[rel]

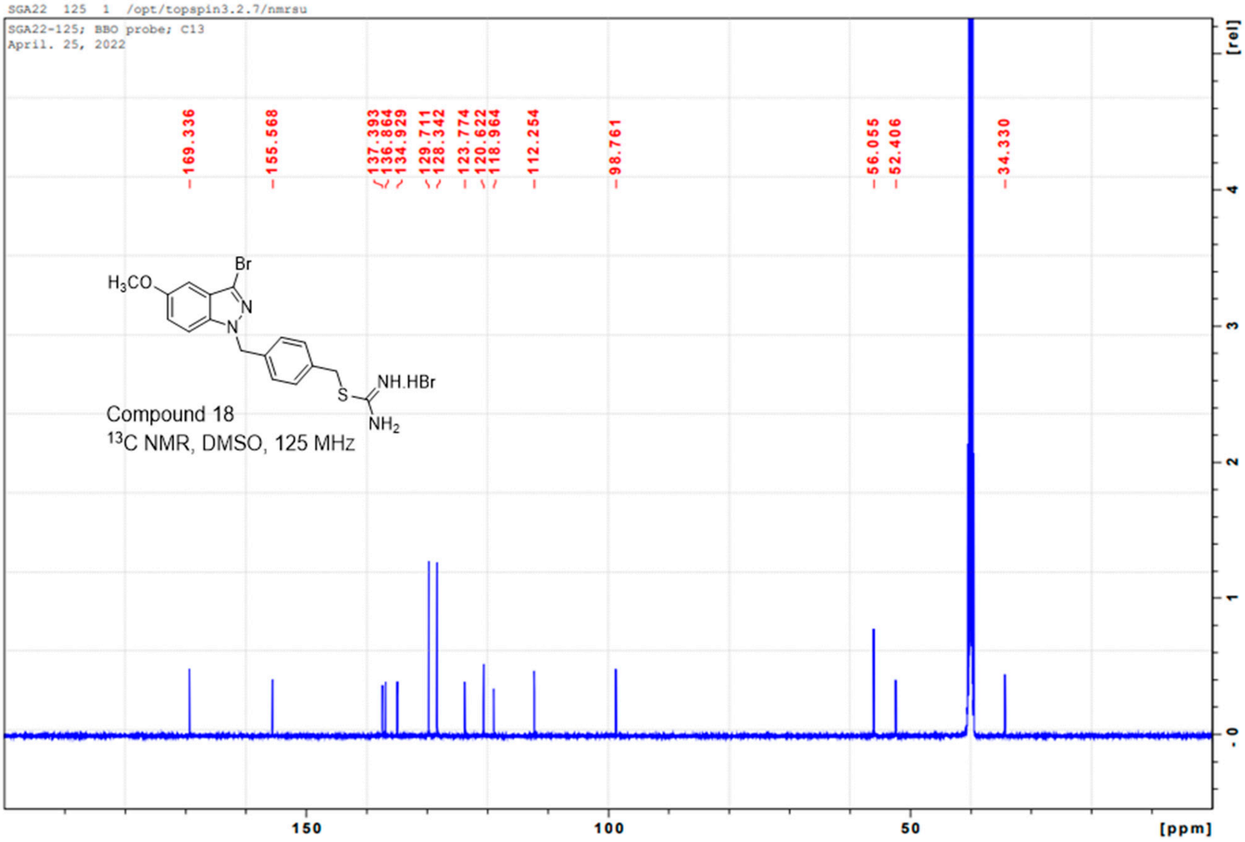
SGA22 125 1 /opt/topspin3.2.7/nmrsu
SGA22-125; BBO probe; C13
April. 25, 2022
Compound 18
^{13}C NMR, DMSO, 125 MHz
[ppm]
[rel]

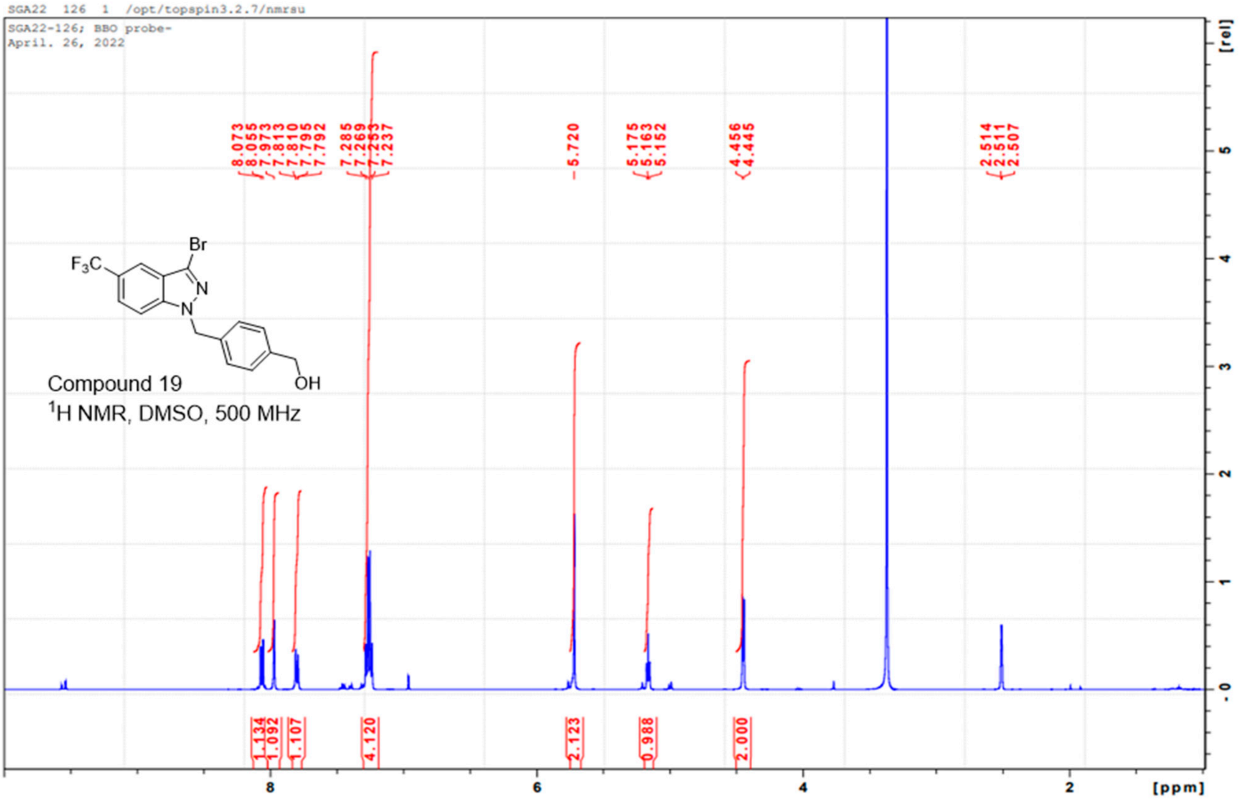
SGA22 126 1 /opt/topspin3.2.7/nmrsu
SGA22-126; BBO probe-
April. 26, 2022
Compound 19
¹H NMR, DMSO, 500 MHz
[ppm]
[rel]

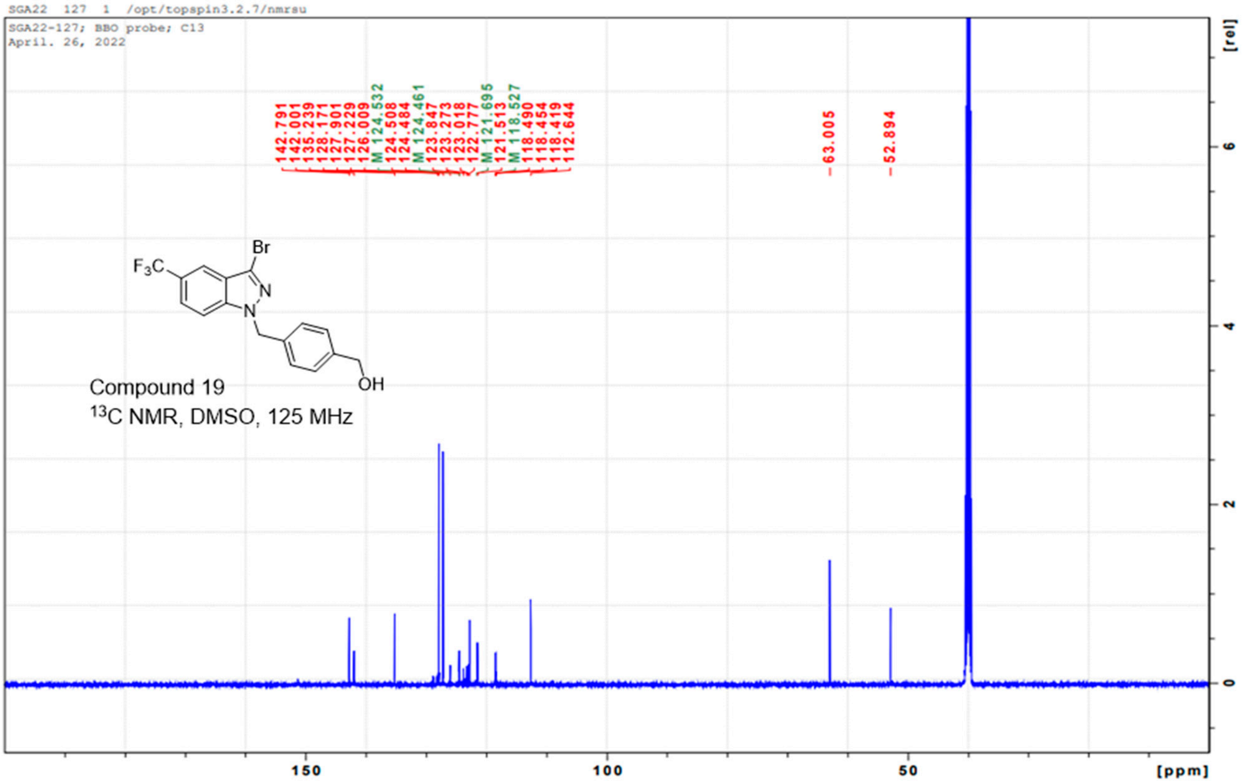
SGA22 127 1 /opt/topspin3.2.7/nmrsu
SGA22-127; BBO probe; C13
April. 26, 2022
Compound 19
¹³C NMR, DMSO, 125 MHz
[ppm]
[rel]

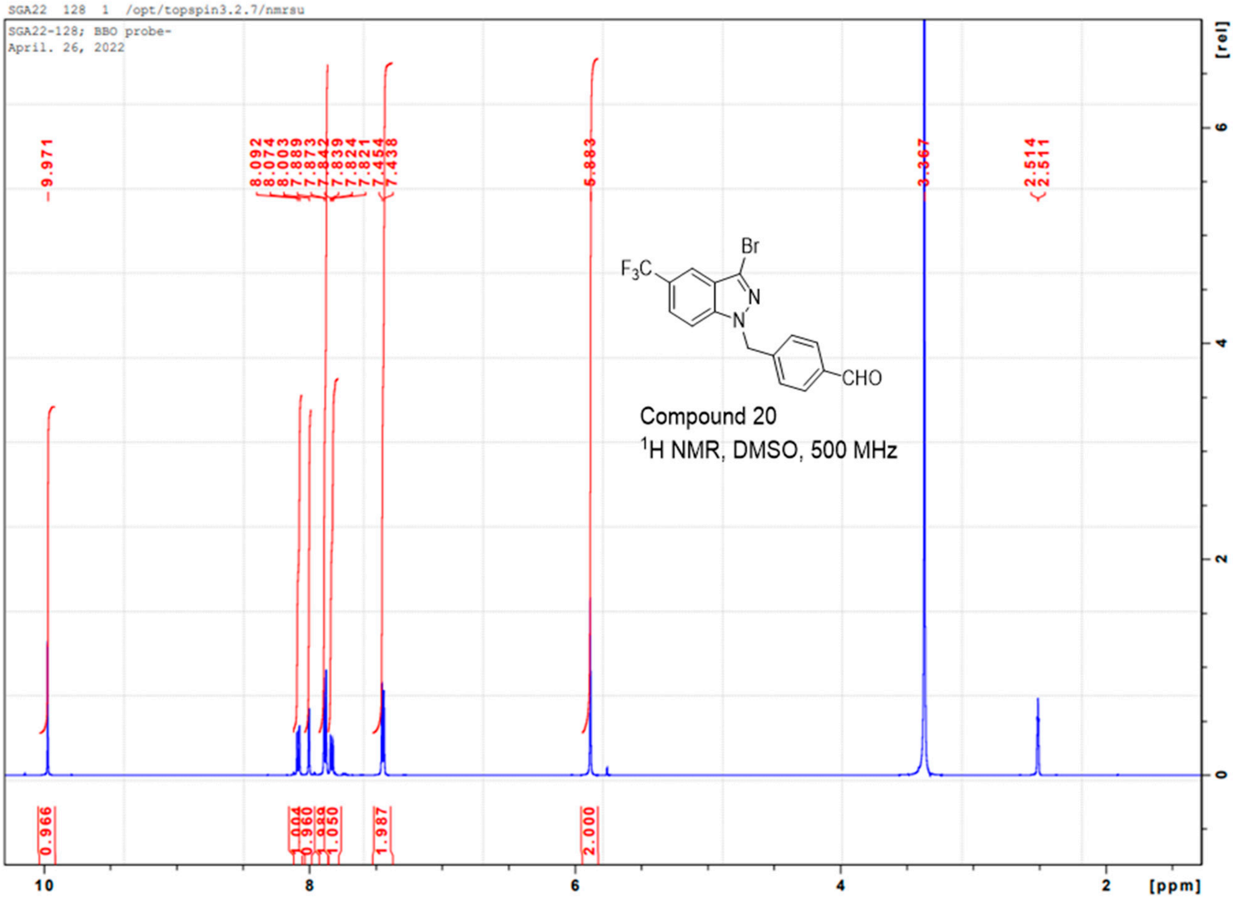
SGA22 128 1 /opt/topspin3.2.7/nmrsu
SGA22-128; BBO probe-
April. 26, 2022
Compound 20
1H NMR, DMSO, 500 MHz
[ppm]
[rel]

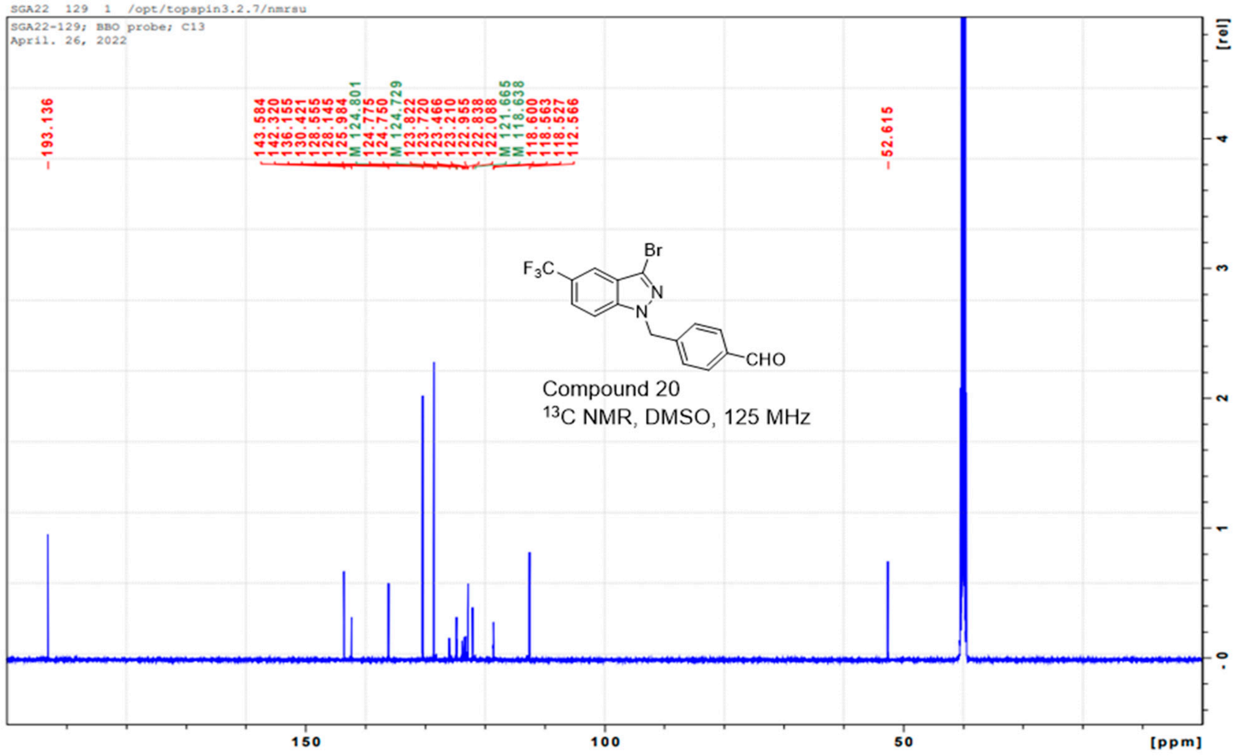
SGA22 129 1 /opt/topspin3.2.7/nmrsu
SGA22-129; BBO probe; C13
April. 26, 2022
Compound 20
13C NMR, DMSO, 125 MHz
[ppm]
[rel]

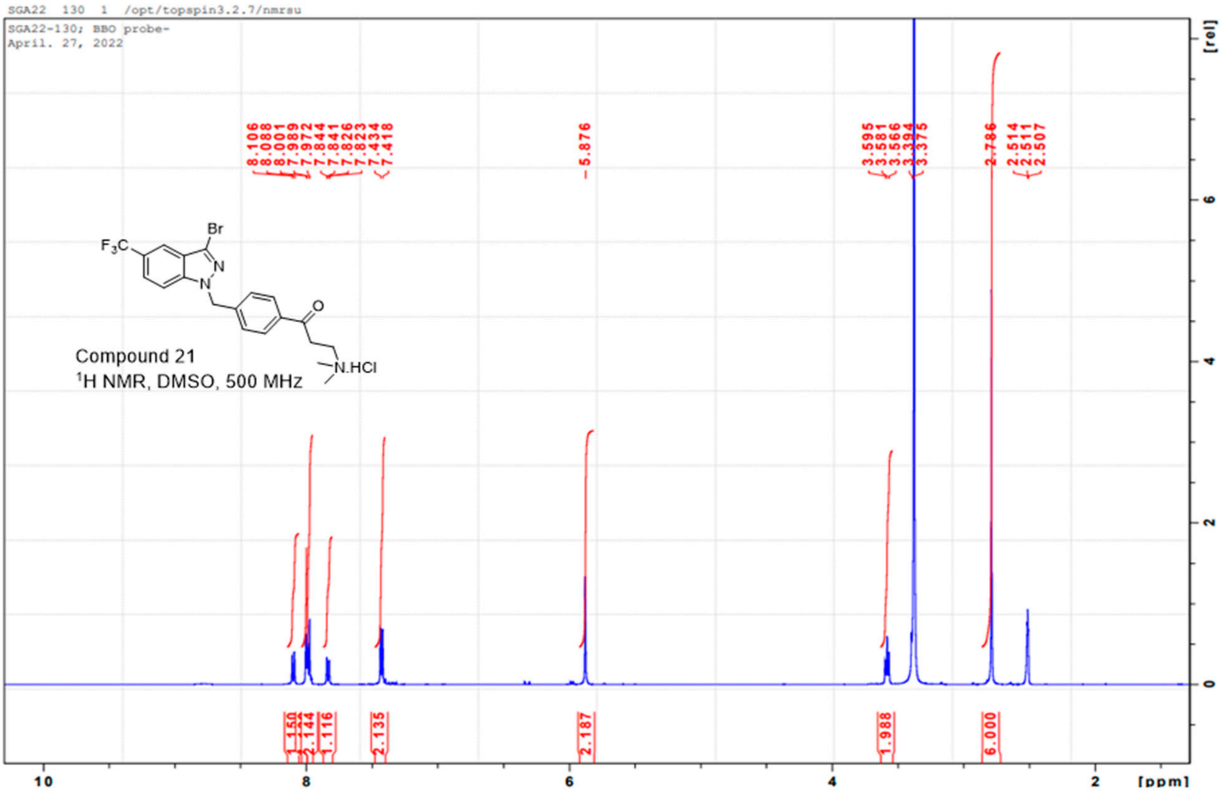
SGA22 130 1 /opt/topspin3.2.7/nmrsu
SGA22-130; BBO probe-
April. 27, 2022
8.106
8.088
8.001
7.989
7.972
7.844
7.841
7.826
7.823
7.434
7.418
5.876
3.595
3.581
3.566
3.394
3.375
2.786
2.514
2.511
2.507
Br
F3C
N
N
O
N.HCl
Compound 21
1H NMR, DMSO, 500 MHz
1.150
2.144
1.116
2.135
2.187
1.988
6.000
[rel]
[ppm]

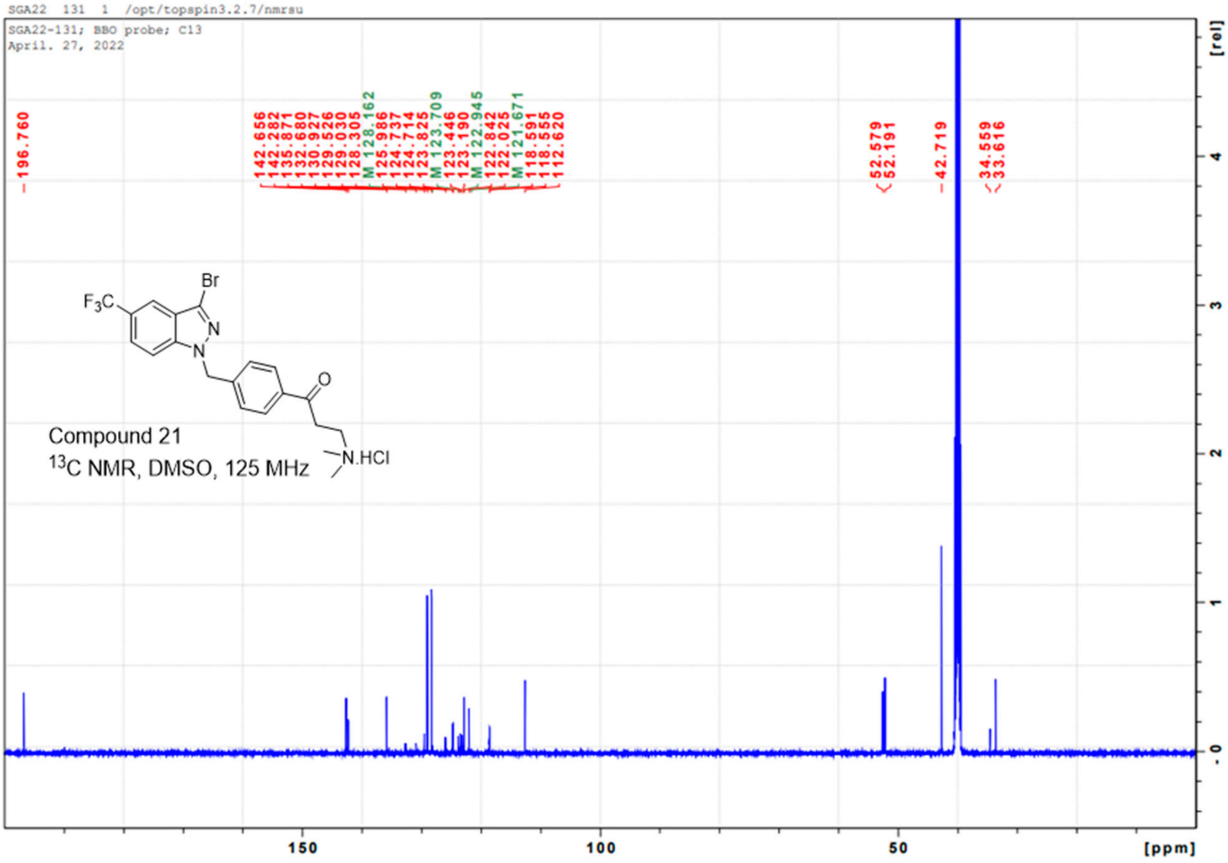
SGA22 131 1 /opt/topspin3.2.7/nmrsu
SGA22-131; BBO probe; C13
April. 27, 2022
196.760
142.656
142.282
135.871
132.680
130.927
129.526
129.030
128.305
M 128.162
125.986
124.737
124.714
123.825
M 123.709
123.446
123.190
M 122.945
122.842
122.025
M 121.671
118.591
118.555
112.620
52.579
52.191
42.719
34.559
33.616
Br
F3C
N
N
O
N.HCl
Compound 21
13C NMR, DMSO, 125 MHz
[rel]
[ppm]
